# Characterizing Anchoring Bias in Vaccine Comparator Selection Due to Health Care Utilization With COVID-19 and Influenza: Observational Cohort Study

**DOI:** 10.2196/33099

**Published:** 2022-06-17

**Authors:** Anna Ostropolets, Patrick B Ryan, Martijn J Schuemie, George Hripcsak

**Affiliations:** 1 Department of Biomedical Informatics Columbia University Irving Medical Center New York, NY United States; 2 Epidemiology Analytics Janssen Research and Development Titusville, NJ United States; 3 Medical Informatics Services New York-Presbyterian Hospital New York, NY United States

**Keywords:** COVID-19, vaccine, anchoring, comparator selection, time-at-risk, vaccination, bias, observational, utilization, flu, influenza, index, cohort

## Abstract

**Background:**

Observational data enables large-scale vaccine safety surveillance but requires careful evaluation of the potential sources of bias. One potential source of bias is the index date selection procedure for the unvaccinated cohort or unvaccinated comparison time (“anchoring”).

**Objective:**

Here, we evaluated the different index date selection procedures for 2 vaccinations: COVID-19 and influenza.

**Methods:**

For each vaccine, we extracted patient baseline characteristics on the index date and up to 450 days prior and then compared them to the characteristics of the unvaccinated patients indexed on (1) an arbitrary date or (2) a date of a visit. Additionally, we compared vaccinated patients indexed on the date of vaccination and the same patients indexed on a prior date or visit.

**Results:**

COVID-19 vaccination and influenza vaccination differ drastically from each other in terms of the populations vaccinated and their status on the day of vaccination. When compared to indexing on a visit in the unvaccinated population, influenza vaccination had markedly higher covariate proportions, and COVID-19 vaccination had lower proportions of most covariates on the index date. In contrast, COVID-19 vaccination had similar covariate proportions when compared to an arbitrary date. These effects attenuated, but were still present, with a longer lookback period. The effect of day 0 was present even when the patients served as their own controls.

**Conclusions:**

Patient baseline characteristics are sensitive to the choice of the index date. In vaccine safety studies, unexposed index event should represent vaccination settings. Study designs previously used to assess influenza vaccination must be reassessed for COVID-19 to account for a potentially healthier population and lack of medical activity on the day of vaccination.

## Introduction

The world is faced with a deadly pandemic at a time of incredible technology such that new vaccines can be produced in a fraction of the previous development time and at a scale that can potentially vaccinate the entire human population. This brings new challenges in using observational data to evaluate vaccine safety, where the pressure to vaccinate quickly to prevent more deaths and viral variants reduces the time available to carry out studies [1]. This time pressure affects not just the collection of data for research but also the time it takes to develop and validate the evaluation methods. We therefore rely on the methods developed and validated in previous pandemics and seasonal infectious diseases, with influenza being an important example [2-4].

COVID-19 vaccination has been unlike any other in history. The target vaccination group has shifted from older adults and those with comorbidities in the early phases of vaccination to everyone including healthy, young people [5], with some nations already vaccinating the majority of their populations [6]. COVID-19 vaccines are delivered in a wide variety of settings, from pop-up centers unconnected to health care delivery to inpatient facilities for hospital discharge. Other vaccines such as those for influenza have a different delivery. They are often administered to specific vulnerable populations, such as pregnant women, patients at high risk of complications, or children, and are often given during health care visits [7-9].

The unique properties of COVID-19 vaccination may require adjusting study designs previously used for influenza vaccination, specifically the selection of a comparator cohort or an unvaccinated comparison time in cohort and self-controlled studies. Although, for the vaccinated group, the index date—vaccination—is clearly defined, the selection of the index date for the unexposed comparator group is more complex. Ideally, the index date in the unexposed group should be chosen based on the vaccination settings to reliably serve as a counterfactual. The selection procedure (which we have termed “anchoring”) may itself influence the results of a study and induce bias in the analysis. For example, in studies of the background rates of adverse events, patients indexed on an arbitrary date were shown to have lower incidence of adverse events than the same patients indexed on a visit [10].

Here, we aimed to evaluate 2 alternative selection procedures for the index date in the unexposed group based on how vaccines are administered—coupled or decoupled to another health care encounter. We compared these approaches for 2 vaccinations, influenza and COVID-19, and investigated how anchoring influences the baseline patient characteristics of the unexposed group.

## Methods

### Data Collection and Analysis

We studied 2 types of vaccination: (1) influenza vaccine administered from 2017-2018 and (2) COVID-19 vaccine administered from 2020-2021 (the list of codes is presented in Table S1 in Multimedia Appendix 1). For each vaccine, we mimicked 2 study designs.

**Figure 1:**
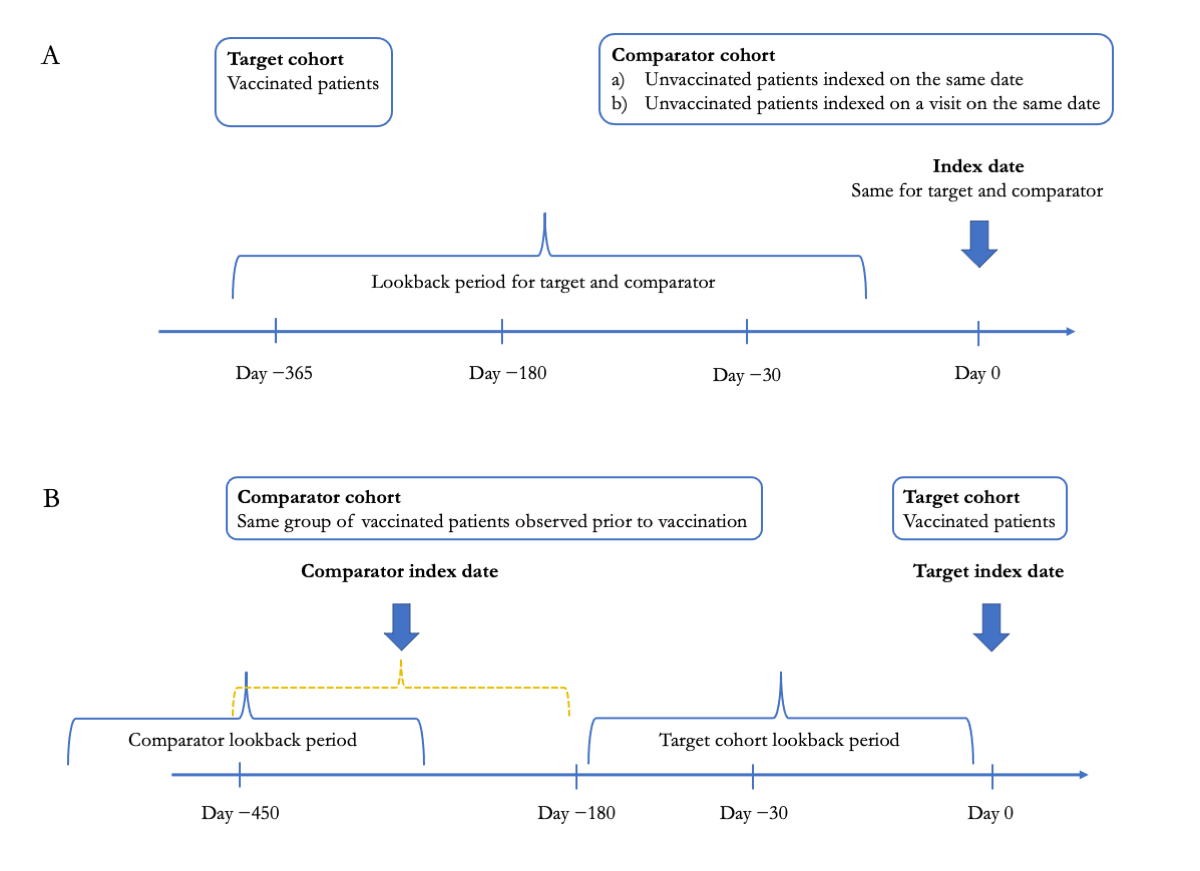
Study design overview.

The first design (Figure 1A) corresponds to a cohort method, where the target group was vaccinated patients and the comparator group was unvaccinated patients. The index date for the target group was the date of vaccination; for the comparator, it was (1) a date selected from the unvaccinated patient’s history (not necessarily with any medical event) such that it matched the index date of one of the target group participants or (2) a visit matched to the index date of one of the target group participants. Patients in each target and comparator pair were matched on age and gender.

The second design (Figure 1B) corresponds to a self-controlled design (case-crossover design) [11], where the cases were the vaccinated patients indexed (or “anchored”) on the day of vaccination and the controls were the same patients indexed on an arbitrary date or a visit within 180-450 days prior to the vaccination date.

For each group, we extracted patient baseline characteristics (covariates) recorded within 5 time intervals: on the index date (day 0), on the day before the index date (day –1), from 30 to 1 days prior to the index date (short-term baseline covariates), from 180 to 31 days prior to the index date (medium-term baseline covariates), and from 450 to 181 days prior to the index date (long-term baseline covariates). Baseline covariates included all condition, procedure, measurement (laboratory tests and vital signs), and drug group codes available in the patients’ structured data within a specified time interval. For each covariate, we calculated covariate proportion, which is the proportion of patients with a covariate recorded in their electronic health record (EHR) within a given time interval along with its SD for binary variables or an average number with SD for continuous variables (such as the number of visits).

We then compared the covariates in each target-comparator pair and calculated the standardized difference of means. The covariates were said to be balanced if the standardized difference of means was less than 0.1 [12,13]. The standardized difference of means for each covariate was then plotted for each time interval and target-comparator pair.

We conducted the analysis on 2 EHR data sources: Columbia University Irving Medical Center health record data set (CUIMC) and Optum deidentified electronic health record data set (Optum EHR). Optum EHR’s data comprises medical record data from 87 million patients and includes clinical information, inclusive of prescriptions as prescribed and administered, lab results, vital signs, body measurements, diagnoses, and procedures. The CUIMC EHR gathers data from the clinical data warehouse of the NewYork-Presbyterian Hospital/Columbia University Irving Medical Center, New York, NY, based on its current and previous EHR systems, with data spanning over 30 years and including over 6 million patients. The data sources were selected based on the availability of both vaccines’ data and captured inpatient and ambulatory aspects of care. Both data sources were mapped to the Observational Medical Outcomes Partnership Common Data Model [14]. The Observational Medical Outcomes Partnership Common Data Model provides a homogeneous format for health care data and standardization of the underlying clinical coding systems that thus enables analysis code to be shared across participating data sets in the network.

All analysis was done in R statistical software (version 4; R Foundation for Statistical Computing). FeatureExtraction package (version 3.1; Observational Health Data Sciences and Informatics) was used to extract the baseline covariates.

### Ethics Approval

The protocol for this research was approved by the Columbia University Institutional Review Board (AAAO7805).

## Results

### Study Populations

The initial study population included 210,263 and 57,000 patients vaccinated with any COVID-19 vaccine from 2020-2021 in CUIMC and Optum EHR, respectively, and 60,142 and 4,991,051 patients vaccinated with an influenza vaccine from 2017-2018 in CUIMC and Optum EHR, respectively. The proportion of female patients was 62.7% (131,922/210,263) and 72.3% (41,204/57,000) for COVID-19 vaccinated patients and 61.4% (36,917/60,142) and 58.2% (2,906,757/4,991,051) for influenza vaccinated patients. The median (IQR) age was 57 (39-71) and 45 (34-56) years for COVID-19 vaccinated patients and 35 (12-63) and 50 (22-66) years for influenza vaccinated patients. We then matched each vaccinated population to the unvaccinated population on the date, age, and gender so that the distribution of age and gender between each target and comparator group was the same.

**Table 1:** The number of covariates with the standardized difference of means >0.1 for selected time intervals.

Target-comparator pair	Index date (day 0), n/N (%)		Long-term (from 450-181 days prior to the index date), n/N (%)	
	CUIMC^a^	Optum EHR^b^	CUIMC	Optum EHR
COVID-19–vaccinated patients compared to unvaccinated patients indexed on a date	25/9073 (0.3)	11/15,097 (<0.1)	131/26,859 (0.5)	56/51,075 (0.1)
COVID-19–vaccinated patients compared to unvaccinated patients indexed on a visit	411/18,741 (2.2)	110/21,739 (0.5)	34/37,073 (<0.1)	91/50,358 (0.2)
Influenza-vaccinated patients compared to unvaccinated patients indexed on a date	469/12,684 (3.7)	268/26,809 (1)	881/25,782 (3.4)	201/55,665 (0.4)
Influenza-vaccinated patients compared to unvaccinated patients indexed on a visit	320/22,816 (1.4)	94/32,931 (0.3)	517/34,361 (1.5)	114/56,387 (0.2)

^a^CUIMC: Columbia University Irving Medical Center electronic health record data set.

^b^Optum EHR: Optum electronic health record data set.

### Comparison of Vaccinated Patients and Unvaccinated Patients Indexed on a Date or a Visit

#### Influenza-Vaccinated Population

**Figure 2:**
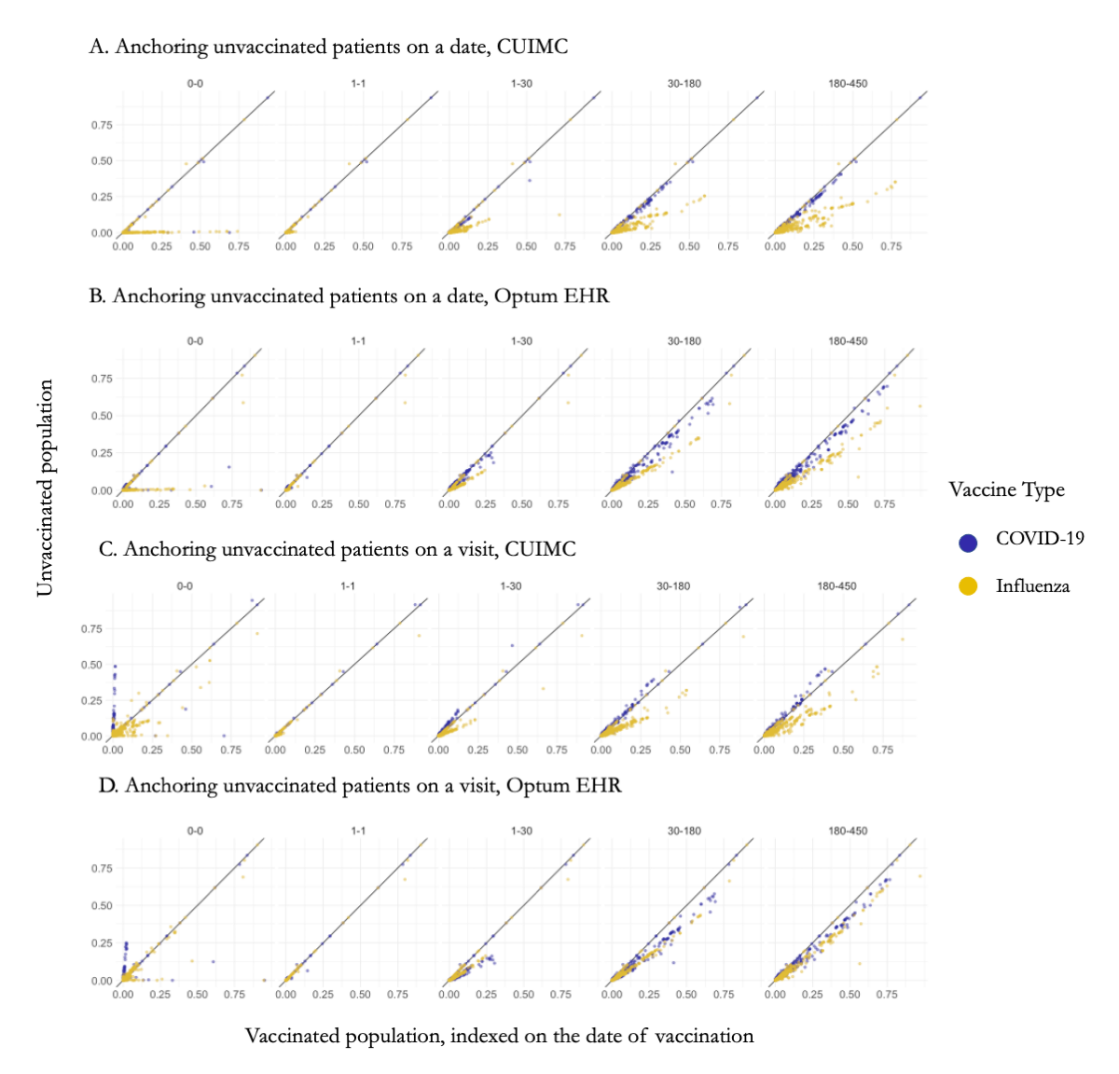
Baseline covariate proportion in vaccinated and unvaccinated populations on day 0, day –1, days –1 to –30, days –31 to –180, and days –181 to –450 in CUIMC and Optum EHR. Each dot represents a covariate. Blue: covariate proportion in COVID-19 vaccinated population versus unvaccinated population; yellow: in influenza vaccinated population versus unvaccinated population. CUIMC: Columbia University Medical Irving Center health record data set; Optum EHR: Optum electronic health record data set.

On the index date (day of vaccination=day 0), the influenza-vaccinated population had markedly higher proportion of most covariates than an arbitrary date in the comparison group (pinning most covariates against the x-axis in Figure 2, A and B, yellow). The largest difference in covariate proportions between the unvaccinated and vaccinated populations on day 0 was observed for inpatient and outpatient measurements such as blood count, metabolic panels, blood pressure, and basal metabolic index, including both the presence of measurements and proportion of patients with of abnormal results; this means that patients were far more likely to have measurements on the date of vaccination than on an arbitrary date. Moreover, the influenza-vaccinated population had higher covariate proportions even a year prior to the vaccination.

In contrast, comparison with the unvaccinated population indexed on a visit (Figure 2, C and D) showed a smaller difference between covariate proportions in CUIMC and almost no difference in Optum EHR, potentially indicating that a visit is a better counterfactual for a vaccination date than an arbitrary date.

Covariate proportions in vaccinated patients were closer to the proportions in the unvaccinated population indexed on a visit even with a longer lookback period (examples of covariates are provided in Multimedia Appendix 1).

#### COVID-19–Vaccinated Population

As opposed to the influenza-vaccinated population, the difference in covariate proportion between the COVID-19–vaccinated and unvaccinated population indexed on an arbitrary date was moderate. We observed that COVID-19 vaccination was associated with a visit in 2.7% (5732/210,263) of patients (compared to 1.2% [2591/210,263] on an arbitrary date). In contrast, 55.8% (33,531/60,142) of the influenza-vaccinated population had a visit on the date of vaccination (compared to 0.6% [331/60,142] of unvaccinated population on an arbitrary date). The vaccinated population tended to have higher proportion of covariates prior to the index date (looking back a year prior).

When compared to the unvaccinated population indexed on a visit, the COVID-19–vaccinated population had markedly lower proportion of most covariates. Those vaccinated with the COVID-19 vaccine had much lower rates of diagnoses of both chronic and acute diseases on the date of vaccination than a visit in the unvaccinated population. The list of conditions included common chronic conditions such as hypertension, depressive disorder, asthma, and diabetes mellitus along with acute conditions such as dyspnea, chest pain, and fever. This difference points out that an arbitrary date may be a better counterfactual for a vaccination date in COVID-19–vaccinated patients.

### Comparison of Vaccinated Patients Indexed on the Date of Vaccination and the Same Patients Indexed on a Prior Date or Visit

#### Influenza-Vaccinated Population

**Figure 3:**
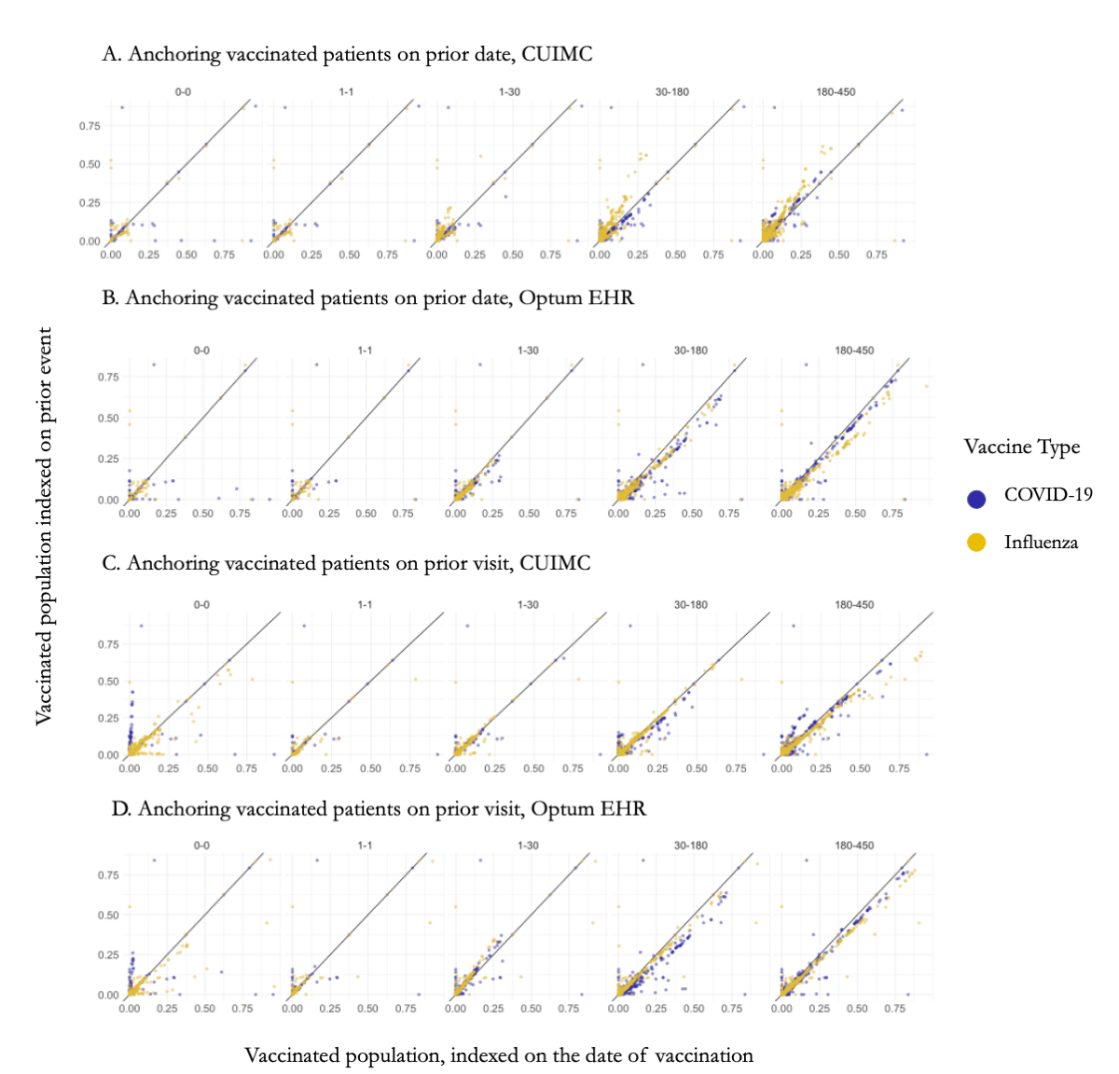
Baseline covariate proportion in vaccinated population indexed on the date of vaccination compared to the same population indexed on a prior visit or date on day 0, day –1, days –1 to –30, days –31 to –180, and days –181 to –450 in CUIMC and Optum EHR. Each dot represents a covariate. Blue: covariate proportion in COVID-19 population; yellow: in influenza vaccinated population. CUIMC: Columbia University Irving Medical Center health record data set; Optum EHR: Optum electronic health record data set.

Here, we compared vaccinated patients indexed on the vaccination date to the same patients indexed on a date or visit within a year prior, similar to the procedure in a self-controlled study. We observed that the date of influenza vaccination tended to have a higher proportion of covariates than an arbitrary date within a year prior (Figure 3, first column) and even higher than an arbitrary visit within a year prior. Patients indexed on the date of vaccination were more likely to have antecedent health care encounters, conditions, and laboratory tests within a year prior to the vaccination date than within a year prior to their previous visits (Figure 3, C and D). For comparison with an arbitrary date, we observed a mixed effect: in Optum EHR, vaccinated patients had more events preceding their vaccination, whereas in CUIMC they had fewer events. Nevertheless, in both data sources, the difference between covariate proportions was larger in magnitude when compared to an arbitrary date than when compared to an arbitrary visit.

#### COVID-19–Vaccinated Population

The COVID-19–vaccinated population showed a markedly lower proportion of covariates on the day of vaccination than a visit or an arbitrary date within a year prior to vaccination. The difference was attenuated with a longer lookback period; COVID-19–vaccinated patients had fewer health care events within a year prior to their vaccination than their previous history. We observed mixed effect when comparing to a date in the past; some covariates such as exposure to COVID-19, COVID-19 laboratory tests, vital signs, or acetaminophen were present in a higher proportion immediately before vaccination. Others such as glomerular filtration rate, thyrotropin measurement, urinalysis, or glomerulonephritis were observed in a lower proportion immediately before the vaccination.

## Discussion

### Principal Findings

We find that COVID-19 vaccination and influenza vaccination differ drastically from each other, with the proportion of most covariates much higher on the date of vaccination in the influenza group than the COVID-19 group. The results from looking back from 31 to 180 days and from 181 to 450 days before the vaccination (or index date) may be related to differences in the populations. The population vaccinated for influenza appears to have more comorbidities and past procedures and measurements than the average population, even after adjusting for age and gender, and the population vaccinated for COVID-19 appears to have a lower proportion of most medical covariates than the average population after adjusting for age and gender. This may be explained if influenza vaccination is targeted to sicker populations on average and if COVID-19 vaccination is targeted to the general public, which is healthier on average than those in our EHR data [7,9].

The drastic effects on day 0 (ie, the day of vaccination and its comparison) are likely related to the context in which the vaccination is given. If the comparison is an arbitrary date in the person’s record, then influenza vaccination has markedly higher covariate proportions, reflecting the association of the vaccination with a health care encounter. Moreover, such a trend (not observed for the COVID-19 vaccine) was present even when comparing the date of influenza vaccination to prior patient visits.

The abovementioned trends for the COVID-19 vaccine were consistently observed in both data sources, and the differences between the data sources were mainly related to the coding practices. For example, in the CUIMC data, COVID-19 vaccination was not associated with a visit but rather with a patient encounter. On the contrary, COVID-19 vaccination in the Optum EHR was associated with the providers entering “Requires vaccination” and “Vaccine Administration” in the system along with the codes for the vaccines. For influenza vaccination, the observed patterns were also consistent when comparing the vaccinated population to the unvaccinated population. When looking at the vaccinated patients immediately before the vaccination compared to an arbitrary date in the past, the mixed effect observed can be attributable to the continuous surveillance of such patients in the CUIMC, which results in having higher health care utilization over an extended time period in the past.

The first implication of these results is that, when comparing vaccinated to unvaccinated patients or time, the anchoring event for the unvaccinated comparator must be selected carefully. Previous research acknowledged that comparing unexposed and exposed patients in the context of vaccine safety and effectiveness surveillance may lead to between-person confounding due to noncomparable groups [15]. For example, as noted before for influenza, vaccinated and unvaccinated patients differ in comorbidity prevalence [16]. Nevertheless, even in the same population, the choice of the index date or event influences both baseline covariates and the incidence rates of conditions following the index date. For COVID-19 vaccination, it appears that the comparison should not be purposely anchored on a health care visit unless it is a relevant vaccination subgroup (eg, those vaccinated at hospital discharge).

Adjusting for confounding will be extremely important, as it appears unlikely that a comparison can be chosen perfectly, although the comparisons between the same participants looking a year prior led to the best equivalency for both influenza and COVID-19 vaccinations. Moreover, the difference in patient characteristics requires a robust selection of covariates for a propensity score model or outcome model as opposed to the current exposed versus unexposed COVID-19 vaccine cohort studies, which only use a limited subset of covariates in their propensity score model [17].

Alternatively, this may argue for a self-controlled study design [18], which mitigates the difference in patient characteristics. However, this design is also sensitive to anchoring (which is what happens on day 0 and around it) and carries other challenges such as accounting for differences in COVID-19 risk over time. For example, we observed that when the time before vaccination is compared to the time before a visit in the past, the former time interval is characterized by higher prevalence of COVID-19 diagnosis and laboratory tests in both data sources, as the previous visits mainly had occurred in 2020 before the COVID-19 pandemic started.

This study has implications beyond using covariates for confounding adjustment. The day 0 results have direct implications for analyses of acute side effects such as anaphylaxis that include day 0 because the side effect often occurs immediately. Any study of such short-term effects must directly account for anchoring to the context in which the vaccination is given. Furthermore, studies that compare effectiveness or safety among vaccines must account for differences in populations and vaccination context. For example, single-dose vaccines may be given preferentially to sicker patients who are unable to return for a second dose, such as those being discharged from the hospital.

### Conclusions

Patient baseline covariates in the unexposed group or time are extremely sensitive to the choice of the index date (anchoring). COVID-19 vaccination and influenza vaccination differ drastically from each other in terms of the populations vaccinated and their status on the day of vaccination. Study designs previously used to assess influenza vaccination must be reassessed for COVID-19 to account for a potentially healthier population and lack of medical activity on the day of vaccination.

## Appendix

Multimedia Appendix 1 Supplementary materials.

## Abbreviations

CUIMC: Columbia University Irving Medical Center health record data set
EHR: electronic health record
Optum EHR: Optum electronic health record data set
