# Supplementary material for: Characterizing Anchoring Bias in Vaccine Comparator Selection Due to Health Care Utilization With COVID-19 and Influenza: Observational Cohort Study
Source: JMIR Public Health Surveill. 2022 Jun 17;8(6):e33099. doi: 10.2196/33099 (PMC9250064; doi:10.2196/33099)
Supplement: Multimedia Appendix 1 [file publichealth_v8i6e33099_app1.doc]

**Multimedia Appendix 1**

**Table S1**. Codes used in the study

| **OMOP concept id** | **Code** | **Name** | **Vocabulary** |
| --- | --- | --- | --- |
| ***COVID-19 vaccines*** | | | |
| 702866 | 212 | SARS-COV-2 (COVID-19) vaccine, vector non-replicating, recombinant spike protein-Ad26, preservative free, 0.5 mL | CVX |
| 724905 | 210 | SARS-COV-2 (COVID-19) vaccine, vector non-replicating, recombinant spike protein-ChAdOx1, preservative free, 0.5 mL | CVX |
| 724906 | 207 | SARS-COV-2 (COVID-19) vaccine, mRNA, spike protein, LNP, preservative free, 100 mcg or 50 mcg dose | CVX |
| 724907 | 208 | SARS-COV-2 (COVID-19) vaccine, mRNA, spike protein, LNP, preservative free, 30 mcg/0.3mL dose | CVX |
| 37003431 | 2468230 | SARS-CoV-2 (COVID-19) vaccine, mRNA-BNT162b2 | RxNorm |
| 37003432 | 2468231 | SARS-CoV-2 (COVID-19) vaccine, mRNA spike protein | RxNorm |
| 37003433 | 2468232 | SARS-CoV-2 (COVID-19) vaccine, mRNA-BNT162b2 0.1 MG/ML | RxNorm |
| 37003434 | 2468233 | SARS-CoV-2 (COVID-19) vaccine, mRNA spike protein Injectable Product | RxNorm |
| 37003435 | 2468234 | SARS-CoV-2 (COVID-19) vaccine, mRNA spike protein Injectable Suspension | RxNorm |
| 37003436 | 2468235 | SARS-CoV-2 (COVID-19) vaccine, mRNA-BNT162b2 0.1 MG/ML Injectable Suspension | RxNorm |
| 37003516 | 2470232 | SARS-CoV-2 (COVID-19) vaccine, mRNA-1273 | RxNorm |
| 37003517 | 2470233 | SARS-CoV-2 (COVID-19) vaccine, mRNA-1273 0.2 MG/ML | RxNorm |
| 37003518 | 2470234 | SARS-CoV-2 (COVID-19) vaccine, mRNA-1273 0.2 MG/ML Injectable Suspension | RxNorm |
| ***Influenza vaccines*** | | | |
| 40213141 | 135 | influenza, high dose seasonal, preservative-free | CVX |
| 40213142 | 153 | Influenza, injectable, Madin Darby Canine Kidney, preservative free | CVX |
| 40213143 | 171 | Influenza, injectable, Madin Darby Canine Kidney, preservative free, quadrivalent | CVX |
| 40213144 | 186 | Influenza, injectable, Madin Darby Canine Kidney, quadrivalent with preservative | CVX |
| 40213146 | 150 | Influenza, injectable, quadrivalent, preservative free | CVX |
| 40213147 | 161 | Influenza, injectable,quadrivalent, preservative free, pediatric | CVX |
| 40213148 | 166 | influenza, intradermal, quadrivalent, preservative free, injectable | CVX |
| 40213149 | 111 | influenza virus vaccine, live, attenuated, for intranasal use | CVX |
| 40213150 | 149 | influenza, live, intranasal, quadrivalent | CVX |
| 40213151 | 155 | Seasonal, trivalent, recombinant, injectable influenza vaccine, preservative free | CVX |
| 40213152 | 185 | Seasonal, quadrivalent, recombinant, injectable influenza vaccine, preservative free | CVX |
| 40213153 | 141 | Influenza, seasonal, injectable | CVX |
| 40213154 | 140 | Influenza, seasonal, injectable, preservative free | CVX |
| 40213155 | 144 | seasonal influenza, intradermal, preservative free | CVX |
| 40213156 | 15 | influenza virus vaccine, split virus (incl. purified surface antigen)-retired CODE | CVX |
| 40213157 | 168 | Seasonal trivalent influenza vaccine, adjuvanted, preservative free | CVX |
| 40213159 | 16 | influenza virus vaccine, whole virus | CVX |
| 40213327 | 151 | influenza nasal, unspecified formulation | CVX |

**Table S2.** Top 50 covariates with the standardized difference of means (SDM) >0.1, covariate proportion and standard deviation, for covariates for comparison of COVID-19 vaccinated patients and unvaccinated patients indexed on (a) a date or (b) a visit, day 0.

| **COVID-19 vaccinated patients (Target) compared to unvaccinated indexed on a date (Comparator)** | | | | **COVID-19 vaccinated patients (Target) compared to unvaccinated indexed on a visit (Comparator)** | | | |
| --- | --- | --- | --- | --- | --- | --- | --- |
| **Covariate name** | **Target, mean (SD)** | **Comparator, mean (SD)** | **SDM** | **Covariate name** | **Target, mean (SD)** | **Comparator, mean (SD)** | **SDM** |
| ***CUIMC EHR*** | | | | | | | |
| Higher proportion in vaccinated patients | | | | Higher proportion in vaccinated patients | | | |
| SARS-CoV-2 (COVID-19) vaccine, mRNA-BNT162b2 0.1 MG/ML Injectable Suspension | 0.69 (0.46) | <0.01 (0) | -2.11 | SARS-CoV-2 (COVID-19) vaccine, mRNA-BNT162b2 0.1 MG/ML Injectable Suspension | 0.71 (0.46) | <0.01 (0) | -2.19 |
| Patient encounter status | 0.46 (0.5) | <0.01 (0.06) | -1.28 | SARS-CoV-2 (COVID-19) vaccine, mRNA-1273 0.2 MG/ML Injectable Suspension | 0.27 (0.44) | <0.01 (0) | -0.86 |
| SARS-CoV-2 (COVID-19) vaccine, mRNA-1273 0.2 MG/ML Injectable Suspension | 0.29 (0.45) | <0.01 (0) | -0.91 | Patient encounter status | 0.46 (0.5) | 0.19 (0.39) | -0.62 |
| SARS-COV-2 (COVID-19) vaccine, vector non-replicating, recombinant spike protein-Ad26, preservative free, 0.5 mL | 0.02 (0.14) | <0.01 (0) | -0.2 | SARS-COV-2 (COVID-19) vaccine, vector non-replicating, recombinant spike protein-Ad26, preservative free, 0.5 mL | 0.03 (0.16) | <0.01 (0) | -0.23 |
| Outpatient visit on day 0 | 0.02 (0.15) | 0.01 (0.1) | -0.11 |  |  |  |  |
| Any visit on day 0 | 0.03 (0.16) | 0.01 (0.11) | -0.11 |  |  |  |  |
| Lower proportion in vaccinated patients | | | | Lower proportion in vaccinated patients | | | |
|  |  |  |  | Any visit on day 0 | 0.03 (0.16) | 1 (0) | 8.63 |
|  |  |  |  | Outpatient visit on day 0 | 0.02 (0.15) | 0.68 (0.47) | 1.88 |
|  |  |  |  | Visit occurrence concept count | 0.04 (0.5) | 1.12 (0.41) | 1.69 |
|  |  |  |  | Systolic blood pressure | 0.02 (0.12) | 0.48 (0.5) | 1.29 |
|  |  |  |  | Diastolic blood pressure | 0.02 (0.12) | 0.48 (0.5) | 1.29 |
|  |  |  |  | Heart rate | 0.01 (0.12) | 0.43 (0.5) | 1.16 |
|  |  |  |  | Tobacco smoking behavior - finding | 0.01 (0.1) | 0.42 (0.49) | 1.15 |
|  |  |  |  | Body weight | 0.01 (0.11) | 0.41 (0.49) | 1.12 |
|  |  |  |  | Body mass index (BMI) [Ratio] | 0.01 (0.11) | 0.4 (0.49) | 1.1 |
|  |  |  |  | Body height | 0.01 (0.09) | 0.33 (0.47) | 0.95 |
|  |  |  |  | Body temperature | 0.01 (0.11) | 0.32 (0.47) | 0.91 |
|  |  |  |  | Oxygen saturation in Arterial blood by Pulse oximetry | 0.01 (0.1) | 0.29 (0.46) | 0.86 |
|  |  |  |  | Respiratory rate | 0.01 (0.09) | 0.21 (0.41) | 0.67 |
|  |  |  |  | A detailed history; A detailed examination; Medical decision making of moderate complexity. Counseling and/o | <0.01 (0.07) | 0.18 (0.38) | 0.63 |
|  |  |  |  | Hemoglobin [Mass/volume] in Blood | 0.01 (0.09) | 0.16 (0.36) | 0.57 |
|  |  |  |  | Hematocrit [Volume Fraction] of Blood by Automated count | 0.01 (0.09) | 0.16 (0.36) | 0.56 |
|  |  |  |  | Erythrocyte distribution width [Ratio] by Automated count | 0.01 (0.09) | 0.15 (0.36) | 0.56 |
|  |  |  |  | Leukocytes [#/volume] in Blood by Automated count | 0.01 (0.09) | 0.16 (0.36) | 0.56 |
|  |  |  |  | Platelets [#/volume] in Blood by Automated count | 0.01 (0.09) | 0.15 (0.36) | 0.56 |
|  |  |  |  | MCV [Entitic volume] by Automated count | 0.01 (0.09) | 0.15 (0.36) | 0.56 |
|  |  |  |  | MCHC [Mass/volume] by Automated count | 0.01 (0.09) | 0.15 (0.36) | 0.56 |
|  |  |  |  | MCH [Entitic mass] by Automated count | 0.01 (0.09) | 0.15 (0.36) | 0.56 |
|  |  |  |  | Erythrocytes [#/volume] in Blood by Automated count | 0.01 (0.09) | 0.15 (0.36) | 0.56 |
|  |  |  |  | Creatinine [Mass/volume] in Serum or Plasma | 0.01 (0.09) | 0.15 (0.36) | 0.55 |
|  |  |  |  | Calcium [Mass/volume] in Serum or Plasma | 0.01 (0.09) | 0.15 (0.36) | 0.55 |
|  |  |  |  | Urea nitrogen [Mass/volume] in Serum or Plasma | 0.01 (0.09) | 0.15 (0.36) | 0.55 |
|  |  |  |  | Chloride [Moles/volume] in Serum or Plasma | 0.01 (0.09) | 0.15 (0.36) | 0.55 |
|  |  |  |  | Sodium [Moles/volume] in Serum or Plasma | 0.01 (0.09) | 0.15 (0.36) | 0.55 |
|  |  |  |  | Potassium [Moles/volume] in Serum or Plasma | 0.01 (0.09) | 0.15 (0.36) | 0.55 |
|  |  |  |  | Carbon dioxide, total [Moles/volume] in Serum or Plasma | 0.01 (0.09) | 0.15 (0.36) | 0.55 |
|  |  |  |  | Platelet mean volume [Entitic volume] in Blood by Automated count | 0.01 (0.09) | 0.15 (0.35) | 0.54 |
|  |  |  |  | Nucleated erythrocytes/100 leukocytes [Ratio] in Blood by Automated count | 0.01 (0.08) | 0.14 (0.35) | 0.53 |
|  |  |  |  | Nucleated erythrocytes [#/volume] in Blood by Automated count | 0.01 (0.08) | 0.14 (0.35) | 0.53 |
|  |  |  |  | Anion gap in Serum or Plasma | 0.01 (0.09) | 0.14 (0.35) | 0.53 |
|  |  |  |  | Glomerular filtration rate/1.73 sq M.predicted [Volume Rate/Area] in Serum, Plasma or Blood by Creatinine-based formula (MDRD) | 0.01 (0.09) | 0.14 (0.35) | 0.53 |
|  |  |  |  | Electrocardiogram, routine ECG with at least 12 leads; with interpretation and report | <0.01 (0.04) | 0.13 (0.33) | 0.52 |
|  |  |  |  | Emergency room visit on day 0 | <0.01 (0.03) | 0.12 (0.33) | 0.52 |
|  |  |  |  | An expanded problem focused history; An expanded problem focused examination; Medical decision making of low | <0.01 (0.06) | 0.12 (0.33) | 0.5 |
|  |  |  |  | Essential hypertension | <0.01 (0.06) | 0.12 (0.33) | 0.5 |
|  |  |  |  | Alanine aminotransferase [Enzymatic activity/volume] in Serum or Plasma | 0.01 (0.08) | 0.13 (0.33) | 0.49 |
|  |  |  |  | Aspartate aminotransferase [Enzymatic activity/volume] in Serum or Plasma | 0.01 (0.08) | 0.12 (0.33) | 0.49 |
|  |  |  |  | Protein [Mass/volume] in Serum or Plasma | 0.01 (0.08) | 0.12 (0.33) | 0.49 |
|  |  |  |  | Albumin [Mass/volume] in Serum or Plasma | 0.01 (0.08) | 0.12 (0.33) | 0.49 |
|  |  |  |  | Bilirubin.total [Mass/volume] in Serum or Plasma | 0.01 (0.08) | 0.12 (0.33) | 0.49 |
|  |  |  |  | Alkaline phosphatase [Enzymatic activity/volume] in Serum or Plasma | 0.01 (0.08) | 0.12 (0.33) | 0.49 |
|  |  |  |  | Neutrophils [#/volume] in Blood by Automated count | <0.01 (0.07) | 0.12 (0.32) | 0.49 |
|  |  |  |  | Basophils [#/volume] in Blood by Automated count | <0.01 (0.07) | 0.12 (0.32) | 0.49 |
|  |  |  |  | Eosinophils [#/volume] in Blood by Automated count | <0.01 (0.07) | 0.12 (0.32) | 0.49 |
|  |  |  |  | Monocytes [#/volume] in Blood by Automated count | <0.01 (0.07) | 0.12 (0.32) | 0.49 |
|  |  |  |  | Lymphocytes [#/volume] in Blood by Automated count | <0.01 (0.07) | 0.12 (0.32) | 0.49 |
| ***Optum EHR*** | | | | | | | |
| Higher proportion in vaccinated patients | | | | Higher proportion in vaccinated patients | | | |
| Visit occurrence concept count | 0.73 (0.05) | 0.15 (0.23) | -2.46 | SARS-CoV-2 (COVID-19) vaccine, mRNA-BNT162b2 0.1 MG/ML Injectable Suspension | 0.95 (0.98) | <0.01 (0.01) | -0.98 |
| SARS-CoV-2 (COVID-19) vaccine, mRNA-BNT162b2 0.1 MG/ML Injectable Suspension | 0.95 (0.98) | <0.01 (0.01) | -0.98 | SARS-CoV-2 (COVID-19) vaccine, mRNA-1273 0.2 MG/ML Injectable Suspension | 0.33 (0.58) | <0.01 (0) | -0.58 |
| Patient encounter procedure | 0.61 (0.78) | 0.02 (0.16) | -0.73 | Patient encounter procedure | 0.61 (0.78) | 0.12 (0.35) | -0.57 |
| Outpatient visit on day 0 | 0.72 (0.85) | 0.13 (0.36) | -0.64 | Requires vaccination | 0.17 (0.41) | <0.01 (0.05) | -0.4 |
| Any visit on day 0 | 0.73 (0.85) | 0.15 (0.38) | -0.62 | Vaccine Administration | 0.14 (0.37) | <0.01 (0.04) | -0.36 |
| SARS-CoV-2 (COVID-19) vaccine, mRNA-1273 0.2 MG/ML Injectable Suspension | 0.33 (0.58) | <0.01 (0) | -0.58 | Drugs Identification - Drugs Requiring Detailed Coding | 0.09 (0.3) | 0.02 (0.14) | -0.21 |
| Requires vaccination | 0.17 (0.41) | <0.01 (0.03) | -0.41 | SARS-COV-2 (COVID-19) vaccine, UNSPECIFIED | 0.03 (0.17) | <0.01 (0.04) | -0.16 |
| Vaccine Administration | 0.14 (0.37) | <0.01 (0.01) | -0.37 | Pharmacy - General Classification | 0.05 (0.22) | 0.02 (0.13) | -0.13 |
| Drugs Identification - Drugs Requiring Detailed Coding | 0.09 (0.3) | 0.01 (0.09) | -0.26 |  |  |  |  |
| SARS-COV-2 (COVID-19) vaccine, UNSPECIFIED | 0.03 (0.17) | <0.01 (0.02) | -0.17 |  |  |  |  |
| Pharmacy - General Classification | 0.05 (0.22) | 0.01 (0.09) | -0.17 |  |  |  |  |
| Lower proportion in vaccinated patients | | | | Lower proportion in vaccinated patients | | | |
|  |  |  |  | Visit occurrence concept count | 0.73 (0.05) | 1.04 (0.2) | 1.52 |
|  |  |  |  | Body weight | 0.02 (0.14) | 0.25 (0.5) | 0.44 |
|  |  |  |  | Systolic blood pressure | 0.02 (0.15) | 0.25 (0.5) | 0.43 |
|  |  |  |  | Diastolic blood pressure | 0.02 (0.15) | 0.24 (0.49) | 0.42 |
|  |  |  |  | Pulse intensity of Unspecified artery palpation | 0.02 (0.15) | 0.24 (0.49) | 0.42 |
|  |  |  |  | Body height | 0.02 (0.13) | 0.22 (0.47) | 0.42 |
|  |  |  |  | Body mass index (BMI) [Ratio] | 0.02 (0.14) | 0.22 (0.47) | 0.41 |
|  |  |  |  | Smoking status [FTND] | 0.02 (0.14) | 0.2 (0.45) | 0.39 |
|  |  |  |  | Body temperature | 0.02 (0.16) | 0.21 (0.46) | 0.39 |
|  |  |  |  | Temperature | 0.02 (0.16) | 0.21 (0.46) | 0.39 |
|  |  |  |  | Oxygen [Partial pressure] in Blood | 0.02 (0.13) | 0.17 (0.42) | 0.36 |
|  |  |  |  | Respiratory rate | 0.01 (0.11) | 0.15 (0.39) | 0.35 |
|  |  |  |  | Alcohol intake | 0.01 (0.12) | 0.14 (0.37) | 0.32 |
|  |  |  |  | Glucose [Mass/volume] in Serum or Plasma | 0.01 (0.1) | 0.09 (0.31) | 0.26 |
|  |  |  |  | Leukocytes [#/volume] in Blood | 0.01 (0.1) | 0.09 (0.3) | 0.25 |
|  |  |  |  | Erythrocytes [#/volume] in Blood | 0.01 (0.1) | 0.09 (0.3) | 0.25 |
|  |  |  |  | Cotinine/Creatinine [Mass Ratio] in Urine | 0.01 (0.1) | 0.09 (0.3) | 0.25 |
|  |  |  |  | Penicillin G potassium [Mass] of Dose | 0.01 (0.1) | 0.09 (0.3) | 0.25 |
|  |  |  |  | Sodium [Moles/volume] in Saliva (oral fluid) | 0.01 (0.1) | 0.09 (0.3) | 0.25 |
|  |  |  |  | Chloride [Moles/volume] in Saliva (oral fluid) | 0.01 (0.1) | 0.09 (0.3) | 0.25 |
|  |  |  |  | Hemoglobin [Mass/volume] in Blood | 0.01 (0.1) | 0.09 (0.29) | 0.25 |
|  |  |  |  | Hematocrit [Volume Fraction] of Blood | 0.01 (0.1) | 0.09 (0.29) | 0.25 |
|  |  |  |  | Calcium.ionized/Calcium.total corrected for albumin in Blood | 0.01 (0.1) | 0.09 (0.3) | 0.25 |
|  |  |  |  | Platelets [#/volume] in Blood | 0.01 (0.09) | 0.08 (0.29) | 0.25 |
|  |  |  |  | MCHC [Mass/volume] | 0.01 (0.09) | 0.08 (0.29) | 0.25 |
|  |  |  |  | MCV [Entitic volume] | 0.01 (0.09) | 0.08 (0.29) | 0.25 |
|  |  |  |  | MCH [Entitic mass] | 0.01 (0.09) | 0.08 (0.29) | 0.25 |
|  |  |  |  | Platelet distribution width [Ratio] in Blood | 0.01 (0.09) | 0.08 (0.29) | 0.24 |
|  |  |  |  | Anion gap in Blood | 0.01 (0.09) | 0.08 (0.28) | 0.24 |
|  |  |  |  | Carbon dioxide [Partial pressure] in Blood | 0.01 (0.1) | 0.08 (0.29) | 0.24 |
|  |  |  |  | Pain severity - 0-10 verbal numeric rating [Score] - Reported | <0.01 (0.07) | 0.07 (0.26) | 0.23 |
|  |  |  |  | Emergency room visit on day 0 | <0.01 (0.03) | 0.06 (0.24) | 0.23 |
|  |  |  |  | Essential hypertension | 0.01 (0.09) | 0.07 (0.27) | 0.23 |
|  |  |  |  | Neutrophil cytoplasmic Ab.perinuclear [Presence] in Serum | 0.01 (0.09) | 0.07 (0.27) | 0.23 |
|  |  |  |  | Lymphocytes/100 leukocytes in Blood | 0.01 (0.09) | 0.07 (0.27) | 0.23 |
|  |  |  |  | Neutrophil Ab [Presence] in Serum | 0.01 (0.09) | 0.07 (0.27) | 0.23 |
|  |  |  |  | Monocytes/100 leukocytes in Blood | 0.01 (0.09) | 0.07 (0.26) | 0.23 |
|  |  |  |  | An expanded problem focused history; An expanded problem focused examination; Medical decision making of low | 0.01 (0.07) | 0.06 (0.25) | 0.22 |
|  |  |  |  | Basophils/100 leukocytes in Blood | 0.01 (0.09) | 0.07 (0.26) | 0.22 |
|  |  |  |  | Eosinophils/100 leukocytes in Blood | 0.01 (0.09) | 0.07 (0.26) | 0.22 |
|  |  |  |  | SARS-CoV-2 (COVID-19) RNA [Presence] in Respiratory specimen by NAA with probe detection | <0.01 (0.07) | 0.06 (0.24) | 0.22 |
|  |  |  |  | A detailed history; A detailed examination; Medical decision making of moderate complexity. Counseling and/o | 0.01 (0.09) | 0.06 (0.25) | 0.21 |
|  |  |  |  | Alanine aminotransferase [Enzymatic activity/volume] in Serum or Plasma | 0.01 (0.09) | 0.06 (0.25) | 0.21 |
|  |  |  |  | Aspartate aminotransferase [Presence] in Body fluid | 0.01 (0.09) | 0.06 (0.25) | 0.21 |
|  |  |  |  | Bilirubin.total [Presence] in Urine | 0.01 (0.08) | 0.06 (0.25) | 0.21 |
|  |  |  |  | Alkaline phosphatase.renal/Alkaline phosphatase.total in Serum or Plasma | 0.01 (0.08) | 0.06 (0.25) | 0.21 |
|  |  |  |  | Albumin [Presence] in Urine | 0.01 (0.09) | 0.06 (0.25) | 0.21 |
|  |  |  |  | Any visit on day 0 | 0.73 (0.85) | 1 (1) | 0.21 |
|  |  |  |  | Albumin/Protein.total in Serum or Plasma | 0.01 (0.09) | 0.06 (0.25) | 0.21 |
|  |  |  |  | Platelet mean volume [Entitic volume] in Blood | 0.01 (0.08) | 0.06 (0.24) | 0.2 |

**Table S3.** Top 50 covariates with the standardized difference of means (SDM) >0.1, covariate proportion and standard deviation, for comparison of influenza vaccinated patients and unvaccinated patients indexed on (a) a date or (b) a visit, day 0.

| **Influenza vaccinated patients (Target) compared to unvaccinated indexed on a date (Comparator)** | | | | **Influenza vaccinated patients (Target) compared to unvaccinated indexed on a visit (Comparator)** | | | |
| --- | --- | --- | --- | --- | --- | --- | --- |
| **Covariate name** | **Target, mean (SD)** | **Comparator, mean (SD)** | **SDM** | **Covariate name** | **Target, mean (SD)** | **Comparator, mean (SD)** | **SDM** |
| ***CUIMC EHR*** | | | | | | | |
| Higher proportion in vaccinated patients | | | | Higher proportion in vaccinated patients | | | |
| Systolic blood pressure | 0.67 (0.47) | 0.01 (0.09) | -1.97 | Influenza, injectable, quadrivalent, preservative free | 0.35 (0.48) | <0.01 (0.02) | -1.03 |
| Diastolic blood pressure | 0.67 (0.47) | 0.01 (0.09) | -1.97 | 0.5 ML influenza A virus A/Hong Kong/4801/2014 (H3N2) antigen 0.03 MG/ML / influenza A virus A/Michigan/45/2015 (H1N1) antigen 0.03 MG/ML / influenza B virus B/Brisbane/60/2008 antigen 0.03 MG/ML / influenza B virus B/Phuket/3073/2013 antigen 0.03 MG/M... | 0.27 (0.44) | <0.01 (0) | -0.86 |
| Body weight | 0.62 (0.48) | 0.01 (0.07) | -1.79 | Immunization administration (includes percutaneous, intradermal, subcutaneous, or intramuscular injections); 1 vaccine (single or combination vaccine/toxoid) | 0.18 (0.39) | 0.01 (0.08) | -0.63 |
| Heart rate | 0.6 (0.49) | 0.01 (0.08) | -1.7 | Patient encounter status | 0.15 (0.35) | <0.01 (0.02) | -0.58 |
| Body mass index (BMI) [Ratio] | 0.56 (0.5) | <0.01 (0.07) | -1.58 | Vaccination needed | 0.14 (0.34) | <0.01 (0) | -0.57 |
| Any visit on day 0 | 0.55 (0.5) | 0.01 (0.07) | -1.53 | Tobacco smoking behavior - finding | 0.44 (0.5) | 0.2 (0.4) | -0.55 |
| Outpatient visit on day 0 | 0.55 (0.5) | <0.01 (0.07) | -1.53 | Patient encounter procedure | 0.3 (0.46) | 0.09 (0.29) | -0.54 |
| Tobacco smoking behavior - finding | 0.49 (0.5) | 0.01 (0.07) | -1.36 | influenza virus vaccine, unspecified formulation | 0.13 (0.33) | <0.01 (0.02) | -0.54 |
| Body temperature | 0.36 (0.48) | 0.01 (0.07) | -1.04 | Body height Measured | 0.29 (0.45) | 0.09 (0.29) | -0.52 |
| 0.5 ML influenza A virus A/Hong Kong/4801/2014 (H3N2) antigen 0.03 MG/ML / influenza A virus A/Michigan/45/2015 (H1N1) antigen 0.03 MG/ML / influenza B virus B/Brisbane/60/2008 antigen 0.03 MG/ML / influenza B virus B/Phuket/3073/2013 antigen 0.03 MG/M... | 0.32 (0.47) | 0.01 (0.01) | -0.96 | 0.5 ML influenza A virus A/Hong Kong/4801/2014 (H3N2) antigen 0.03 MG/ML / influenza A virus A/Singapore/GP1908/2015 (H1N1) antigen 0.03 MG/ML / influenza B virus B/Brisbane/60/2008 antigen 0.03 MG/ML / influenza B virus B/Phuket/3073/2013 antigen 0.03... | 0.12 (0.32) | <0.01 (0) | -0.52 |
| Patient encounter procedure | 0.29 (0.46) | <0.01 (0.02) | -0.91 | Body weight | 0.61 (0.49) | 0.37 (0.48) | -0.49 |
| Body height Measured | 0.29 (0.45) | <0.01 (0.02) | -0.89 | Needs influenza immunization | 0.1 (0.3) | <0.01 (0) | -0.46 |
| Influenza, injectable, quadrivalent, preservative free | 0.26 (0.44) | <0.01 (0.01) | -0.84 | Follow-up encounter | 0.13 (0.34) | 0.01 (0.12) | -0.45 |
| Hemoglobin [Mass/volume] in Blood | 0.27 (0.44) | <0.01 (0.07) | -0.83 | Body mass index (BMI) [Ratio] | 0.56 (0.5) | 0.34 (0.47) | -0.45 |
| Hematocrit [Volume Fraction] of Blood by Automated count | 0.26 (0.44) | <0.01 (0.07) | -0.82 | Cholesterol [Mass/volume] in Serum or Plasma | 0.14 (0.34) | 0.03 (0.16) | -0.42 |
| Leukocytes [#/volume] in Blood by Automated count | 0.26 (0.44) | <0.01 (0.07) | -0.82 | Cholesterol in HDL [Mass/volume] in Serum or Plasma | 0.13 (0.34) | 0.02 (0.16) | -0.41 |
| MCHC [Mass/volume] by Automated count | 0.26 (0.44) | <0.01 (0.07) | -0.82 | Cholesterol in LDL [Mass/volume] in Serum or Plasma by calculation | 0.13 (0.34) | 0.02 (0.15) | -0.41 |
| MCH [Entitic mass] by Automated count | 0.26 (0.44) | <0.01 (0.07) | -0.82 | Triglyceride [Mass/volume] in Serum or Plasma | 0.13 (0.34) | 0.02 (0.16) | -0.41 |
| Erythrocyte distribution width [Ratio] by Automated count | 0.26 (0.44) | <0.01 (0.07) | -0.82 | Cholesterol.total/Cholesterol in HDL [Mass Ratio] in Serum or Plasma | 0.13 (0.34) | 0.02 (0.15) | -0.4 |
| Erythrocytes [#/volume] in Blood by Automated count | 0.26 (0.44) | <0.01 (0.07) | -0.82 | 0.5 ML influenza A virus A/Hong Kong/4801/2014 (H3N2) antigen 0.12 MG/ML / influenza A virus A/Michigan/45/2015 (H1N1) antigen 0.12 MG/ML / influenza B virus B/Brisbane/60/2008 antigen 0.12 MG/ML Prefilled Syringe [Fluzone 2017-2018] | 0.07 (0.26) | <0.01 (0) | -0.4 |
| MCV [Entitic volume] by Automated count | 0.26 (0.44) | <0.01 (0.07) | -0.82 | influenza, high dose seasonal, preservative-free | 0.06 (0.24) | <0.01 (0) | -0.37 |
| Platelets [#/volume] in Blood by Automated count | 0.26 (0.44) | <0.01 (0.07) | -0.81 | Administration of influenza vaccine | 0.07 (0.25) | <0.01 (0.05) | -0.36 |
| Creatinine [Mass/volume] in Serum or Plasma | 0.26 (0.44) | <0.01 (0.07) | -0.81 | Immunization administration through 18 years of age via any route of administration, with counseling by physician or other qualified health care professional; first or only component of each vaccine or toxoid administered | 0.06 (0.25) | <0.01 (0.04) | -0.36 |
| Urea nitrogen [Mass/volume] in Serum or Plasma | 0.26 (0.44) | <0.01 (0.07) | -0.81 | Immunization status | 0.06 (0.23) | <0.01 (0) | -0.34 |
| Sodium [Moles/volume] in Serum or Plasma | 0.26 (0.44) | <0.01 (0.07) | -0.81 | Body height [Percentile] | 0.14 (0.35) | 0.04 (0.2) | -0.34 |
| Chloride [Moles/volume] in Serum or Plasma | 0.26 (0.44) | <0.01 (0.07) | -0.81 | Urea nitrogen/Creatinine [Mass Ratio] in Serum or Plasma | 0.09 (0.29) | 0.02 (0.13) | -0.34 |
| Potassium [Moles/volume] in Serum or Plasma | 0.26 (0.44) | <0.01 (0.07) | -0.81 | Platelets [#/volume] in Blood by Automated count | 0.22 (0.41) | 0.1 (0.3) | -0.33 |
| Calcium [Mass/volume] in Serum or Plasma | 0.26 (0.44) | <0.01 (0.07) | -0.81 | Hematocrit [Volume Fraction] of Blood by Automated count | 0.22 (0.41) | 0.1 (0.3) | -0.33 |
| Carbon dioxide, total [Moles/volume] in Serum or Plasma | 0.26 (0.44) | <0.01 (0.07) | -0.81 | Leukocytes [#/volume] in Blood by Automated count | 0.22 (0.41) | 0.1 (0.3) | -0.33 |
| Platelet mean volume [Entitic volume] in Blood by Automated count | 0.24 (0.43) | <0.01 (0.06) | -0.77 | Erythrocytes [#/volume] in Blood by Automated count | 0.22 (0.41) | 0.1 (0.3) | -0.33 |
| Anion gap in Serum or Plasma | 0.24 (0.43) | <0.01 (0.07) | -0.77 | MCHC [Mass/volume] by Automated count | 0.22 (0.41) | 0.1 (0.3) | -0.33 |
| Glomerular filtration rate/1.73 sq M.predicted [Volume Rate/Area] in Serum, Plasma or Blood by Creatinine-based formula (MDRD) | 0.24 (0.42) | <0.01 (0.06) | -0.76 | MCH [Entitic mass] by Automated count | 0.22 (0.41) | 0.1 (0.3) | -0.33 |
| Visit occurrence concept count | 0.74 (0.76) | 0.01 (0.61) | -0.75 | MCV [Entitic volume] by Automated count | 0.22 (0.41) | 0.1 (0.3) | -0.33 |
| Alanine aminotransferase [Enzymatic activity/volume] in Serum or Plasma | 0.21 (0.41) | <0.01 (0.06) | -0.71 | Erythrocyte distribution width [Ratio] by Automated count | 0.22 (0.41) | 0.1 (0.3) | -0.33 |
| Protein [Mass/volume] in Serum or Plasma | 0.21 (0.4) | <0.01 (0.06) | -0.7 | Hemoglobin [Mass/volume] in Blood | 0.23 (0.42) | 0.11 (0.31) | -0.32 |
| Aspartate aminotransferase [Enzymatic activity/volume] in Serum or Plasma | 0.21 (0.4) | <0.01 (0.06) | -0.7 | Hemoglobin A1c/Hemoglobin.total in Blood | 0.11 (0.31) | 0.03 (0.17) | -0.32 |
| Bilirubin.total [Mass/volume] in Serum or Plasma | 0.2 (0.4) | <0.01 (0.06) | -0.7 | Requires vaccination | 0.04 (0.2) | <0.01 (0) | -0.3 |
| Albumin [Mass/volume] in Serum or Plasma | 0.2 (0.4) | <0.01 (0.06) | -0.7 | Glomerular filtration rate/1.73 sq M.predicted [Volume Rate/Area] in Serum, Plasma or Blood by Creatinine-based formula (MDRD) | 0.17 (0.37) | 0.07 (0.26) | -0.29 |
| Alkaline phosphatase [Enzymatic activity/volume] in Serum or Plasma | 0.2 (0.4) | <0.01 (0.06) | -0.7 | Platelet mean volume [Entitic volume] in Blood by Automated count | 0.2 (0.4) | 0.1 (0.3) | -0.29 |
| Body height | 0.2 (0.4) | <0.01 (0.06) | -0.69 | Influenza, seasonal, injectable | 0.04 (0.19) | <0.01 (0) | -0.29 |
| Respiratory rate | 0.2 (0.4) | <0.01 (0.07) | -0.68 | Urea nitrogen [Mass/volume] in Serum or Plasma | 0.19 (0.39) | 0.09 (0.29) | -0.28 |
| Immunization administration (includes percutaneous, intradermal, subcutaneous, or intramuscular injections); 1 vaccine (single or combination vaccine/toxoid) | 0.18 (0.39) | <0.01 (0.02) | -0.67 | Creatinine [Mass/volume] in Serum or Plasma | 0.19 (0.39) | 0.09 (0.29) | -0.28 |
| Globulin [Mass/volume] in Serum | 0.19 (0.39) | <0.01 (0.06) | -0.66 | Potassium [Moles/volume] in Serum or Plasma | 0.19 (0.39) | 0.09 (0.29) | -0.28 |
| Cholesterol [Mass/volume] in Serum or Plasma | 0.18 (0.38) | <0.01 (0.03) | -0.65 | Sodium [Moles/volume] in Serum or Plasma | 0.19 (0.39) | 0.09 (0.29) | -0.28 |
| Cholesterol in HDL [Mass/volume] in Serum or Plasma | 0.17 (0.38) | <0.01 (0.03) | -0.64 | Chloride [Moles/volume] in Serum or Plasma | 0.19 (0.39) | 0.09 (0.29) | -0.28 |
| Triglyceride [Mass/volume] in Serum or Plasma | 0.17 (0.38) | <0.01 (0.03) | -0.64 | Calcium [Mass/volume] in Serum or Plasma | 0.19 (0.39) | 0.09 (0.29) | -0.28 |
| Cholesterol in LDL [Mass/volume] in Serum or Plasma by calculation | 0.17 (0.38) | <0.01 (0.03) | -0.64 | Carbon dioxide, total [Moles/volume] in Serum or Plasma | 0.19 (0.39) | 0.09 (0.29) | -0.28 |
| Vaccination needed | 0.17 (0.37) | <0.01 (0.01) | -0.63 | Alanine aminotransferase [Enzymatic activity/volume] in Serum or Plasma | 0.16 (0.37) | 0.07 (0.26) | -0.27 |
| influenza virus vaccine, unspecified formulation | 0.16 (0.37) | <0.01 (0) | -0.62 | Aspartate aminotransferase [Enzymatic activity/volume] in Serum or Plasma | 0.15 (0.36) | 0.07 (0.26) | -0.26 |
| Essential hypertension | 0.16 (0.37) | <0.01 (0.04) | -0.61 | Protein [Mass/volume] in Serum or Plasma | 0.15 (0.36) | 0.07 (0.26) | -0.26 |
| Lower proportion in vaccinated patients | | | | Lower proportion in vaccinated patients | | | |
|  |  |  |  | Any visit on day 0 | 0.03 (0.16) | 1 (0) | 8.63 |
|  |  |  |  | Outpatient visit on day 0 | 0.02 (0.15) | 0.68 (0.47) | 1.88 |
|  |  |  |  | Visit occurrence concept count | 0.04 (0.5) | 1.12 (0.41) | 1.69 |
|  |  |  |  | Systolic blood pressure | 0.02 (0.12) | 0.48 (0.5) | 1.29 |
|  |  |  |  | Diastolic blood pressure | 0.02 (0.12) | 0.48 (0.5) | 1.29 |
|  |  |  |  | Heart rate | 0.01 (0.12) | 0.43 (0.5) | 1.16 |
|  |  |  |  | Tobacco smoking behavior - finding | 0.01 (0.1) | 0.42 (0.49) | 1.15 |
|  |  |  |  | Body weight | 0.01 (0.11) | 0.41 (0.49) | 1.12 |
|  |  |  |  | Body mass index (BMI) [Ratio] | 0.01 (0.11) | 0.4 (0.49) | 1.1 |
|  |  |  |  | Body height | 0.01 (0.09) | 0.33 (0.47) | 0.95 |
|  |  |  |  | Body temperature | 0.01 (0.11) | 0.32 (0.47) | 0.91 |
|  |  |  |  | Oxygen saturation in Arterial blood by Pulse oximetry | 0.01 (0.1) | 0.29 (0.46) | 0.86 |
|  |  |  |  | Respiratory rate | 0.01 (0.09) | 0.21 (0.41) | 0.67 |
|  |  |  |  | A detailed history; A detailed examination; Medical decision making of moderate complexity. Counseling and/o | <0.01 (0.07) | 0.18 (0.38) | 0.63 |
|  |  |  |  | Hemoglobin [Mass/volume] in Blood | 0.01 (0.09) | 0.16 (0.36) | 0.57 |
|  |  |  |  | Hematocrit [Volume Fraction] of Blood by Automated count | 0.01 (0.09) | 0.16 (0.36) | 0.56 |
|  |  |  |  | Erythrocyte distribution width [Ratio] by Automated count | 0.01 (0.09) | 0.15 (0.36) | 0.56 |
|  |  |  |  | Leukocytes [#/volume] in Blood by Automated count | 0.01 (0.09) | 0.16 (0.36) | 0.56 |
|  |  |  |  | Platelets [#/volume] in Blood by Automated count | 0.01 (0.09) | 0.15 (0.36) | 0.56 |
|  |  |  |  | MCV [Entitic volume] by Automated count | 0.01 (0.09) | 0.15 (0.36) | 0.56 |
|  |  |  |  | MCHC [Mass/volume] by Automated count | 0.01 (0.09) | 0.15 (0.36) | 0.56 |
|  |  |  |  | MCH [Entitic mass] by Automated count | 0.01 (0.09) | 0.15 (0.36) | 0.56 |
|  |  |  |  | Erythrocytes [#/volume] in Blood by Automated count | 0.01 (0.09) | 0.15 (0.36) | 0.56 |
|  |  |  |  | Creatinine [Mass/volume] in Serum or Plasma | 0.01 (0.09) | 0.15 (0.36) | 0.55 |
|  |  |  |  | Calcium [Mass/volume] in Serum or Plasma | 0.01 (0.09) | 0.15 (0.36) | 0.55 |
|  |  |  |  | Urea nitrogen [Mass/volume] in Serum or Plasma | 0.01 (0.09) | 0.15 (0.36) | 0.55 |
|  |  |  |  | Chloride [Moles/volume] in Serum or Plasma | 0.01 (0.09) | 0.15 (0.36) | 0.55 |
|  |  |  |  | Sodium [Moles/volume] in Serum or Plasma | 0.01 (0.09) | 0.15 (0.36) | 0.55 |
|  |  |  |  | Potassium [Moles/volume] in Serum or Plasma | 0.01 (0.09) | 0.15 (0.36) | 0.55 |
|  |  |  |  | Carbon dioxide, total [Moles/volume] in Serum or Plasma | 0.01 (0.09) | 0.15 (0.36) | 0.55 |
|  |  |  |  | Platelet mean volume [Entitic volume] in Blood by Automated count | 0.01 (0.09) | 0.15 (0.35) | 0.54 |
|  |  |  |  | Nucleated erythrocytes/100 leukocytes [Ratio] in Blood by Automated count | 0.01 (0.08) | 0.14 (0.35) | 0.53 |
|  |  |  |  | Nucleated erythrocytes [#/volume] in Blood by Automated count | 0.01 (0.08) | 0.14 (0.35) | 0.53 |
|  |  |  |  | Anion gap in Serum or Plasma | 0.01 (0.09) | 0.14 (0.35) | 0.53 |
|  |  |  |  | Glomerular filtration rate/1.73 sq M.predicted [Volume Rate/Area] in Serum, Plasma or Blood by Creatinine-based formula (MDRD) | 0.01 (0.09) | 0.14 (0.35) | 0.53 |
|  |  |  |  | Electrocardiogram, routine ECG with at least 12 leads; with interpretation and report | <0.01 (0.04) | 0.13 (0.33) | 0.52 |
|  |  |  |  | Emergency room visit on day 0 | <0.01 (0.03) | 0.12 (0.33) | 0.52 |
|  |  |  |  | An expanded problem focused history; An expanded problem focused examination; Medical decision making of low | <0.01 (0.06) | 0.12 (0.33) | 0.5 |
|  |  |  |  | Essential hypertension | <0.01 (0.06) | 0.12 (0.33) | 0.5 |
|  |  |  |  | Alanine aminotransferase [Enzymatic activity/volume] in Serum or Plasma | 0.01 (0.08) | 0.13 (0.33) | 0.49 |
|  |  |  |  | Aspartate aminotransferase [Enzymatic activity/volume] in Serum or Plasma | 0.01 (0.08) | 0.12 (0.33) | 0.49 |
|  |  |  |  | Protein [Mass/volume] in Serum or Plasma | 0.01 (0.08) | 0.12 (0.33) | 0.49 |
|  |  |  |  | Albumin [Mass/volume] in Serum or Plasma | 0.01 (0.08) | 0.12 (0.33) | 0.49 |
|  |  |  |  | Bilirubin.total [Mass/volume] in Serum or Plasma | 0.01 (0.08) | 0.12 (0.33) | 0.49 |
|  |  |  |  | Alkaline phosphatase [Enzymatic activity/volume] in Serum or Plasma | 0.01 (0.08) | 0.12 (0.33) | 0.49 |
|  |  |  |  | Neutrophils [#/volume] in Blood by Automated count | <0.01 (0.07) | 0.12 (0.32) | 0.49 |
|  |  |  |  | Basophils [#/volume] in Blood by Automated count | <0.01 (0.07) | 0.12 (0.32) | 0.49 |
|  |  |  |  | Eosinophils [#/volume] in Blood by Automated count | <0.01 (0.07) | 0.12 (0.32) | 0.49 |
|  |  |  |  | Monocytes [#/volume] in Blood by Automated count | <0.01 (0.07) | 0.12 (0.32) | 0.49 |
|  |  |  |  | Lymphocytes [#/volume] in Blood by Automated count | <0.01 (0.07) | 0.12 (0.32) | 0.49 |
| ***Optum EHR*** | | | | | | | |
| Higher proportion in vaccinated patients | | | | Higher proportion in vaccinated patients | | | |
| visit_occurrence concept count | 0.56 (0.24) | 0.03 (0.21) | -1.67 | 0.5 ML influenza A virus A/North Carolina/04/2016 (H3N2) antigen 0.03 MG/ML / influenza A virus A/Singapore/GP1908/2015 (H1N1) antigen 0.03 MG/ML / influenza B virus B/Iowa/06/2017 antigen 0.03 MG/ML / influenza B virus B/Singapore/INFTT-16-0610/2016 a... | 0.95 (0.97) | <0.01 (0.02) | -0.97 |
| 0.5 ML influenza A virus A/North Carolina/04/2016 (H3N2) antigen 0.03 MG/ML / influenza A virus A/Singapore/GP1908/2015 (H1N1) antigen 0.03 MG/ML / influenza B virus B/Iowa/06/2017 antigen 0.03 MG/M... | 0.95 (0.97) | <0.01 (0) | -0.97 | Immunization administration (includes percutaneous, intradermal, subcutaneous, or intramuscular injections); 1 vaccine (single or combination vaccine/toxoid) | 0.26 (0.51) | 0.01 (0.09) | -0.49 |
| Any visit on day 0 | 0.53 (0.73) | 0.03 (0.16) | -0.68 | Influenza, injectable, quadrivalent, preservative free | 0.23 (0.48) | <0.01 (0.01) | -0.48 |
| Patient encounter procedure | 0.46 (0.68) | <0.01 (0.05) | -0.67 | Patient encounter procedure | 0.46 (0.68) | 0.13 (0.36) | -0.44 |
| Outpatient visit on day 0 | 0.51 (0.72) | 0.02 (0.15) | -0.67 | Administration of influenza vaccine | 0.13 (0.36) | <0.01 (0.02) | -0.36 |
| Body weight | 0.35 (0.59) | 0.01 (0.08) | -0.57 | Requires influenza virus vaccination | 0.1 (0.32) | <0.01 (0.03) | -0.32 |
| Body mass index (BMI) [Ratio] | 0.35 (0.59) | 0.01 (0.08) | -0.57 | 0.5 ML influenza A virus A/Idaho/07/2018 (H1N1) antigen 0.03 MG/ML / influenza A virus A/Indiana/08/2018 (H3N2) antigen 0.03 MG/ML / influenza B virus B/Iowa/06/2017 antigen 0.03 MG/ML… | 0.08 (0.28) | <0.01 (0.01) | -0.28 |
| Systolic blood pressure | 0.34 (0.58) | 0.01 (0.09) | -0.57 | Requires vaccination | 0.08 (0.29) | <0.01 (0.06) | -0.27 |
| Diastolic blood pressure | 0.34 (0.58) | 0.01 (0.09) | -0.57 | influenza, injectable, quadrivalent, contains preservative | 0.07 (0.27) | <0.01 (0.01) | -0.26 |
| Smoking status [FTND] | 0.32 (0.56) | 0.01 (0.09) | -0.54 | influenza, high dose seasonal, preservative-free | 0.07 (0.26) | <0.01 (0) | -0.26 |
| Pulse intensity of Unspecified artery palpation | 0.3 (0.55) | 0.01 (0.08) | -0.53 | Vaccine Administration | 0.06 (0.24) | <0.01 (0.04) | -0.23 |
| Body height | 0.29 (0.54) | 0.01 (0.08) | -0.52 | Immunization administration through 18 years of age via any route of administration, with counseling by physician or other qualified health care professional; first or only component of each vaccine or toxoid administered | 0.05 (0.22) | <0.01 (0.06) | -0.19 |
| Immunization administration (includes percutaneous, intradermal, subcutaneous, or intramuscular injections); 1 vaccine (single or combination vaccine/toxoid) | 0.26 (0.51) | <0.01 (0.01) | -0.51 | Immunization administration (includes percutaneous, intradermal, subcutaneous, or intramuscular injections); each additional vaccine (single or combination vaccine/toxoid) for primary procedure) | 0.03 (0.18) | <0.01 (0.03) | -0.17 |
| Influenza, injectable, quadrivalent, preservative free | 0.22 (0.47) | <0.01 (0) | -0.47 | Influenza, seasonal, injectable, preservative free | 0.03 (0.16) | <0.01 (0) | -0.16 |
| Body temperature | 0.24 (0.49) | <0.01 (0.07) | -0.47 | Drugs Identification - Drugs Requiring Detailed Coding | 0.07 (0.26) | 0.02 (0.16) | -0.14 |
| Temperature | 0.24 (0.49) | <0.01 (0.07) | -0.47 | influenza virus vaccine, whole virus | 0.02 (0.14) | <0.01 (0) | -0.14 |
| Alcohol intake | 0.21 (0.45) | <0.01 (0.07) | -0.44 | 0.5 ML Streptococcus pneumoniae type 1 capsular polysaccharide antigen 0.05 MG/ML / Streptococcus pneumoniae type 10A capsular polysaccharide antigen 0.05 MG/ML / Streptococcus pneumoniae type 11A capsular polysaccharide antigen 0.05 MG/ML / Streptococ... | 0.02 (0.14) | <0.01 (0.03) | -0.13 |
| Respiratory rate | 0.19 (0.43) | <0.01 (0.07) | -0.42 | General examination of patient | 0.04 (0.21) | 0.01 (0.12) | -0.13 |
| Oxygen [Partial pressure] in Blood | 0.16 (0.4) | <0.01 (0.07) | -0.38 | Influenza, injectable, Madin Darby Canine Kidney, preservative free, quadrivalent | 0.01 (0.12) | <0.01 (0) | -0.12 |
| Administration of influenza vaccine | 0.13 (0.37) | <0.01 (0) | -0.37 | 0.5 ML Streptococcus pneumoniae serotype 1 capsular antigen diphtheria CRM197 protein conjugate vaccine 0.0044 MG/ML / Streptococcus pneumoniae serotype 14 capsular antigen diphtheria CRM197 protein conjugate vaccine 0.0044 MG/ML / Streptococcus pneumo... | 0.02 (0.14) | <0.01 (0.05) | -0.12 |
| A detailed history; A detailed examination; Medical decision making of moderate complexity. Counseling and/o | 0.12 (0.35) | <0.01 (0.04) | -0.34 | Charlson index - Romano adaptation | 1.4 (2.34) | 1.04 (2.09) | -0.12 |
| Requires influenza virus vaccination | 0.1 (0.32) | <0.01 (0) | -0.32 | A detailed history; A detailed examination; Medical decision making of moderate complexity. Counseling and/o | 0.12 (0.35) | 0.07 (0.27) | -0.11 |
| Essential hypertension | 0.1 (0.32) | <0.01 (0.04) | -0.31 | Child examination | 0.02 (0.15) | 0.01 (0.07) | -0.11 |
| Requires vaccination | 0.08 (0.29) | <0.01 (0.01) | -0.29 |  |  |  |  |
| An expanded problem focused history; An expanded problem focused examination; Medical decision making of low | 0.09 (0.3) | <0.01 (0.05) | -0.29 |  |  |  |  |
| Collection of venous blood by venipuncture | 0.09 (0.29) | <0.01 (0.04) | -0.28 |  |  |  |  |
| Pain severity - 0-10 verbal numeric rating [Score] - Reported | 0.09 (0.29) | <0.01 (0.05) | -0.28 |  |  |  |  |
| 0.5 ML influenza A virus A/Idaho/07/2018 (H1N1) antigen 0.03 MG/ML / influenza A virus A/Indiana/08/2018 (H3N2) antigen 0.03 MG/ML / influenza B virus B/Iowa/06/2017 antigen 0.03 MG/ML / influenza B virus B/Singapore/INFTT-16-0610/2016 antigen 0.03 MG/... | 0.08 (0.28) | <0.01 (0) | -0.28 |  |  |  |  |
| Glucose [Mass/volume] in Serum or Plasma | 0.08 (0.28) | <0.01 (0.05) | -0.27 |  |  |  |  |
| influenza, injectable, quadrivalent, contains preservative | 0.07 (0.26) | <0.01 (0) | -0.26 |  |  |  |  |
| influenza, high dose seasonal, preservative-free | 0.07 (0.26) | <0.01 (0) | -0.26 |  |  |  |  |
| Cotinine/Creatinine [Mass Ratio] in Urine | 0.08 (0.27) | <0.01 (0.05) | -0.26 |  |  |  |  |
| Penicillin G potassium [Mass] of Dose | 0.07 (0.27) | <0.01 (0.05) | -0.26 |  |  |  |  |
| Sodium [Moles/volume] in Saliva (oral fluid) | 0.07 (0.27) | <0.01 (0.05) | -0.26 |  |  |  |  |
| Chloride [Moles/volume] in Saliva (oral fluid) | 0.07 (0.27) | <0.01 (0.05) | -0.26 |  |  |  |  |
| Calcium.ionized/Calcium.total corrected for albumin in Blood | 0.07 (0.27) | <0.01 (0.05) | -0.26 |  |  |  |  |
| Drugs Identification - Drugs Requiring Detailed Coding | 0.07 (0.26) | <0.01 (0.03) | -0.25 |  |  |  |  |
| Carbon dioxide [Partial pressure] in Blood | 0.07 (0.26) | <0.01 (0.05) | -0.25 |  |  |  |  |
| Leukocytes [#/volume] in Blood | 0.07 (0.26) | <0.01 (0.05) | -0.25 |  |  |  |  |
| Erythrocytes [#/volume] in Blood | 0.07 (0.26) | <0.01 (0.05) | -0.25 |  |  |  |  |
| Hemoglobin [Mass/volume] in Blood | 0.07 (0.26) | <0.01 (0.05) | -0.24 |  |  |  |  |
| Vaccine Administration | 0.06 (0.24) | <0.01 (0.01) | -0.24 |  |  |  |  |
| Hematocrit [Volume Fraction] of Blood | 0.06 (0.25) | <0.01 (0.05) | -0.24 |  |  |  |  |
| Platelets [#/volume] in Blood | 0.06 (0.25) | <0.01 (0.05) | -0.24 |  |  |  |  |
| MCV [Entitic volume] | 0.06 (0.25) | <0.01 (0.05) | -0.24 |  |  |  |  |
| MCHC [Mass/volume] | 0.06 (0.25) | <0.01 (0.05) | -0.24 |  |  |  |  |
| MCH [Entitic mass] | 0.06 (0.25) | <0.01 (0.05) | -0.24 |  |  |  |  |
| Platelet distribution width [Ratio] in Blood | 0.06 (0.25) | <0.01 (0.04) | -0.23 |  |  |  |  |
| Anion gap in Blood | 0.06 (0.24) | <0.01 (0.04) | -0.23 |  |  |  |  |
| Alanine aminotransferase [Enzymatic activity/volume] in Serum or Plasma | 0.06 (0.24) | <0.01 (0.04) | -0.23 |  |  |  |  |
| Lower proportion in vaccinated patients | | | | Lower proportion in vaccinated patients | | | |
|  |  |  |  | Visit occurrence concept count | 0.57 (0.23) | 1.06 (0.25) | 1.46 |
|  |  |  |  | Any visit on day 0 | 0.54 (0.73) | 1 (1) | 0.37 |
|  |  |  |  | Outpatient visit on day 0 | 0.52 (0.72) | 0.86 (0.93) | 0.29 |
|  |  |  |  | Emergency room visit on day 0 | 0.01 (0.08) | 0.08 (0.28) | 0.24 |
|  |  |  |  | Emergency Room - General Classification | 0.01 (0.07) | 0.04 (0.2) | 0.16 |
|  |  |  |  | A detailed history; A detailed examination; and Medical decision making of moderate complexity. Counseling and/or coordination of care with o | <0.01 (0.03) | 0.03 (0.16) | 0.15 |
|  |  |  |  | An expanded problem focused history; An expanded problem focused examination; and Medical decision making of moderate complexity. Counseling | <0.01 (0.02) | 0.02 (0.15) | 0.15 |
|  |  |  |  | Infectious agent antigen detection by immunoassay with direct optical observation; Streptococcus, group A | <0.01 (0.05) | 0.02 (0.14) | 0.11 |
|  |  |  |  | Acute pharyngitis | <0.01 (0.05) | 0.02 (0.14) | 0.11 |

**Table S4.** Top 50 covariates with the standardized difference of means (SDM) >0.1, covariate proportion and standard deviation, for comparison of COVID-19 vaccinated population indexed on the date of vaccination compared to the same population indexed on (a) a date or (b) a visit, day 0.

| **COVID-19 vaccinated patients (Target) compared to unvaccinated indexed on a date (Comparator)** | | | | **COVID-19 vaccinated patients (Target) compared to unvaccinated indexed on a visit (Comparator)** | | | |
| --- | --- | --- | --- | --- | --- | --- | --- |
| **Covariate name** | **Target, mean (SD)** | **Comparator, mean (SD)** | **SDM** | **Covariate name** | **Target, mean (SD)** | **Comparator, mean (SD)** | **SDM** |
| ***CUIMC EHR*** | | | | | | | |
| Higher proportion before vaccination | | | | Higher proportion before vaccination | | | |
| SARS-CoV-2 (COVID-19) vaccine, mRNA-BNT162b2 0.1 MG/ML Injectable Suspension | 0.68 (0.46) | <0.01 (0) | -2.08 | SARS-CoV-2 (COVID-19) vaccine, mRNA-BNT162b2 0.1 MG/ML Injectable Suspension | 0.67 (0.47) | <0.01 (0) | -2.03 |
| Patient encounter status | 0.47 (0.5) | <0.01 (0.04) | -1.31 | SARS-CoV-2 (COVID-19) vaccine, mRNA-1273 0.2 MG/ML Injectable Suspension | 0.3 (0.46) | <0.01 (0) | -0.93 |
| SARS-CoV-2 (COVID-19) vaccine, mRNA-1273 0.2 MG/ML Injectable Suspension | 0.29 (0.45) | <0.01 (0) | -0.91 | Patient encounter status | 0.45 (0.5) | 0.13 (0.34) | -0.76 |
| index month:1 | 0.29 (0.45) | 0.1 (0.3) | -0.49 | index month:1 | 0.3 (0.46) | 0.11 (0.31) | -0.48 |
| index month:3 | 0.27 (0.45) | 0.11 (0.32) | -0.42 | index month:3 | 0.26 (0.44) | 0.1 (0.31) | -0.42 |
| index month:2 | 0.21 (0.4) | 0.1 (0.3) | -0.29 | index month:4 | 0.14 (0.35) | 0.06 (0.24) | -0.27 |
| SARS-COV-2 (COVID-19) vaccine, vector non-replicating, recombinant spike protein-Ad26, preservative free, 0.5 mL | 0.02 (0.15) | <0.01 (0) | -0.22 | SARS-COV-2 (COVID-19) vaccine, vector non-replicating, recombinant spike protein-Ad26, preservative free, 0.5 mL | 0.03 (0.16) | <0.01 (0) | -0.23 |
| Outpatient visit on day 0 | 0.02 (0.14) | 0.01 (0.08) | -0.12 | index month:2 | 0.21 (0.41) | 0.14 (0.34) | -0.19 |
| Any visit on day 0 | 0.02 (0.15) | 0.01 (0.09) | -0.12 |  |  |  |  |
| index month:4 | 0.15 (0.35) | 0.11 (0.31) | -0.12 |  |  |  |  |
| Lower proportion before vaccination | | | | Lower proportion before vaccination | | | |
| index month:6 | <0.01 (0) | 0.11 (0.32) | 0.5 | Any visit on day 0 | 0.03 (0.18) | 1 (0) | 7.47 |
| index month:7 | <0.01 (0) | 0.1 (0.3) | 0.47 | Outpatient visit on day 0 | 0.03 (0.17) | 0.71 (0.45) | 1.98 |
| index month:5 | 0.01 (0.11) | 0.12 (0.32) | 0.43 | Visit occurrence concept count | 0.05 (0.52) | 1.21 (0.55) | 1.54 |
| index month:8 | <0.01 (0) | 0.07 (0.26) | 0.39 | Systolic blood pressure | 0.02 (0.14) | 0.42 (0.49) | 1.11 |
| index month:11 | <0.01 (0) | 0.04 (0.2) | 0.3 | Diastolic blood pressure | 0.02 (0.14) | 0.42 (0.49) | 1.11 |
| index month:9 | <0.01 (0) | 0.04 (0.18) | 0.27 | Heart rate | 0.02 (0.13) | 0.36 (0.48) | 0.97 |
| index month:10 | <0.01 (0) | 0.02 (0.16) | 0.22 | Body weight | 0.02 (0.13) | 0.35 (0.48) | 0.95 |
|  |  |  |  | Tobacco smoking behavior - finding | 0.01 (0.12) | 0.34 (0.47) | 0.93 |
|  |  |  |  | Body mass index (BMI) [Ratio] | 0.02 (0.12) | 0.34 (0.47) | 0.93 |
|  |  |  |  | Body height | 0.01 (0.11) | 0.26 (0.44) | 0.77 |
|  |  |  |  | Body temperature | 0.02 (0.12) | 0.26 (0.44) | 0.76 |
|  |  |  |  | Oxygen saturation in Arterial blood by Pulse oximetry | 0.01 (0.12) | 0.2 (0.4) | 0.64 |
|  |  |  |  | Respiratory rate | 0.01 (0.11) | 0.18 (0.39) | 0.6 |
|  |  |  |  | A detailed history; A detailed examination; Medical decision making of moderate complexity. Counseling and/o | 0.01 (0.08) | 0.17 (0.37) | 0.6 |
|  |  |  |  | An expanded problem focused history; An expanded problem focused examination; Medical decision making of low | 0.01 (0.07) | 0.15 (0.36) | 0.57 |
|  |  |  |  | Calcium [Mass/volume] in Serum or Plasma | 0.01 (0.1) | 0.15 (0.36) | 0.53 |
|  |  |  |  | Creatinine [Mass/volume] in Serum or Plasma | 0.01 (0.1) | 0.15 (0.36) | 0.53 |
|  |  |  |  | Hemoglobin [Mass/volume] in Blood | 0.01 (0.1) | 0.15 (0.36) | 0.53 |
|  |  |  |  | Urea nitrogen [Mass/volume] in Serum or Plasma | 0.01 (0.1) | 0.15 (0.36) | 0.53 |
|  |  |  |  | Potassium [Moles/volume] in Serum or Plasma | 0.01 (0.1) | 0.15 (0.36) | 0.53 |
|  |  |  |  | Chloride [Moles/volume] in Serum or Plasma | 0.01 (0.1) | 0.15 (0.36) | 0.53 |
|  |  |  |  | Carbon dioxide, total [Moles/volume] in Serum or Plasma | 0.01 (0.1) | 0.15 (0.36) | 0.53 |
|  |  |  |  | Sodium [Moles/volume] in Serum or Plasma | 0.01 (0.1) | 0.15 (0.36) | 0.53 |
|  |  |  |  | Hematocrit [Volume Fraction] of Blood by Automated count | 0.01 (0.1) | 0.15 (0.35) | 0.53 |
|  |  |  |  | Erythrocyte distribution width [Ratio] by Automated count | 0.01 (0.1) | 0.15 (0.35) | 0.53 |
|  |  |  |  | MCHC [Mass/volume] by Automated count | 0.01 (0.1) | 0.15 (0.35) | 0.53 |
|  |  |  |  | MCH [Entitic mass] by Automated count | 0.01 (0.1) | 0.15 (0.35) | 0.53 |
|  |  |  |  | MCV [Entitic volume] by Automated count | 0.01 (0.1) | 0.15 (0.35) | 0.53 |
|  |  |  |  | Leukocytes [#/volume] in Blood by Automated count | 0.01 (0.1) | 0.15 (0.35) | 0.53 |
|  |  |  |  | Erythrocytes [#/volume] in Blood by Automated count | 0.01 (0.1) | 0.15 (0.35) | 0.53 |
|  |  |  |  | Platelets [#/volume] in Blood by Automated count | 0.01 (0.1) | 0.15 (0.35) | 0.53 |
|  |  |  |  | Anion gap in Serum or Plasma | 0.01 (0.1) | 0.15 (0.35) | 0.52 |
|  |  |  |  | index month:7 | <0.01 (0) | 0.12 (0.32) | 0.52 |
|  |  |  |  | Glomerular filtration rate/1.73 sq M.predicted [Volume Rate/Area] in Serum, Plasma or Blood by Creatinine-based formula (MDRD) | 0.01 (0.1) | 0.15 (0.35) | 0.52 |
|  |  |  |  | Platelet mean volume [Entitic volume] in Blood by Automated count | 0.01 (0.1) | 0.14 (0.35) | 0.52 |
|  |  |  |  | index month:6 | <0.01 (0) | 0.12 (0.32) | 0.52 |
|  |  |  |  | Alanine aminotransferase [Enzymatic activity/volume] in Serum or Plasma | 0.01 (0.09) | 0.12 (0.33) | 0.48 |
|  |  |  |  | Aspartate aminotransferase [Enzymatic activity/volume] in Serum or Plasma | 0.01 (0.09) | 0.12 (0.33) | 0.48 |
|  |  |  |  | Protein [Mass/volume] in Serum or Plasma | 0.01 (0.09) | 0.12 (0.33) | 0.47 |
|  |  |  |  | Albumin [Mass/volume] in Serum or Plasma | 0.01 (0.09) | 0.12 (0.33) | 0.47 |
|  |  |  |  | Alkaline phosphatase [Enzymatic activity/volume] in Serum or Plasma | 0.01 (0.09) | 0.12 (0.33) | 0.47 |
|  |  |  |  | Bilirubin.total [Mass/volume] in Serum or Plasma | 0.01 (0.09) | 0.12 (0.33) | 0.47 |
|  |  |  |  | Nucleated erythrocytes/100 leukocytes [Ratio] in Blood by Automated count | 0.01 (0.1) | 0.12 (0.33) | 0.47 |
|  |  |  |  | Nucleated erythrocytes [#/volume] in Blood by Automated count | 0.01 (0.1) | 0.12 (0.33) | 0.47 |
|  |  |  |  | Essential hypertension | 0.01 (0.08) | 0.11 (0.32) | 0.47 |
|  |  |  |  | Lymphocytes [#/volume] in Blood by Automated count | 0.01 (0.08) | 0.11 (0.32) | 0.46 |
|  |  |  |  | Basophils/100 leukocytes in Blood by Automated count | 0.01 (0.08) | 0.11 (0.32) | 0.46 |
|  |  |  |  | Neutrophils [#/volume] in Blood by Automated count | 0.01 (0.08) | 0.11 (0.32) | 0.46 |
| ***Optum EHR*** | | | | | | | |
| Higher proportion before vaccination | | | | Higher proportion before vaccination | | | |
| Visit occurrence concept count | 0.73 (0.05) | 0.07 (0.17) | -3.69 | SARS-CoV-2 (COVID-19) vaccine, mRNA-BNT162b2 0.1 MG/ML Injectable Suspension | 0.95 (0.98) | <0.01 (0) | -0.98 |
| SARS-CoV-2 (COVID-19) vaccine, mRNA-BNT162b2 0.1 MG/ML Injectable Suspension | 0.95 (0.98) | <0.01 (0) | -0.98 | SARS-CoV-2 (COVID-19) vaccine, mRNA-1273 0.2 MG/ML Injectable Suspension | 0.33 (0.58) | <0.01 (0) | -0.58 |
| Patient encounter procedure | 0.61 (0.78) | 0.01 (0.08) | -0.77 | Patient encounter procedure | 0.61 (0.78) | 0.14 (0.38) | -0.54 |
| Outpatient visit on day 0 | 0.72 (0.85) | 0.06 (0.24) | -0.76 | Requires vaccination | 0.17 (0.41) | 0.01 (0.08) | -0.39 |
| Any visit on day 0 | 0.73 (0.85) | 0.06 (0.25) | -0.75 | Vaccine Administration | 0.14 (0.37) | <0.01 (0.05) | -0.36 |
| SARS-CoV-2 (COVID-19) vaccine, mRNA-1273 0.2 MG/ML Injectable Suspension | 0.33 (0.58) | <0.01 (0) | -0.58 | index month:3 | 0.3 (0.54) | 0.1 (0.32) | -0.3 |
| Requires vaccination | 0.17 (0.41) | <0.01 (0.01) | -0.41 | index month:1 | 0.29 (0.54) | 0.11 (0.33) | -0.29 |
| Vaccine Administration | 0.14 (0.37) | <0.01 (0.01) | -0.37 | index month:2 | 0.25 (0.5) | 0.1 (0.31) | -0.26 |
| index month:1 | 0.3 (0.54) | 0.11 (0.34) | -0.29 | index month:12 | 0.16 (0.4) | 0.08 (0.28) | -0.17 |
| Drugs Identification - Drugs Requiring Detailed Coding | 0.09 (0.3) | <0.01 (0.05) | -0.28 | SARS-COV-2 (COVID-19) vaccine, UNSPECIFIED | 0.03 (0.17) | <0.01 (0) | -0.17 |
| index month:3 | 0.29 (0.54) | 0.11 (0.34) | -0.28 | Drugs Identification - Drugs Requiring Detailed Coding | 0.09 (0.3) | 0.03 (0.18) | -0.16 |
| index month:2 | 0.25 (0.5) | 0.11 (0.33) | -0.24 |  |  |  |  |
| Pharmacy - General Classification | 0.05 (0.22) | <0.01 (0.05) | -0.21 |  |  |  |  |
| SARS-COV-2 (COVID-19) vaccine, UNSPECIFIED | 0.03 (0.17) | <0.01 (0) | -0.17 |  |  |  |  |
| index month:12 | 0.16 (0.4) | 0.09 (0.3) | -0.15 |  |  |  |  |
| Lower proportion before vaccination | | | | Lower proportion before vaccination | | | |
| index month:5 | <0.01 (0) | 0.11 (0.34) | 0.34 | Visit occurrence concept count | 0.73 (0.05) | 1.04 (0.22) | 1.38 |
| index month:4 | <0.01 (0) | 0.11 (0.33) | 0.33 | Smoking status [FTND] | 0.02 (0.15) | 0.26 (0.51) | 0.45 |
| index month:6 | <0.01 (0) | 0.11 (0.33) | 0.33 | Diastolic blood pressure | 0.02 (0.15) | 0.23 (0.47) | 0.41 |
| index month:7 | <0.01 (0) | 0.09 (0.29) | 0.29 | Systolic blood pressure | 0.02 (0.15) | 0.22 (0.47) | 0.4 |
| index month:11 | <0.01 (0.01) | 0.06 (0.24) | 0.24 | Body mass index (BMI) [Ratio] | 0.02 (0.14) | 0.22 (0.46) | 0.4 |
| index month:8 | <0.01 (0.02) | 0.05 (0.23) | 0.23 | Body weight | 0.02 (0.15) | 0.22 (0.47) | 0.4 |
| index month:10 | <0.01 (0.01) | 0.03 (0.16) | 0.16 | Alcohol intake | 0.01 (0.12) | 0.19 (0.44) | 0.39 |
| index month:9 | <0.01 (0.01) | 0.02 (0.14) | 0.14 | Pulse intensity of Unspecified artery palpation | 0.02 (0.15) | 0.21 (0.46) | 0.39 |
|  |  |  |  | Body height | 0.02 (0.13) | 0.19 (0.43) | 0.38 |
|  |  |  |  | index month:6 | <0.01 (0) | 0.14 (0.37) | 0.37 |
|  |  |  |  | index month:5 | <0.01 (0) | 0.11 (0.33) | 0.33 |
|  |  |  |  | index month:7 | <0.01 (0) | 0.11 (0.33) | 0.33 |
|  |  |  |  | Body temperature | 0.02 (0.16) | 0.16 (0.4) | 0.32 |
|  |  |  |  | Temperature | 0.02 (0.16) | 0.16 (0.4) | 0.32 |
|  |  |  |  | Oxygen [Partial pressure] in Blood | 0.02 (0.13) | 0.14 (0.38) | 0.32 |
|  |  |  |  | Respiratory rate | 0.01 (0.11) | 0.13 (0.36) | 0.31 |
|  |  |  |  | index month:4 | <0.01 (0) | 0.09 (0.3) | 0.3 |
|  |  |  |  | index month:8 | <0.01 (0.01) | 0.06 (0.25) | 0.25 |
|  |  |  |  | Essential hypertension | 0.01 (0.09) | 0.08 (0.29) | 0.25 |
|  |  |  |  | Collection of venous blood by venipuncture | 0.01 (0.09) | 0.08 (0.28) | 0.24 |
|  |  |  |  | Pain severity - 0-10 verbal numeric rating [Score] - Reported | <0.01 (0.07) | 0.07 (0.26) | 0.24 |
|  |  |  |  | A detailed history; A detailed examination; Medical decision making of moderate complexity. Counseling and/o | 0.01 (0.09) | 0.07 (0.27) | 0.23 |
|  |  |  |  | Glucose [Mass/volume] in Serum or Plasma | 0.01 (0.1) | 0.08 (0.28) | 0.23 |
|  |  |  |  | index month:11 | <0.01 (0.01) | 0.05 (0.23) | 0.23 |
|  |  |  |  | An expanded problem focused history and examination | 0.01 (0.07) | 0.07 (0.26) | 0.23 |
|  |  |  |  | Cotinine/Creatinine [Mass Ratio] in Urine | 0.01 (0.1) | 0.08 (0.28) | 0.22 |
|  |  |  |  | Penicillin G potassium [Mass] of Dose | 0.01 (0.1) | 0.07 (0.27) | 0.22 |
|  |  |  |  | Sodium [Moles/volume] in Saliva (oral fluid) | 0.01 (0.1) | 0.07 (0.27) | 0.22 |
|  |  |  |  | Chloride [Moles/volume] in Saliva (oral fluid) | 0.01 (0.1) | 0.07 (0.27) | 0.22 |
|  |  |  |  | Calcium.ionized/Calcium.total corrected for albumin in Blood | 0.01 (0.1) | 0.07 (0.27) | 0.22 |
|  |  |  |  | Leukocytes [#/volume] in Blood | 0.01 (0.1) | 0.07 (0.27) | 0.22 |
|  |  |  |  | Erythrocytes [#/volume] in Blood | 0.01 (0.1) | 0.07 (0.27) | 0.22 |
|  |  |  |  | Hemoglobin [Mass/volume] in Blood | 0.01 (0.09) | 0.07 (0.26) | 0.21 |
|  |  |  |  | Hematocrit [Volume Fraction] of Blood | 0.01 (0.09) | 0.07 (0.26) | 0.21 |
|  |  |  |  | Exposure to viral disease | <0.01 (0.05) | 0.05 (0.23) | 0.21 |
|  |  |  |  | Carbon dioxide [Partial pressure] in Blood | 0.01 (0.1) | 0.07 (0.26) | 0.21 |
|  |  |  |  | Platelets [#/volume] in Blood | 0.01 (0.09) | 0.07 (0.26) | 0.21 |
|  |  |  |  | MCV [Entitic volume] | 0.01 (0.09) | 0.07 (0.26) | 0.21 |
|  |  |  |  | MCHC [Mass/volume] | 0.01 (0.09) | 0.07 (0.26) | 0.21 |
|  |  |  |  | Platelet distribution width [Ratio] in Blood | 0.01 (0.09) | 0.07 (0.26) | 0.21 |
|  |  |  |  | MCH [Entitic mass] | 0.01 (0.09) | 0.07 (0.26) | 0.21 |
|  |  |  |  | Any visit on day 0 | 0.73 (0.85) | 1 (1) | 0.21 |
|  |  |  |  | Laboratory - General Classification | 0.01 (0.08) | 0.06 (0.25) | 0.21 |
|  |  |  |  | Laboratory - Chemistry | 0.01 (0.08) | 0.06 (0.24) | 0.2 |
|  |  |  |  | Anion gap in Blood | 0.01 (0.09) | 0.06 (0.25) | 0.2 |
|  |  |  |  | Neutrophil cytoplasmic Ab.perinuclear [Presence] in Serum | 0.01 (0.09) | 0.06 (0.24) | 0.19 |
|  |  |  |  | Lymphocytes/100 leukocytes in Blood | 0.01 (0.09) | 0.06 (0.24) | 0.19 |
|  |  |  |  | Neutrophil Ab [Presence] in Serum | 0.01 (0.09) | 0.06 (0.24) | 0.19 |

**Table S5.** Top 50 covariates with the standardized difference of means (SDM) >0.1, covariate proportion and standard deviation, for comparison of influenza vaccinated population indexed on the date of vaccination compared to the same population indexed on (a) a date or (b) a visit, day 0.

| **Influenza vaccinated patients (Target) compared to unvaccinated indexed on a date (Comparator)** | | | | **Influenza vaccinated patients (Target) compared to unvaccinated indexed on a visit (Comparator)** | | | |
| --- | --- | --- | --- | --- | --- | --- | --- |
| **Covariate name** | **Target, mean (SD)** | **Comparator, mean (SD)** | **SDM** | **Covariate name** | **Target, mean (SD)** | **Comparator, mean (SD)** | **SDM** |
| ***CUIMC EHR*** | | | | | | | |
| Higher proportion before vaccination | | | | Higher proportion before vaccination | | | |
| index month:6 | 0.11 (0.32) | 0.04 (0.19) | -0.29 | Influenza, injectable, quadrivalent, preservative free | 0.42 (0.49) | 0.09 (0.28) | -0.84 |
| index month:5 | 0.12 (0.32) | 0.05 (0.22) | -0.23 | 0.5 ML influenza A virus A/Hong Kong/4801/2014 (H3N2) antigen 0.03 MG/ML / influenza A virus A/Michigan/45/2015 (H1N1) antigen 0.03 MG/ML / influenza B virus B/Brisbane/60/2008 antigen 0.03 MG/ML... | 0.2 (0.4) | <0.01 (0.01) | -0.7 |
| index month:7 | 0.1 (0.3) | 0.06 (0.23) | -0.17 | influenza virus vaccine, unspecified formulation | 0.17 (0.38) | <0.01 (0) | -0.65 |
| age group: 70 - 74 | 0.1 (0.3) | 0.06 (0.23) | -0.16 | Platelet mean volume [Entitic volume] in Blood by Automated count | 0.18 (0.38) | <0.01 (0.07) | -0.63 |
| age group: 65 - 69 | 0.11 (0.31) | 0.06 (0.24) | -0.16 | Platelets [#/volume] in Blood by Automated count | 0.19 (0.39) | 0.01 (0.09) | -0.63 |
| age group: 40 - 44 | 0.07 (0.25) | 0.04 (0.19) | -0.14 | Glomerular filtration rate/1.73 sq M.predicted [Volume Rate/Area] in Serum, Plasma or Blood by Creatinine-based formula (MDRD) | 0.14 (0.35) | <0.01 (0.05) | -0.56 |
| age group: 35 - 39 | 0.08 (0.27) | 0.05 (0.22) | -0.13 | 0.5 ML influenza A virus A/Hong Kong/4801/2014 (H3N2) antigen 0.03 MG/ML / influenza A virus A/Singapore/GP1908/2015 (H1N1) antigen 0.03 MG/ML... | 0.13 (0.34) | 0.01 (0.09) | -0.49 |
| age group: 55 - 59 | 0.08 (0.27) | 0.05 (0.22) | -0.12 | index month:10 | 0.29 (0.45) | 0.1 (0.3) | -0.49 |
| age group: 60 - 64 | 0.09 (0.29) | 0.06 (0.24) | -0.12 | Glomerular filtration rate/1.73 sq M.predicted [Volume Rate/Area] in Serum, Plasma or Blood | 0.1 (0.31) | <0.01 (0.06) | -0.46 |
| age group: 50 - 54 | 0.07 (0.26) | 0.04 (0.21) | -0.12 | Patient encounter procedure | 0.41 (0.49) | 0.22 (0.42) | -0.41 |
| age group: 75 - 79 | 0.07 (0.25) | 0.04 (0.2) | -0.11 | influenza, high dose seasonal, preservative-free | 0.1 (0.29) | 0.01 (0.09) | -0.4 |
| age group: 45 - 49 | 0.06 (0.24) | 0.04 (0.19) | -0.11 | 0.5 ML influenza A virus A/Hong Kong/4801/2014 (H3N2) antigen 0.12 MG/ML / influenza A virus A/Michigan/45/2015 (H1N1) antigen 0.12 MG/ML / influenza B virus B/Brisbane/60/2008 antigen 0.12 MG/ML Prefilled Syringe [Fluzone 2017-2018] | 0.08 (0.27) | 0.01 (0.07) | -0.38 |
| index month:4 | 0.11 (0.31) | 0.08 (0.27) | -0.11 | Immunization administration (includes percutaneous, intradermal, subcutaneous, or intramuscular injections); 1 vaccine (single or combination vaccine/toxoid) | 0.08 (0.26) | 0.01 (0.1) | -0.33 |
| age group: 30 - 34 | 0.08 (0.26) | 0.05 (0.22) | -0.11 | Neisseria gonorrhoeae DNA [Presence] in Unspecified specimen by NAA with probe detection | 0.05 (0.22) | <0.01 (0.04) | -0.32 |
|  |  |  |  | Specific gravity of Urine by Test strip | 0.06 (0.24) | <0.01 (0.07) | -0.32 |
|  |  |  |  | Follow-up encounter | 0.18 (0.38) | 0.08 (0.27) | -0.31 |
|  |  |  |  | Administration of influenza vaccine | 0.06 (0.23) | 0.01 (0.09) | -0.28 |
|  |  |  |  | index month:9 | 0.18 (0.38) | 0.09 (0.28) | -0.27 |
|  |  |  |  | Body height Measured | 0.4 (0.49) | 0.27 (0.45) | -0.26 |
|  |  |  |  | Tobacco smoking behavior - finding | 0.44 (0.5) | 0.32 (0.47) | -0.25 |
|  |  |  |  | 0.5 ML influenza A virus A/Michigan/45/2015 (H1N1) antigen 0.03 MG/ML / influenza A virus A/Singapore/INFIMH-16-0019/2016 (H3N2) antigen 0.03 MG/ML... | 0.03 (0.18) | <0.01 (0.03) | -0.25 |
|  |  |  |  | Patient encounter status | 0.03 (0.17) | <0.01 (0.02) | -0.24 |
|  |  |  |  | Vaccination needed | 0.03 (0.16) | <0.01 (0.01) | -0.24 |
|  |  |  |  | index month:11 | 0.19 (0.39) | 0.1 (0.31) | -0.23 |
|  |  |  |  | influenza A virus A/California/7/2009 (H1N1) antigen 0.03 MG/ML / influenza A virus A/South Australia/55/2014 (H3N2) antigen 0.03 MG/ML / influenza B virus B/Phuket/3073/2013 antigen 0.03 MG/ML Injectable Suspension [Afluria 2015-2016] | 0.03 (0.16) | <0.01 (0) | -0.23 |
|  |  |  |  | 0.5 ML influenza A virus A/Michigan/45/2015 (H1N1) antigen 0.12 MG/ML / influenza A virus A/Singapore/INFIMH-16-0019/2016 (H3N2) antigen 0.12 MG/ML / influenza B virus B/Maryland/15/2016 antigen 0.12 MG/ML Prefilled Syringe [Fluzone 2018-2019] | 0.04 (0.18) | <0.01 (0.06) | -0.23 |
|  |  |  |  | Lead [Mass/volume] in Venous blood | 0.03 (0.16) | <0.01 (0.03) | -0.23 |
|  |  |  |  | Body weight | 0.65 (0.48) | 0.54 (0.5) | -0.21 |
|  |  |  |  | Body mass index (BMI) [Ratio] | 0.58 (0.49) | 0.48 (0.5) | -0.21 |
|  |  |  |  | Needs influenza immunization | 0.02 (0.14) | <0.01 (0.01) | -0.2 |
|  |  |  |  | Influenza, seasonal, injectable, preservative free | 0.02 (0.14) | <0.01 (0.01) | -0.19 |
|  |  |  |  | 0.5 ML influenza A virus A/Idaho/07/2018 (H1N1) antigen 0.03 MG/ML / influenza A virus A/Indiana/08/2018 (H3N2) antigen 0.03 MG/ML… | 0.02 (0.13) | <0.01 (0.01) | -0.19 |
|  |  |  |  | 0.5 ML influenza A virus A/Brisbane/02/2018 (H1N1) antigen 0.03 MG/ML / influenza A virus A/Kansas/14/2017 (H3N2) antigen 0.03 MG/ML / influenza B virus B/Maryland/... | 0.02 (0.13) | <0.01 (0) | -0.18 |
|  |  |  |  | Rubella virus IgG Ab [Presence] in Serum or Plasma by Immunoassay | 0.01 (0.11) | <0.01 (0.02) | -0.16 |
|  |  |  |  | C reactive protein [Mass/volume] in Serum or Plasma by High sensitivity method | 0.01 (0.11) | <0.01 (0.01) | -0.15 |
|  |  |  |  | Urinalysis dipstick W Reflex Microscopic panel - Urine | 0.02 (0.12) | <0.01 (0.03) | -0.15 |
|  |  |  |  | Blood typing, serologic; ABO | 0.01 (0.11) | <0.01 (0.02) | -0.15 |
|  |  |  |  | ABO and Rh group [Type] in Blood | 0.01 (0.11) | <0.01 (0.02) | -0.15 |
|  |  |  |  | Cholesterol in LDL [Mass/volume] in Serum or Plasma by calculation | 0.1 (0.3) | 0.06 (0.23) | -0.15 |
|  |  |  |  | Immunization status | 0.01 (0.11) | <0.01 (0.01) | -0.15 |
|  |  |  |  | CD19 cells/100 cells in Blood | 0.01 (0.1) | <0.01 (0.01) | -0.15 |
|  |  |  |  | Urinalysis complete panel - Urine | 0.01 (0.11) | <0.01 (0.03) | -0.15 |
|  |  |  |  | Cholesterol in HDL [Mass/volume] in Serum or Plasma | 0.1 (0.3) | 0.06 (0.24) | -0.15 |
|  |  |  |  | Triglyceride [Mass/volume] in Serum or Plasma | 0.1 (0.3) | 0.06 (0.24) | -0.15 |
|  |  |  |  | Cholesterol [Mass/volume] in Serum or Plasma | 0.1 (0.3) | 0.06 (0.24) | -0.14 |
|  |  |  |  | Cholesterol.total/Cholesterol in HDL [Mass Ratio] in Serum or Plasma | 0.1 (0.3) | 0.06 (0.24) | -0.14 |
|  |  |  |  | Body height [Percentile] | 0.18 (0.39) | 0.13 (0.34) | -0.14 |
|  |  |  |  | Hemoglobin A1c/Hemoglobin.total in Blood | 0.11 (0.31) | 0.07 (0.25) | -0.14 |
| Lower proportion before vaccination | | | | Lower proportion before vaccination | | | |
| age group: 0 - 4 | <0.01 (0) | 0.1 (0.3) | 0.48 | Any visit on day 0 | 0.77 (0.42) | 1 (0) | 0.76 |
| age group: 5-9 | <0.01 (0) | 0.1 (0.3) | 0.46 | Emergency room visit on day 0 | <0.01 (0.04) | 0.1 (0.3) | 0.45 |
| age group: 10-14 | <0.01 (0.04) | 0.1 (0.3) | 0.46 | Outpatient visit on day 0 | 0.77 (0.42) | 0.91 (0.29) | 0.37 |
| index month:10 | 0.02 (0.16) | 0.09 (0.29) | 0.29 | An expanded problem focused history; An expanded problem focused examination; and Medical decision making of moderate complexity. Counseling | <0.01 (0.02) | 0.05 (0.22) | 0.33 |
| age group: 15-19 | 0.02 (0.14) | 0.07 (0.26) | 0.26 | index month:3 | 0.03 (0.17) | 0.11 (0.32) | 0.32 |
| index month:11 | 0.04 (0.2) | 0.1 (0.3) | 0.22 | A detailed history; A detailed examination; and Medical decision making of moderate complexity. Counseling and/or coordination of care with o | <0.01 (0.03) | 0.05 (0.22) | 0.32 |
| index month:9 | 0.04 (0.18) | 0.08 (0.27) | 0.19 | Dry body weight Measured | 0.01 (0.08) | 0.06 (0.24) | 0.3 |
| index month:12 | 0.08 (0.26) | 0.11 (0.31) | 0.11 | index month:7 | <0.01 (0.04) | 0.05 (0.21) | 0.3 |
|  |  |  |  | index month:5 | 0.01 (0.07) | 0.06 (0.23) | 0.3 |
|  |  |  |  | index month:4 | 0.01 (0.12) | 0.07 (0.26) | 0.29 |
|  |  |  |  | Visit occurrence concept count | 1.01 (0.78) | 1.32 (0.73) | 0.29 |
|  |  |  |  | index month:6 | <0.01 (0.05) | 0.04 (0.19) | 0.25 |
|  |  |  |  | index month:8 | 0.02 (0.15) | 0.07 (0.25) | 0.22 |
|  |  |  |  | A comprehensive history examination | <0.01 (0.03) | 0.02 (0.16) | 0.22 |
|  |  |  |  | Bacteria identified in Urine by Culture | <0.01 (0.06) | 0.03 (0.17) | 0.21 |
|  |  |  |  | Fever | <0.01 (0.04) | 0.02 (0.14) | 0.18 |
|  |  |  |  | Child examination | <0.01 (0.01) | 0.02 (0.12) | 0.18 |
|  |  |  |  | Respiratory rate | 0.18 (0.39) | 0.26 (0.44) | 0.18 |
|  |  |  |  | Bacteria identified in Throat by Culture | <0.01 (0.02) | 0.01 (0.12) | 0.17 |
|  |  |  |  | Child weight centiles - finding | 0.04 (0.19) | 0.08 (0.27) | 0.17 |
|  |  |  |  | Electrocardiogram, routine ECG with at least 12 leads; interpretation and report only | 0.01 (0.1) | 0.03 (0.18) | 0.16 |
|  |  |  |  | Inpatient visit on day 0 | <0.01 (0.03) | 0.01 (0.12) | 0.15 |
|  |  |  |  | Child height centile finding | 0.04 (0.19) | 0.07 (0.26) | 0.15 |
|  |  |  |  | An expanded problem focused history; An expanded problem focused examination; Medical decision making of low | 0.03 (0.17) | 0.06 (0.24) | 0.14 |
|  |  |  |  | 1000 ML sodium chloride 9 MG/ML Injection | <0.01 (0.05) | 0.01 (0.12) | 0.14 |
|  |  |  |  | No known drug allergy | <0.01 (0.05) | 0.01 (0.12) | 0.14 |
|  |  |  |  | Therapeutic, prophylactic, or diagnostic injection (specify substance or drug); intravenous push, single or initial substance/drug | <0.01 (0.01) | 0.01 (0.1) | 0.13 |
|  |  |  |  | Microscopic exam [Interpretation] of Urine by Cytology | 0.03 (0.16) | 0.05 (0.22) | 0.13 |
|  |  |  |  | Body surface area | 0.16 (0.36) | 0.21 (0.41) | 0.13 |
|  |  |  |  | Radiologic examination, chest, 2 views, frontal and lateral | 0.01 (0.08) | 0.02 (0.15) | 0.13 |
|  |  |  |  | Body mass index (BMI) [Percentile] | 0.04 (0.18) | 0.06 (0.24) | 0.13 |
|  |  |  |  | A detailed history; A detailed examination; Medical decision making of low complexity. Counseling and/or coordination of care with | <0.01 (0.04) | 0.01 (0.11) | 0.13 |
|  |  |  |  | An expanded problem focused history; An expanded problem focused examination; and Medical decision making of low complexity. Counseling and/o | <0.01 (0.01) | 0.01 (0.09) | 0.12 |
|  |  |  |  | Level IV - Surgical pathology, gross and microscopic examination Abortion - spontaneous/missed Artery, biopsy Bone marrow, biopsy Bone exostosis Brain/meninges, other than for tumor resection Breast, biopsy, not requiring microscopic evaluation of surgica | <0.01 (0.03) | 0.01 (0.1) | 0.12 |
|  |  |  |  | Sodium [Moles/volume] in Blood | <0.01 (0.02) | 0.01 (0.09) | 0.12 |
|  |  |  |  | Potassium [Moles/volume] in Blood | <0.01 (0.02) | 0.01 (0.09) | 0.12 |
|  |  |  |  | amoxicillin 80 MG/ML Oral Suspension | <0.01 (0.06) | 0.01 (0.12) | 0.11 |
|  |  |  |  | Pressurized or nonpressurized inhalation treatment for acute airway obstruction for therapeutic purposes and/or for diagnostic purposes such as sputum induction with an aerosol generator, nebulizer, metered dose inhaler or intermittent positive pressure b | <0.01 (0.02) | 0.01 (0.08) | 0.11 |
|  |  |  |  | Creatinine [Mass/volume] in Blood | <0.01 (0.02) | 0.01 (0.09) | 0.11 |
|  |  |  |  | 0.5 ML influenza A virus A/Michigan/45/2015 (H1N1) antigen 0.03 MG/ML / influenza A virus A/Singapore/INFIMH-16-0019/2016 (H3N2) antigen 0.03 MG/ML / influenza B virus B/Maryland/15/2016 antigen 0.03 MG/ML... | <0.01 (0.05) | 0.01 (0.11) | 0.11 |
|  |  |  |  | Influenza virus A H1 RNA [Presence] in Isolate by NAA with probe detection | <0.01 (0.04) | 0.01 (0.1) | 0.11 |
|  |  |  |  | Influenza virus A H3 RNA [Presence] in Isolate by NAA with probe detection | <0.01 (0.04) | 0.01 (0.1) | 0.11 |
|  |  |  |  | Chloride [Moles/volume] in Blood | <0.01 (0.02) | 0.01 (0.08) | 0.11 |
|  |  |  |  | Calcium.ionized [Moles/volume] in Blood by Ion-selective membrane electrode (ISE) | <0.01 (0.03) | 0.01 (0.09) | 0.11 |
|  |  |  |  | Activity | <0.01 (0.03) | 0.01 (0.09) | 0.11 |
|  |  |  |  | Calcium.ionized [Moles/volume] in Blood | <0.01 (0.03) | 0.01 (0.09) | 0.11 |
|  |  |  |  | Body temperature | 0.31 (0.46) | 0.36 (0.48) | 0.11 |
|  |  |  |  | Body temperature | 0.31 (0.46) | 0.36 (0.48) | 0.11 |
| ***Optum EHR*** | | | | | | | |
| Higher proportion before vaccination | | | | Higher proportion before vaccination | | | |
| index month:6 | 0.11 (0.33) | 0.02 (0.15) | -0.24 | 0.5 ML influenza A virus A/North Carolina/04/2016 (H3N2) antigen 0.03 MG/ML / influenza A virus A/Singapore/GP1908/2015 (H1N1) antigen 0.03 MG/ML... | 0.96 (0.98) | 0.05 (0.22) | -0.91 |
| index month:5 | 0.11 (0.34) | 0.03 (0.18) | -0.21 | Immunization administration (includes percutaneous, intradermal, subcutaneous, or intramuscular injections); 1 vaccine (single or combination vaccine/toxoid) | 0.29 (0.53) | 0.03 (0.17) | -0.46 |
| CHADS2VASc | 2.06 (1.61) | 1.63 (1.42) | -0.2 | Influenza, injectable, quadrivalent, preservative free | 0.25 (0.5) | 0.02 (0.13) | -0.45 |
| Charlson index - Romano adaptation | 1.83 (2.66) | 1.19 (2.2) | -0.19 | index month:10 | 0.43 (0.66) | 0.11 (0.33) | -0.44 |
| CHADS2 | 1.03 (1.28) | 0.74 (1.09) | -0.18 | Patient encounter procedure | 0.51 (0.72) | 0.16 (0.4) | -0.43 |
| Diabetes Comorbidity Severity Index (DCSI) | 1.35 (2.33) | 0.95 (2.32) | -0.12 | Administration of influenza vaccine | 0.16 (0.39) | 0.01 (0.1) | -0.35 |
| age group: 85 - 89 | 0.04 (0.19) | 0.01 (0.1) | -0.12 | Requires influenza virus vaccination | 0.12 (0.34) | 0.01 (0.11) | -0.29 |
|  |  |  |  | influenza, high dose seasonal, preservative-free | 0.08 (0.29) | <0.01 (0.07) | -0.26 |
|  |  |  |  | Requires vaccination | 0.09 (0.31) | 0.01 (0.11) | -0.25 |
|  |  |  |  | influenza, injectable, quadrivalent, contains preservative | 0.08 (0.28) | 0.01 (0.09) | -0.24 |
|  |  |  |  | 0.5 ML influenza A virus A/Idaho/07/2018 (H1N1) antigen 0.03 MG/ML / influenza A virus A/Indiana/08/2018 (H3N2) antigen 0.03 MG/ML / influenza B virus B/Iowa/06/2017 antigen 0.03 MG… | 0.06 (0.25) | <0.01 (0.04) | -0.24 |
|  |  |  |  | Vaccine Administration | 0.06 (0.25) | 0.01 (0.09) | -0.21 |
|  |  |  |  | index month:9 | 0.2 (0.45) | 0.11 (0.32) | -0.17 |
|  |  |  |  | Immunization administration through 18 years of age via any route of administration, with counseling by physician or other qualified health care professional; first or only component of each vaccine or toxoid administered | 0.05 (0.23) | 0.01 (0.11) | -0.16 |
|  |  |  |  | index month:11 | 0.19 (0.43) | 0.1 (0.32) | -0.16 |
|  |  |  |  | influenza virus vaccine, whole virus | 0.02 (0.15) | <0.01 (0) | -0.15 |
|  |  |  |  | Immunization administration (includes percutaneous, intradermal, subcutaneous, or intramuscular injections); each additional vaccine (single or combination vaccine/toxoid) for primary procedure) | 0.03 (0.18) | <0.01 (0.06) | -0.15 |
|  |  |  |  | Influenza, seasonal, injectable, preservative free | 0.03 (0.17) | <0.01 (0.06) | -0.15 |
|  |  |  |  | A detailed history; A detailed examination; Medical decision making of moderate complexity. Counseling and/o | 0.14 (0.37) | 0.08 (0.28) | -0.13 |
|  |  |  |  | Drugs Identification - Drugs Requiring Detailed Coding | 0.07 (0.27) | 0.03 (0.18) | -0.12 |
|  |  |  |  | Influenza, injectable, Madin Darby Canine Kidney, preservative free, quadrivalent | 0.02 (0.13) | <0.01 (0.02) | -0.12 |
|  |  |  |  | 0.5 ML Streptococcus pneumoniae type 1 capsular polysaccharide antigen 0.05 MG/ML / Streptococcus pneumoniae type 10A capsular polysaccharide antigen 0.05 MG/ML... | 0.02 (0.14) | <0.01 (0.05) | -0.11 |
|  |  |  |  | 0.5 ML influenza A virus A/Hong Kong/4801/2014 (H3N2) antigen 0.12 MG/ML / influenza A virus A/Michigan/45/2015 (H1N1) antigen 0.12 MG/ML / influenza B virus B/Brisbane/60/2008 antigen 0.12 MG/ML Prefilled Syringe [Fluzone 2017-2018] | 0.01 (0.11) | <0.01 (0.01) | -0.11 |
| Lower proportion before vaccination | | | | Lower proportion before vaccination | | | |
| index month:9 | 0.02 (0.14) | 0.09 (0.31) | 0.22 | Visit occurrence concept count | 0.61 (0.23) | 1.06 (0.25) | 1.31 |
| index month:10 | 0.03 (0.16) | 0.1 (0.32) | 0.22 | Any visit on day 0 | 0.58 (0.76) | 1 (1) | 0.33 |
| age group: 10-14 | <0.01 (0) | 0.04 (0.2) | 0.2 | index month:3 | 0.01 (0.08) | 0.12 (0.35) | 0.32 |
| age group: 0-4 | <0.01 (0) | 0.04 (0.2) | 0.2 | Outpatient visit on day 0 | 0.56 (0.75) | 0.89 (0.94) | 0.27 |
| age group: 5-9 | <0.01 (0) | 0.04 (0.19) | 0.19 | index month:4 | <0.01 (0.05) | 0.06 (0.25) | 0.24 |
| age group: 15-19 | <0.01 (0.07) | 0.04 (0.19) | 0.15 | index month:2 | 0.03 (0.16) | 0.1 (0.32) | 0.2 |
| index month:11 | 0.06 (0.24) | 0.11 (0.32) | 0.12 | index month:7 | <0.01 (0.03) | 0.04 (0.21) | 0.2 |
|  |  |  |  | index month:8 | 0.02 (0.14) | 0.08 (0.29) | 0.19 |
|  |  |  |  | index month:5 | <0.01 (0.02) | 0.04 (0.19) | 0.19 |
|  |  |  |  | Emergency room visit on day 0 | 0.01 (0.08) | 0.04 (0.21) | 0.17 |
|  |  |  |  | index month:1 | 0.05 (0.23) | 0.1 (0.32) | 0.13 |
|  |  |  |  | index month:6 | <0.01 (0.04) | 0.02 (0.15) | 0.13 |
|  |  |  |  | Emergency Room - General Classification | 0.01 (0.07) | 0.03 (0.17) | 0.13 |
|  |  |  |  | A detailed history; A detailed examination; and Medical decision making of moderate complexity. Counseling and/or coordination of care with o | <0.01 (0.03) | 0.01 (0.12) | 0.11 |
|  |  |  |  | An expanded problem focused history; An expanded problem focused examination; and Medical decision making of moderate complexity. Counseling | <0.01 (0.02) | 0.01 (0.11) | 0.11 |

**Table S6.** Top 50 covariates with the standardized difference of means (SDM) >0.1, covariate proportion and standard deviation, for comparison of COVID-19 vaccinated patients and unvaccinated patients indexed on (a) a date or (b) a visit, day -180 to -450.

| **COVID-19 vaccinated patients (Target) compared to unvaccinated indexed on a date (Comparator)** | | | | **COVID-19 vaccinated patients (Target) compared to unvaccinated indexed on a visit (Comparator)** | | | |
| --- | --- | --- | --- | --- | --- | --- | --- |
| **Covariate name** | **Target, mean (SD)** | **Comparator, mean (SD)** | **SDM** | **Covariate name** | **Target, mean (SD)** | **Comparator, mean (SD)** | **SDM** |
| ***CUIMC EHR*** | | | | | | | |
| Higher proportion in vaccinated patients | | | | Higher proportion in vaccinated patients | | | |
| SARS-CoV-2 (COVID-19) IgG+IgM Ab [Presence] in Serum or Plasma by Immunoassay | 0.07 (0.26) | 0.04 (0.19) | -0.16 | SARS-CoV-2 (COVID-19) IgG+IgM Ab [Presence] in Serum or Plasma by Immunoassay | 0.07 (0.26) | 0.03 (0.17) | -0.2 |
| Exposure to SARS-CoV-2 | 0.03 (0.18) | 0.01 (0.12) | -0.13 | Exposure to SARS-CoV-2 | 0.04 (0.2) | 0.02 (0.12) | -0.16 |
| Outpatient visit on day 0 | 0.02 (0.15) | 0.01 (0.1) | -0.11 | History of SARS-CoV-2 | 0.01 (0.12) | <0.01 (0.06) | -0.12 |
| Any visit on day 0 | 0.03 (0.16) | 0.01 (0.11) | -0.11 | Suspected COVID-19 | 0.03 (0.16) | 0.01 (0.11) | -0.11 |
| Suspected COVID-19 | 0.02 (0.14) | 0.01 (0.09) | -0.11 |  |  |  |  |
| Lower proportion in vaccinated patients | | | | Lower proportion in vaccinated patients | | | |
|  |  |  |  | Any visit on day 0 | 0.03 (0.16) | 1 (0) | 8.63 |
|  |  |  |  | Outpatient visit on day 0 | 0.02 (0.15) | 0.68 (0.47) | 1.88 |
|  |  |  |  | Emergency room visit on day 0 | <0.01 (0.03) | 0.12 (0.33) | 0.52 |
|  |  |  |  | Inpatient visit on day 0 | <0.01 (0.02) | 0.03 (0.17) | 0.25 |
|  |  |  |  | A detailed history; A detailed examination; Medical decision making of moderate complexity. Counseling and/o | 0.2 (0.4) | 0.28 (0.45) | 0.19 |
|  |  |  |  | Tobacco smoking behavior - finding | 0.36 (0.48) | 0.45 (0.5) | 0.18 |
|  |  |  |  | Body weight | 0.34 (0.47) | 0.43 (0.49) | 0.18 |
|  |  |  |  | Body mass index (BMI) [Ratio] | 0.33 (0.47) | 0.42 (0.49) | 0.18 |
|  |  |  |  | Systolic blood pressure | 0.38 (0.49) | 0.47 (0.5) | 0.17 |
|  |  |  |  | Diastolic blood pressure | 0.38 (0.49) | 0.47 (0.5) | 0.17 |
|  |  |  |  | Body height | 0.29 (0.45) | 0.37 (0.48) | 0.16 |
|  |  |  |  | Heart rate | 0.34 (0.48) | 0.42 (0.49) | 0.16 |
|  |  |  |  | Body weight | 0.23 (0.42) | 0.3 (0.46) | 0.15 |
|  |  |  |  | Body height | 0.2 (0.4) | 0.26 (0.44) | 0.14 |
|  |  |  |  | Heart rate | 0.24 (0.43) | 0.3 (0.46) | 0.14 |
|  |  |  |  | Systolic blood pressure | 0.26 (0.44) | 0.32 (0.47) | 0.14 |
|  |  |  |  | Diastolic blood pressure | 0.26 (0.44) | 0.32 (0.47) | 0.14 |
|  |  |  |  | Electrocardiogram, routine ECG with at least 12 leads; with interpretation and report | 0.11 (0.31) | 0.16 (0.36) | 0.13 |
|  |  |  |  | Globulin [Mass/volume] in Serum by calculation | 0.02 (0.13) | 0.04 (0.19) | 0.13 |
|  |  |  |  | A comprehensive history; A comprehensive examination; Medical decision making of high complexity. Counseling | 0.05 (0.22) | 0.08 (0.28) | 0.12 |
|  |  |  |  | Albumin/Globulin [Mass Ratio] in Serum or Plasma | 0.02 (0.15) | 0.04 (0.2) | 0.12 |
|  |  |  |  | Non-rheumatic mitral valve stenosis with regurgitation | 0.01 (0.09) | 0.02 (0.14) | 0.12 |
|  |  |  |  | Oxygen saturation in Arterial blood by Pulse oximetry | 0.23 (0.42) | 0.28 (0.45) | 0.12 |
|  |  |  |  | Body surface area | 0.08 (0.27) | 0.11 (0.31) | 0.11 |
|  |  |  |  | Oxygen saturation Calculated from oxygen partial pressure in Blood | 0.03 (0.18) | 0.06 (0.23) | 0.11 |
|  |  |  |  | Globulin [Mass/volume] in Serum by calculation | 0.01 (0.11) | 0.03 (0.16) | 0.11 |
|  |  |  |  | Atherosclerosis of coronary artery without angina pectoris | 0.03 (0.17) | 0.05 (0.22) | 0.11 |
|  |  |  |  | Echocardiography, transthoracic, real-time with image documentation (2D), includes M-mode recording, when performed, complete, with spectral Doppler echocardiography, and with color flow Doppler echocardiography | 0.04 (0.19) | 0.06 (0.23) | 0.11 |
|  |  |  |  | Albumin/Globulin [Mass Ratio] in Serum or Plasma | 0.01 (0.11) | 0.03 (0.16) | 0.11 |
| ***Optum EHR*** | | | | | | | |
| Higher proportion in vaccinated patients | | | | Higher proportion in vaccinated patients | | | |
| Outpatient visit on day 0 | 0.72 (0.85) | 0.13 (0.36) | -0.64 | Basophils/100 leukocytes in Blood | 1.63 (1.28) | 1.14 (1.07) | -0.3 |
| Any visit on day 0 | 0.73 (0.85) | 0.15 (0.38) | -0.62 | visit_occurrence concept count | 17.33 (19.33) | 10.54 (13.63) | -0.29 |
| Basophils/100 leukocytes in Blood | 1.63 (1.28) | 1.24 (1.11) | -0.23 | Eosinophils/100 leukocytes in Blood | 1.51 (1.23) | 1.06 (1.03) | -0.28 |
| Albumin [Presence] in Urine | 0.9 (0.95) | 0.62 (0.79) | -0.22 | Albumin [Presence] in Urine | 0.9 (0.95) | 0.56 (0.75) | -0.28 |
| Eosinophils/100 leukocytes in Blood | 1.51 (1.23) | 1.16 (1.08) | -0.21 | Calcium.ionized/Calcium.total corrected for albumin in Blood | 1.32 (1.15) | 0.91 (0.96) | -0.28 |
| Albumin/Protein.total in Serum or Plasma | 0.87 (0.93) | 0.62 (0.79) | -0.2 | Albumin/Protein.total in Serum or Plasma | 0.87 (0.93) | 0.56 (0.75) | -0.25 |
| 0.5 ML influenza A virus A/North Carolina/04/2016 (H3N2) antigen 0.03 MG/ML / influenza A virus A/Singapore/GP1908/2015 (H1N1) antigen 0.03 MG/ML / influenza B virus B/Iowa/06/2017... | 0.2 (0.45) | 0.09 (0.3) | -0.2 | Monocytes/100 leukocytes in Blood | 1.32 (1.15) | 0.95 (0.97) | -0.25 |
| Alkaline phosphatase.renal/Alkaline phosphatase.total in Serum or Plasma | 0.86 (0.93) | 0.63 (0.79) | -0.19 | Alkaline phosphatase.renal/Alkaline phosphatase.total in Serum or Plasma | 0.86 (0.93) | 0.57 (0.76) | -0.24 |
| Calcium.ionized/Calcium.total corrected for albumin in Blood | 1.32 (1.15) | 1.03 (1.02) | -0.19 | Sodium [Moles/volume] in Saliva (oral fluid) | 1.31 (1.15) | 0.95 (0.98) | -0.24 |
| Globulin [Mass/volume] in Serum | 0.36 (0.6) | 0.22 (0.47) | -0.19 | Penicillin G potassium [Mass] of Dose | 1.41 (1.19) | 1.04 (1.02) | -0.24 |
| Monocytes/100 leukocytes in Blood | 1.32 (1.15) | 1.03 (1.02) | -0.19 | Chloride [Moles/volume] in Saliva (oral fluid) | 1.26 (1.12) | 0.91 (0.96) | -0.23 |
| Lymphocytes/100 leukocytes in Blood | 1.16 (1.08) | 0.91 (0.96) | -0.17 | Lymphocytes/100 leukocytes in Blood | 1.16 (1.08) | 0.84 (0.92) | -0.23 |
| Laboratory - Bacteriology and Microbiology | 0.26 (0.51) | 0.15 (0.39) | -0.17 | Lymphocytes/100 leukocytes in Blood | 0.59 (0.77) | 0.38 (0.61) | -0.22 |
| Neutrophil cytoplasmic Ab.perinuclear [Presence] in Serum | 0.5 (0.71) | 0.34 (0.59) | -0.17 | Carbon dioxide [Partial pressure] in Blood | 1.17 (1.08) | 0.86 (0.93) | -0.22 |
| Exposure to viral disease | 0.21 (0.46) | 0.12 (0.35) | -0.16 | 0.5 ML influenza A virus A/North Carolina/04/2016 (H3N2) antigen 0.03 MG/ML / influenza A virus A/Singapore/GP1908/2015 (H1N1) antigen 0.03 MG/ML / influenza B virus B/Iowa/06... | 0.2 (0.45) | 0.09 (0.3) | -0.21 |
| Triglyceride [Percentile] | 0.24 (0.49) | 0.14 (0.37) | -0.16 | Aspartate aminotransferase [Presence] in Body fluid | 0.72 (0.85) | 0.49 (0.7) | -0.21 |
| visit_occurrence concept count | 17.33 (19.33) | 13.35 (16.98) | -0.15 | Leukocytes [#/volume] in Blood | 1.13 (1.07) | 0.85 (0.92) | -0.2 |
| Aspartate aminotransferase [Presence] in Body fluid | 0.72 (0.85) | 0.55 (0.74) | -0.15 | Bilirubin.total [Presence] in Urine | 0.63 (0.8) | 0.43 (0.66) | -0.2 |
| Lymphocytes/100 leukocytes in Blood | 0.59 (0.77) | 0.44 (0.66) | -0.15 | Laboratory - Bacteriology and Microbiology | 0.26 (0.51) | 0.14 (0.37) | -0.19 |
| Chloride [Moles/volume] in Saliva (oral fluid) | 1.26 (1.12) | 1.04 (1.02) | -0.15 | Neutrophil Ab [Presence] in Serum | 0.77 (0.87) | 0.55 (0.74) | -0.19 |
| Sodium [Moles/volume] in Saliva (oral fluid) | 1.31 (1.15) | 1.09 (1.04) | -0.14 | Anion gap in Blood | 0.83 (0.91) | 0.61 (0.78) | -0.19 |
| Cholesterol.total/Cholesterol in HDL [Percentile] | 0.25 (0.5) | 0.16 (0.4) | -0.14 | Platelets [#/volume] in Blood | 1.12 (1.06) | 0.86 (0.93) | -0.19 |
| Infectious agent detection by nucleic acid (DNA or RNA); severe acute respiratory syndrome coronavirus 2 (SARS-CoV-2) (Coronavirus disease [COVID-19]), amplified probe technique | 0.12 (0.35) | 0.06 (0.25) | -0.14 | Glucose [Mass/volume] in Serum or Plasma | 0.92 (0.96) | 0.68 (0.82) | -0.19 |
| Carbon dioxide [Partial pressure] in Blood | 1.17 (1.08) | 0.97 (0.99) | -0.14 | MCHC [Mass/volume] | 1.07 (1.03) | 0.81 (0.9) | -0.19 |
| Penicillin G potassium [Mass] of Dose | 1.41 (1.19) | 1.2 (1.09) | -0.13 | MCH [Entitic mass] | 1.11 (1.05) | 0.85 (0.92) | -0.18 |
| Leukocytes [#/volume] in Blood | 1.13 (1.07) | 0.95 (0.97) | -0.13 | Neutrophil cytoplasmic Ab.perinuclear [Presence] in Serum | 0.5 (0.71) | 0.34 (0.58) | -0.18 |
| Hemoglobin [Mass/volume] in Blood | 0.76 (0.87) | 0.61 (0.78) | -0.13 | Globulin [Mass/volume] in Serum | 0.36 (0.6) | 0.23 (0.48) | -0.17 |
| Hematocrit [Volume Fraction] of Blood | 0.78 (0.88) | 0.63 (0.79) | -0.13 | MCV [Entitic volume] | 1.07 (1.03) | 0.84 (0.91) | -0.17 |
| Erythrocytes [#/volume] in Blood | 0.86 (0.93) | 0.71 (0.84) | -0.12 | Alanine aminotransferase [Enzymatic activity/volume] in Serum or Plasma | 0.63 (0.8) | 0.46 (0.68) | -0.17 |
| Neutrophil Ab [Presence] in Serum | 0.77 (0.87) | 0.62 (0.79) | -0.12 | Laboratory - Chemistry | 0.31 (0.55) | 0.19 (0.44) | -0.17 |
| Eosinophils/100 leukocytes in Blood | 0.15 (0.39) | 0.09 (0.3) | -0.12 | Hematocrit [Volume Fraction] of Blood | 0.78 (0.88) | 0.58 (0.76) | -0.17 |
| Alanine aminotransferase [Enzymatic activity/volume] in Serum or Plasma | 0.63 (0.8) | 0.5 (0.71) | -0.12 | Cotinine/Creatinine [Mass Ratio] in Urine | 0.93 (0.97) | 0.72 (0.85) | -0.16 |
| Laboratory - Chemistry | 0.31 (0.55) | 0.22 (0.47) | -0.12 | Hemoglobin [Mass/volume] in Blood | 0.76 (0.87) | 0.57 (0.76) | -0.16 |
| General finding of observation of patient | 0.07 (0.26) | 0.03 (0.17) | -0.12 | Erythrocytes [#/volume] in Blood | 0.86 (0.93) | 0.66 (0.82) | -0.16 |
| Cholesterol in LDL [Percentile] | 0.37 (0.6) | 0.27 (0.52) | -0.12 | Prothrombin time (PT) | 0.29 (0.54) | 0.18 (0.42) | -0.16 |
| Neutrophil Ab [Presence] in Serum | 0.13 (0.37) | 0.08 (0.28) | -0.12 | Monocytes/100 leukocytes in Blood | 0.29 (0.54) | 0.18 (0.43) | -0.16 |
| Other Imaging Services - Screening Mammography | 0.08 (0.28) | 0.04 (0.19) | -0.12 | Erythrocytes [#/volume] in Blood | 0.59 (0.76) | 0.43 (0.65) | -0.16 |
| Cholesterol esters/Cholesterol.total in Serum or Plasma | 0.36 (0.6) | 0.27 (0.52) | -0.12 | Laboratory - General Classification | 0.31 (0.55) | 0.2 (0.45) | -0.15 |
| Cholesterol.total/Cholesterol in HDL [Percentile] | 0.36 (0.6) | 0.27 (0.52) | -0.12 | MCV [Entitic volume] | 0.21 (0.46) | 0.12 (0.35) | -0.15 |
| Cholesterol esters/Cholesterol.total in Serum or Plasma | 0.18 (0.43) | 0.12 (0.35) | -0.12 | Hemoglobin [Mass/volume] in Blood | 0.69 (0.83) | 0.52 (0.72) | -0.15 |
| Patient encounter procedure | 0.64 (0.8) | 0.51 (0.72) | -0.12 | Neutrophil Ab [Presence] in Serum | 0.13 (0.37) | 0.07 (0.26) | -0.15 |
| Triglyceride [Percentile] | 0.36 (0.6) | 0.27 (0.52) | -0.12 | Platelet mean volume [Entitic volume] in Blood | 0.82 (0.91) | 0.64 (0.8) | -0.15 |
| Bilirubin.total [Presence] in Urine | 0.63 (0.8) | 0.51 (0.72) | -0.11 | Exposure to viral disease | 0.21 (0.46) | 0.13 (0.36) | -0.15 |
| Glucose [Mass/volume] in Serum or Plasma | 0.92 (0.96) | 0.77 (0.88) | -0.11 | Platelet distribution width [Ratio] in Blood | 0.81 (0.9) | 0.64 (0.8) | -0.15 |
| Monocytes/100 leukocytes in Blood | 0.14 (0.37) | 0.09 (0.29) | -0.11 | Collection of venous blood by venipuncture | 0.48 (0.7) | 0.35 (0.59) | -0.14 |
| Monocytes/100 leukocytes in Blood | 0.29 (0.54) | 0.21 (0.46) | -0.11 | Eosinophils/100 leukocytes in Blood | 0.15 (0.39) | 0.08 (0.29) | -0.14 |
| Platelets [#/volume] in Blood | 1.12 (1.06) | 0.96 (0.98) | -0.11 | Monocytes/100 leukocytes in Blood | 0.14 (0.37) | 0.07 (0.27) | -0.14 |
| SARS-CoV-2 (COVID-19) RNA [Presence] in Respiratory specimen by NAA with probe detection | 0.28 (0.53) | 0.21 (0.46) | -0.11 | Laboratory - Hematology | 0.25 (0.5) | 0.16 (0.4) | -0.14 |
| Cotinine/Creatinine [Mass Ratio] in Urine | 0.93 (0.97) | 0.79 (0.89) | -0.11 | Triglyceride [Percentile] | 0.24 (0.49) | 0.15 (0.39) | -0.13 |
| Lower proportion in vaccinated patients | | | | Lower proportion in vaccinated patients | | | |
| Oxygen [Partial pressure] in Blood | 0.12 (0.34) | 0.31 (0.55) | 0.29 | Oxygen [Partial pressure] in Blood | 0.12 (0.34) | 0.34 (0.58) | 0.33 |
|  |  |  |  | Emergency room visit on day 0 | <0.01 (0.03) | 0.06 (0.24) | 0.23 |
|  |  |  |  | Any visit on day 0 | 0.73 (0.85) | 1 (1) | 0.21 |
|  |  |  |  | Inpatient visit on day 0 | <0.01 (0.03) | 0.03 (0.16) | 0.15 |

**Table S7.** Top 50 covariates with the standardized difference of means (SDM) >0.1, covariate proportion and standard deviation, for comparison of influenza vaccinated patients and unvaccinated patients indexed on (a) a date or (b) a visit, day -180 to -450.

| **Influenza vaccinated patients (Target) compared to unvaccinated indexed on a date (Comparator)** | | | | **Influenza vaccinated patients (Target) compared to unvaccinated indexed on a visit (Comparator)** | | | |
| --- | --- | --- | --- | --- | --- | --- | --- |
| **Covariate name** | **Target, mean (SD)** | **Comparator, mean (SD)** | **SDM** | **Covariate name** | **Target, mean (SD)** | **Comparator, mean (SD)** | **SDM** |
| ***CUIMC EHR*** | | | | | | | |
| Higher proportion in vaccinated patients | | | | Higher proportion in vaccinated patients | | | |
| Any visit on day 0 | 0.55 (0.5) | 0.01 (0.07) | -1.53 | Tobacco smoking behavior - finding | 0.58 (0.49) | 0.25 (0.43) | -0.7 |
| Outpatient visit on day 0 | 0.55 (0.5) | <0.01 (0.07) | -1.53 | Body weight | 0.72 (0.45) | 0.43 (0.5) | -0.6 |
| Body weight | 0.75 (0.43) | 0.31 (0.46) | -0.99 | Body mass index (BMI) [Ratio] | 0.69 (0.46) | 0.41 (0.49) | -0.58 |
| Heart rate | 0.75 (0.43) | 0.32 (0.47) | -0.95 | Body temperature | 0.56 (0.5) | 0.31 (0.46) | -0.52 |
| Diastolic blood pressure | 0.77 (0.42) | 0.35 (0.48) | -0.94 | Patient encounter status | 0.16 (0.36) | 0.02 (0.14) | -0.51 |
| Systolic blood pressure | 0.77 (0.42) | 0.35 (0.48) | -0.94 | Heart rate | 0.69 (0.46) | 0.45 (0.5) | -0.5 |
| Body mass index (BMI) [Ratio] | 0.72 (0.45) | 0.3 (0.46) | -0.93 | Body height Measured | 0.36 (0.48) | 0.15 (0.36) | -0.48 |
| Body height Measured | 0.36 (0.48) | 0.05 (0.21) | -0.85 | Systolic blood pressure | 0.71 (0.45) | 0.48 (0.5) | -0.48 |
| Body temperature | 0.6 (0.49) | 0.21 (0.41) | -0.84 | Diastolic blood pressure | 0.71 (0.45) | 0.48 (0.5) | -0.48 |
| Tobacco smoking behavior - finding | 0.65 (0.48) | 0.27 (0.44) | -0.82 | Influenza, injectable, quadrivalent, preservative free | 0.17 (0.38) | 0.03 (0.17) | -0.47 |
| Patient encounter procedure | 0.31 (0.46) | 0.04 (0.2) | -0.75 | Hematocrit [Volume Fraction] of Blood by Automated count | 0.42 (0.49) | 0.2 (0.4) | -0.47 |
| Platelet mean volume [Entitic volume] in Blood | 0.32 (0.47) | 0.06 (0.24) | -0.69 | MCHC [Mass/volume] by Automated count | 0.41 (0.49) | 0.2 (0.4) | -0.47 |
| Glomerular filtration rate/1.73 sq M.predicted among blacks [Volume Rate/Area] in Serum, Plasma or Blood by Creatinine-based formula (MDRD) | 0.29 (0.45) | 0.05 (0.22) | -0.69 | Leukocytes [#/volume] in Blood by Automated count | 0.41 (0.49) | 0.2 (0.4) | -0.47 |
| Nucleated erythrocytes/100 leukocytes [Ratio] in Blood | 0.31 (0.46) | 0.06 (0.23) | -0.68 | MCH [Entitic mass] by Automated count | 0.41 (0.49) | 0.2 (0.4) | -0.47 |
| Nucleated erythrocytes [#/volume] in Blood | 0.31 (0.46) | 0.06 (0.23) | -0.68 | Erythrocyte distribution width [Ratio] by Automated count | 0.41 (0.49) | 0.2 (0.4) | -0.47 |
| Glomerular filtration rate/1.73 sq M.predicted among non-blacks [Volume Rate/Area] in Serum, Plasma or Blood by Creatinine-based formula (MDRD) | 0.29 (0.45) | 0.05 (0.21) | -0.68 | Erythrocytes [#/volume] in Blood by Automated count | 0.41 (0.49) | 0.2 (0.4) | -0.47 |
| Platelets [#/volume] in Blood | 0.32 (0.47) | 0.07 (0.25) | -0.68 | MCV [Entitic volume] by Automated count | 0.41 (0.49) | 0.2 (0.4) | -0.47 |
| Nucleated erythrocytes/100 leukocytes [Ratio] in Blood by Automated count | 0.31 (0.46) | 0.06 (0.24) | -0.68 | Hemoglobin [Mass/volume] in Blood | 0.42 (0.49) | 0.21 (0.41) | -0.46 |
| Nucleated erythrocytes [#/volume] in Blood by Automated count | 0.31 (0.46) | 0.06 (0.24) | -0.68 | Cholesterol [Mass/volume] in Serum or Plasma | 0.24 (0.43) | 0.08 (0.27) | -0.45 |
| Leukocytes [#/volume] in Blood by Automated count | 0.5 (0.5) | 0.2 (0.4) | -0.66 | Cholesterol in HDL [Mass/volume] in Serum or Plasma | 0.24 (0.43) | 0.08 (0.27) | -0.45 |
| MCHC [Mass/volume] by Automated count | 0.5 (0.5) | 0.2 (0.4) | -0.66 | Cholesterol in LDL [Mass/volume] in Serum or Plasma by calculation | 0.24 (0.42) | 0.08 (0.27) | -0.45 |
| MCH [Entitic mass] by Automated count | 0.5 (0.5) | 0.2 (0.4) | -0.66 | Triglyceride [Mass/volume] in Serum or Plasma | 0.24 (0.43) | 0.08 (0.27) | -0.45 |
| Erythrocyte distribution width [Ratio] by Automated count | 0.5 (0.5) | 0.2 (0.4) | -0.66 | Cholesterol.total/Cholesterol in HDL [Mass Ratio] in Serum or Plasma | 0.23 (0.42) | 0.08 (0.27) | -0.44 |
| Erythrocytes [#/volume] in Blood by Automated count | 0.5 (0.5) | 0.2 (0.4) | -0.66 | Urea nitrogen [Mass/volume] in Serum or Plasma | 0.38 (0.49) | 0.19 (0.39) | -0.44 |
| MCV [Entitic volume] by Automated count | 0.5 (0.5) | 0.2 (0.4) | -0.66 | Creatinine [Mass/volume] in Serum or Plasma | 0.38 (0.49) | 0.19 (0.39) | -0.44 |
| Hematocrit [Volume Fraction] of Blood by Automated count | 0.5 (0.5) | 0.2 (0.4) | -0.66 | Calcium [Mass/volume] in Serum or Plasma | 0.38 (0.49) | 0.19 (0.39) | -0.44 |
| Hemoglobin [Mass/volume] in Blood | 0.5 (0.5) | 0.2 (0.4) | -0.66 | Potassium [Moles/volume] in Serum or Plasma | 0.38 (0.49) | 0.19 (0.39) | -0.44 |
| Chloride [Moles/volume] in Serum or Plasma | 0.5 (0.5) | 0.2 (0.4) | -0.66 | Chloride [Moles/volume] in Serum or Plasma | 0.38 (0.49) | 0.19 (0.39) | -0.44 |
| Carbon dioxide, total [Moles/volume] in Serum or Plasma | 0.5 (0.5) | 0.2 (0.4) | -0.66 | Sodium [Moles/volume] in Serum or Plasma | 0.38 (0.49) | 0.19 (0.39) | -0.44 |
| Sodium [Moles/volume] in Serum or Plasma | 0.5 (0.5) | 0.2 (0.4) | -0.66 | Carbon dioxide, total [Moles/volume] in Serum or Plasma | 0.38 (0.49) | 0.19 (0.39) | -0.44 |
| Potassium [Moles/volume] in Serum or Plasma | 0.5 (0.5) | 0.2 (0.4) | -0.66 | Vaccination needed | 0.09 (0.29) | <0.01 (0.06) | -0.42 |
| Calcium [Mass/volume] in Serum or Plasma | 0.5 (0.5) | 0.2 (0.4) | -0.66 | Body temperature | 0.24 (0.42) | 0.08 (0.28) | -0.42 |
| Creatinine [Mass/volume] in Serum or Plasma | 0.5 (0.5) | 0.2 (0.4) | -0.66 | Patient encounter procedure | 0.32 (0.47) | 0.15 (0.36) | -0.41 |
| Urea nitrogen [Mass/volume] in Serum or Plasma | 0.5 (0.5) | 0.2 (0.4) | -0.66 | Platelets [#/volume] in Blood by Automated count | 0.15 (0.36) | 0.03 (0.18) | -0.41 |
| Anion gap in Serum or Plasma | 0.48 (0.5) | 0.19 (0.39) | -0.64 | Anion gap in Serum or Plasma | 0.36 (0.48) | 0.18 (0.39) | -0.41 |
| Hematocrit [Volume Fraction] of Blood | 0.34 (0.47) | 0.09 (0.29) | -0.64 | Influenza, seasonal, injectable | 0.12 (0.33) | 0.02 (0.14) | -0.41 |
| Nucleated erythrocytes/100 leukocytes [Ratio] in Blood | 0.25 (0.43) | 0.04 (0.2) | -0.61 | Alanine aminotransferase [Enzymatic activity/volume] in Serum or Plasma | 0.33 (0.47) | 0.16 (0.37) | -0.41 |
| Nucleated erythrocytes/100 leukocytes [Ratio] in Blood by Automated count | 0.25 (0.43) | 0.05 (0.21) | -0.61 | Leukocytes [#/volume] in Blood by Automated count | 0.29 (0.45) | 0.13 (0.34) | -0.4 |
| Respiratory rate | 0.46 (0.5) | 0.19 (0.39) | -0.6 | Aspartate aminotransferase [Enzymatic activity/volume] in Serum or Plasma | 0.32 (0.47) | 0.16 (0.36) | -0.4 |
| Glucose [Mass/volume] in Serum or Plasma | 0.37 (0.48) | 0.12 (0.33) | -0.6 | Alkaline phosphatase [Enzymatic activity/volume] in Serum or Plasma | 0.32 (0.47) | 0.16 (0.36) | -0.4 |
| Platelet mean volume [Entitic volume] in Blood | 0.24 (0.43) | 0.04 (0.2) | -0.6 | Albumin [Mass/volume] in Serum or Plasma | 0.32 (0.47) | 0.16 (0.36) | -0.39 |
| Alanine aminotransferase [Enzymatic activity/volume] in Serum or Plasma | 0.44 (0.5) | 0.18 (0.38) | -0.58 | Protein [Mass/volume] in Serum or Plasma | 0.32 (0.47) | 0.16 (0.36) | -0.39 |
| Calcium [Mass/volume] in Serum or Plasma | 0.39 (0.49) | 0.15 (0.36) | -0.57 | Bilirubin.total [Mass/volume] in Serum or Plasma | 0.32 (0.47) | 0.16 (0.37) | -0.39 |
| Potassium [Moles/volume] in Serum or Plasma | 0.4 (0.49) | 0.15 (0.36) | -0.57 | Follow-up encounter | 0.15 (0.36) | 0.04 (0.19) | -0.39 |
| Albumin [Mass/volume] in Serum or Plasma | 0.43 (0.5) | 0.18 (0.38) | -0.57 | Calcium [Mass/volume] in Serum or Plasma | 0.29 (0.46) | 0.14 (0.34) | -0.39 |
| Aspartate aminotransferase [Enzymatic activity/volume] in Serum or Plasma | 0.43 (0.49) | 0.18 (0.38) | -0.57 | Sodium [Moles/volume] in Serum or Plasma | 0.29 (0.45) | 0.14 (0.34) | -0.39 |
| Hemoglobin A1c/Hemoglobin.total in Blood | 0.21 (0.41) | 0.04 (0.18) | -0.57 | Potassium [Moles/volume] in Serum or Plasma | 0.3 (0.46) | 0.14 (0.35) | -0.39 |
| Sodium [Moles/volume] in Serum or Plasma | 0.39 (0.49) | 0.15 (0.36) | -0.56 | Platelet mean volume [Entitic volume] in Blood by Automated count | 0.13 (0.34) | 0.03 (0.17) | -0.38 |
| Bilirubin.total [Mass/volume] in Serum or Plasma | 0.43 (0.49) | 0.18 (0.38) | -0.56 | MCHC [Mass/volume] by Automated count | 0.28 (0.45) | 0.13 (0.34) | -0.38 |
| Essential hypertension | 0.27 (0.44) | 0.08 (0.26) | -0.53 | Carbon dioxide, total [Moles/volume] in Serum or Plasma | 0.28 (0.45) | 0.13 (0.34) | -0.38 |
| Lower proportion in vaccinated patients | | | | Lower proportion in vaccinated patients | | | |
| race = White | 0.41 (0.49) | 0.48 (0.5) | 0.14 | Any visit on day 0 | 0.56 (0.5) | 1 (0) | 1.26 |
|  |  |  |  | Emergency room visit on day 0 | <0.01 (0.04) | 0.2 (0.4) | 0.71 |
|  |  |  |  | Outpatient visit on day 0 | 0.56 (0.5) | 0.8 (0.4) | 0.53 |
|  |  |  |  | Inpatient visit on day 0 | <0.01 (0.03) | 0.01 (0.12) | 0.16 |
| ***Optum EHR*** | | | | | | | |
| Higher proportion in vaccinated patients | | | | Higher proportion in vaccinated patients | | | |
| Any visit on day 0 | 0.53 (0.73) | 0.03 (0.16) | -0.68 | 0.5 ML influenza A virus A/North Carolina/04/2016 (H3N2) antigen 0.03 MG/ML / influenza A virus A/Singapore/GP1908/2015 (H1N1) antigen 0.03 MG/ML / influenza B virus B/Iowa/06/... | 0.57 (0.75) | 0.11 (0.33) | -0.55 |
| Outpatient visit on day 0 | 0.51 (0.72) | 0.02 (0.15) | -0.67 | visit_occurrence concept count during day -450 through -181 concept_count relative to index | 12.35 (15.08) | 7.37 (9.96) | -0.28 |
| 0.5 ML influenza A virus A/North Carolina/04/2016 (H3N2) antigen 0.03 MG/ML / influenza A virus A/Singapore/GP1908/2015 (H1N1) antigen 0.03 MG/ML / influenza B virus B/Iowa/06/2017 antigen 0.03 MG/ML... | 0.57 (0.75) | 0.09 (0.3) | -0.59 | Influenza, injectable, quadrivalent, preservative free | 0.12 (0.34) | 0.02 (0.16) | -0.24 |
| Visit occurrence concept count during day -450 through -181 concept_count relative to index | 12.41 (15.14) | 5.79 (10.89) | -0.35 | Immunization administration (includes percutaneous, intradermal, subcutaneous, or intramuscular injections) | 0.18 (0.42) | 0.06 (0.24) | -0.24 |
| Patient encounter procedure | 0.58 (0.76) | 0.28 (0.53) | -0.32 | Requires influenza virus vaccination | 0.09 (0.3) | 0.02 (0.12) | -0.23 |
| Calcium.ionized/Calcium.total corrected for albumin in Blood | 0.78 (0.88) | 0.44 (0.66) | -0.31 | Administration of influenza vaccine | 0.09 (0.3) | 0.02 (0.13) | -0.22 |
| Carbon dioxide [Partial pressure] in Blood | 0.73 (0.85) | 0.41 (0.64) | -0.3 | Calcium.ionized/Calcium.total corrected for albumin in Blood | 0.78 (0.88) | 0.54 (0.73) | -0.21 |
| Penicillin G potassium [Mass] of Dose | 0.85 (0.92) | 0.51 (0.71) | -0.29 | Glucose [Mass/volume] in Serum or Plasma | 0.86 (0.92) | 0.6 (0.77) | -0.21 |
| Chloride [Moles/volume] in Saliva (oral fluid) | 0.76 (0.87) | 0.44 (0.67) | -0.29 | Penicillin G potassium [Mass] of Dose | 0.85 (0.92) | 0.6 (0.77) | -0.21 |
| Sodium [Moles/volume] in Saliva (oral fluid) | 0.82 (0.91) | 0.49 (0.7) | -0.29 | Carbon dioxide [Partial pressure] in Blood | 0.73 (0.85) | 0.5 (0.71) | -0.21 |
| Immunization administration (includes percutaneous, intradermal, subcutaneous, or intramuscular injections) | 0.18 (0.42) | 0.04 (0.21) | -0.28 | Sodium [Moles/volume] in Saliva (oral fluid) | 0.82 (0.9) | 0.58 (0.76) | -0.21 |
| Albumin [Presence] in Urine | 0.5 (0.71) | 0.27 (0.52) | -0.27 | Patient encounter procedure | 0.57 (0.76) | 0.38 (0.61) | -0.2 |
| A detailed history; A detailed examination; Medical decision making of moderate complexity. Counseling and/o | 0.39 (0.62) | 0.18 (0.43) | -0.27 | Chloride [Moles/volume] in Saliva (oral fluid) | 0.76 (0.87) | 0.53 (0.73) | -0.2 |
| Cholesterol in LDL [Percentile] | 0.32 (0.56) | 0.14 (0.37) | -0.27 | Albumin [Presence] in Urine | 0.5 (0.71) | 0.34 (0.58) | -0.18 |
| Cotinine/Creatinine [Mass Ratio] in Urine | 0.65 (0.81) | 0.38 (0.62) | -0.26 | Cotinine/Creatinine [Mass Ratio] in Urine | 0.65 (0.81) | 0.46 (0.68) | -0.18 |
| Cholesterol esters/Cholesterol.total in Serum or Plasma | 0.32 (0.56) | 0.14 (0.37) | -0.26 | Albumin/Protein.total in Serum or Plasma | 0.5 (0.71) | 0.33 (0.58) | -0.18 |
| Cholesterol.total/Cholesterol in HDL [Percentile] | 0.32 (0.56) | 0.14 (0.37) | -0.26 | MCHC [Mass/volume] | 0.68 (0.82) | 0.49 (0.7) | -0.17 |
| An expanded problem focused history; An expanded problem focused examination; | 0.44 (0.67) | 0.23 (0.48) | -0.26 | Glucose [Mass/volume] in Serum or Plasma | 0.57 (0.76) | 0.4 (0.64) | -0.17 |
| Triglyceride [Percentile] | 0.32 (0.56) | 0.14 (0.37) | -0.26 | Cholesterol in LDL [Percentile] | 0.32 (0.56) | 0.2 (0.44) | -0.17 |
| Albumin/Protein.total in Serum or Plasma | 0.5 (0.7) | 0.27 (0.52) | -0.26 | MCV [Entitic volume] | 0.65 (0.81) | 0.48 (0.69) | -0.17 |
| Influenza, injectable, quadrivalent, preservative free | 0.11 (0.34) | 0.02 (0.14) | -0.26 | Requires vaccination | 0.08 (0.28) | 0.02 (0.16) | -0.17 |
| Glucose [Mass/volume] in Serum or Plasma | 0.86 (0.93) | 0.55 (0.74) | -0.26 | Cholesterol esters/Cholesterol.total in Serum or Plasma | 0.32 (0.56) | 0.2 (0.44) | -0.17 |
| Collection of venous blood by venipuncture | 0.37 (0.61) | 0.18 (0.43) | -0.25 | influenza, high dose seasonal, preservative-free | 0.05 (0.21) | 0.01 (0.09) | -0.17 |
| Leukocytes [#/volume] in Blood | 0.67 (0.82) | 0.41 (0.64) | -0.25 | Cholesterol.total/Cholesterol in HDL [Percentile] | 0.32 (0.56) | 0.2 (0.44) | -0.17 |
| Alkaline phosphatase.renal/Alkaline phosphatase.total in Serum or Plasma | 0.48 (0.69) | 0.27 (0.52) | -0.25 | Triglyceride [Percentile] | 0.32 (0.56) | 0.2 (0.44) | -0.17 |
| MCHC [Mass/volume] | 0.68 (0.82) | 0.42 (0.64) | -0.25 | Leukocytes [#/volume] in Blood | 0.67 (0.82) | 0.49 (0.7) | -0.17 |
| Requires influenza virus vaccination | 0.09 (0.3) | 0.01 (0.1) | -0.25 | Alkaline phosphatase.renal/Alkaline phosphatase.total in Serum or Plasma | 0.48 (0.69) | 0.33 (0.58) | -0.17 |
| Body mass index (BMI) [Ratio] | 0.72 (0.85) | 0.45 (0.67) | -0.25 | A detailed history; A detailed examination; Medical decision making of moderate complexity. Counseling and/o | 0.39 (0.62) | 0.25 (0.5) | -0.16 |
| Platelets [#/volume] in Blood | 0.67 (0.82) | 0.41 (0.64) | -0.25 | Cholesterol, serum, total (82465) Lipoprotein, direct measurement, high density cholesterol (HDL cholesterol) (83718) Triglycerides (84478) | 0.21 (0.45) | 0.11 (0.34) | -0.16 |
| Administration of influenza vaccine | 0.09 (0.3) | 0.01 (0.11) | -0.24 | Platelets [#/volume] in Blood | 0.67 (0.82) | 0.49 (0.7) | -0.16 |
| MCV [Entitic volume] | 0.65 (0.81) | 0.4 (0.64) | -0.24 | 0.5 ML influenza A virus A/Idaho/07/2018 (H1N1) antigen 0.03 MG/ML / influenza A virus A/Indiana/08/2018 (H3N2) antigen 0.03 MG/ML / influenza B virus B/Iowa/06/2017 antigen 0.03 MG/ML… | 0.04 (0.21) | 0.01 (0.08) | -0.16 |
| Lymphocytes/100 leukocytes in Blood | 0.62 (0.79) | 0.38 (0.62) | -0.24 | Monocytes/100 leukocytes in Blood | 0.69 (0.83) | 0.51 (0.72) | -0.16 |
| Cholesterol, serum, total (82465) Lipoprotein, direct measurement, high density cholesterol (HDL cholesterol) (83718) Triglycerides (84478) | 0.21 (0.46) | 0.08 (0.28) | -0.24 | Basophils/100 leukocytes in Blood | 0.82 (0.91) | 0.63 (0.79) | -0.16 |
| Erythrocytes [#/volume] in Blood | 0.56 (0.75) | 0.33 (0.58) | -0.24 | influenza, injectable, quadrivalent, contains preservative | 0.05 (0.22) | 0.01 (0.1) | -0.16 |
| Body height | 0.68 (0.82) | 0.43 (0.65) | -0.24 | Lymphocytes/100 leukocytes in Blood | 0.62 (0.79) | 0.46 (0.68) | -0.16 |
| Bilirubin.total [Presence] in Urine | 0.46 (0.68) | 0.26 (0.51) | -0.24 | Collection of venous blood by venipuncture | 0.37 (0.61) | 0.25 (0.5) | -0.16 |
| Body weight | 0.72 (0.85) | 0.46 (0.68) | -0.24 | Bilirubin.total [Presence] in Urine | 0.46 (0.68) | 0.32 (0.57) | -0.16 |
| Monocytes/100 leukocytes in Blood | 0.69 (0.83) | 0.44 (0.66) | -0.24 | MCH [Entitic mass] | 0.64 (0.8) | 0.48 (0.69) | -0.16 |
| Pulse intensity of Unspecified artery palpation | 0.65 (0.81) | 0.41 (0.64) | -0.23 | Erythrocytes [#/volume] in Blood | 0.56 (0.75) | 0.41 (0.64) | -0.15 |
| Systolic blood pressure | 0.7 (0.84) | 0.45 (0.67) | -0.23 | Aspartate aminotransferase [Presence] in Body fluid | 0.43 (0.65) | 0.3 (0.55) | -0.15 |
| MCH [Entitic mass] | 0.64 (0.8) | 0.41 (0.64) | -0.23 | Eosinophils/100 leukocytes in Blood | 0.76 (0.87) | 0.59 (0.77) | -0.15 |
| Glucose [Mass/volume] in Serum or Plasma | 0.45 (0.67) | 0.25 (0.5) | -0.23 | Neutrophil Ab [Presence] in Serum | 0.4 (0.63) | 0.28 (0.53) | -0.15 |
| Diastolic blood pressure | 0.7 (0.84) | 0.45 (0.67) | -0.23 | Anion gap in Blood | 0.52 (0.72) | 0.38 (0.62) | -0.15 |
| Basophils/100 leukocytes in Blood | 0.82 (0.91) | 0.55 (0.74) | -0.23 | Hemoglobin; glycosylated (A1C) | 0.13 (0.37) | 0.07 (0.26) | -0.14 |
| Glucose [Mass/volume] in Serum or Plasma | 0.57 (0.76) | 0.35 (0.59) | -0.23 | Hematocrit [Volume Fraction] of Blood | 0.48 (0.7) | 0.35 (0.6) | -0.14 |
| Carbon dioxide [Partial pressure] in Blood | 0.41 (0.64) | 0.23 (0.48) | -0.23 | An expanded problem focused history; An expanded problem focused examination; | 0.44 (0.67) | 0.32 (0.57) | -0.14 |
| Calcium.ionized/Calcium.total corrected for albumin in Blood | 0.43 (0.65) | 0.24 (0.49) | -0.23 | Platelet mean volume [Entitic volume] in Blood | 0.55 (0.74) | 0.42 (0.64) | -0.14 |
| Penicillin G potassium [Mass] of Dose | 0.43 (0.66) | 0.24 (0.49) | -0.23 | Hemoglobin [Mass/volume] in Blood | 0.49 (0.7) | 0.36 (0.6) | -0.14 |
| Cotinine/Creatinine [Mass Ratio] in Urine | 0.44 (0.66) | 0.25 (0.5) | -0.23 | General examination of patient | 0.13 (0.36) | 0.07 (0.27) | -0.14 |
| Chloride [Moles/volume] in Saliva (oral fluid) | 0.43 (0.65) | 0.24 (0.49) | -0.23 | Carbon dioxide [Partial pressure] in Blood | 0.41 (0.64) | 0.29 (0.54) | -0.14 |
| Lower proportion in vaccinated patients | | | | Lower proportion in vaccinated patients | | | |
|  |  |  |  | Any visit on day 0 | 0.54 (0.73) | 1 (1) | 0.37 |
|  |  |  |  | Outpatient visit on day 0 | 0.52 (0.72) | 0.86 (0.93) | 0.29 |
|  |  |  |  | Emergency room visit on day 0 | 0.01 (0.08) | 0.08 (0.28) | 0.24 |

**Table S8.** Top 50 covariates with the standardized difference of means (SDM) >0.1, covariate proportion and standard deviation, for comparison of COVID-19 vaccinated population indexed on the date of vaccination compared to the same population indexed on (a) a date or (b) a visit, day -180 to -450.

| **COVID-19 vaccinated patients (Target) compared to unvaccinated indexed on a date (Comparator)** | | | | **COVID-19 vaccinated patients (Target) compared to unvaccinated indexed on a visit (Comparator)** | | | |
| --- | --- | --- | --- | --- | --- | --- | --- |
| **Covariate name** | **Target, mean (SD)** | **Comparator, mean (SD)** | **SDM** | **Covariate name** | **Target, mean (SD)** | **Comparator, mean (SD)** | **SDM** |
| ***CUIMC EHR*** | | | | | | | |
| Higher proportion before vaccination | | | | Higher proportion before vaccination | | | |
| index month:1 | 0.29 (0.45) | 0.1 (0.3) | -0.49 | SARS-CoV-2 (COVID-19) RNA [Presence] in Respiratory specimen by NAA with probe detection | 0.16 (0.37) | <0.01 (0.01) | -0.63 |
| SARS-CoV-2 (COVID-19) RNA [Presence] in Respiratory specimen by NAA with probe detection | 0.1 (0.3) | <0.01 (0.01) | -0.46 | Patient encounter status | 0.36 (0.48) | 0.13 (0.34) | -0.55 |
| index month:3 | 0.27 (0.45) | 0.11 (0.32) | -0.42 | Nucleated erythrocytes [#/volume] in Blood by Automated count | 0.14 (0.35) | 0.01 (0.08) | -0.52 |
| SARS-CoV-2 (COVID-19) IgG+IgM Ab [Presence] in Serum or Plasma by Immunoassay | 0.06 (0.25) | <0.01 (0) | -0.37 | Tobacco smoking behavior - finding | 0.64 (0.48) | 0.39 (0.49) | -0.52 |
| Nucleated erythrocytes [#/volume] in Blood by Automated count | 0.08 (0.27) | 0.01 (0.09) | -0.35 | index month:1 | 0.3 (0.46) | 0.11 (0.31) | -0.48 |
| Patient encounter status | 0.21 (0.41) | 0.09 (0.29) | -0.32 | SARS-CoV-2 (COVID-19) IgG+IgM Ab [Presence] in Serum or Plasma by Immunoassay | 0.1 (0.3) | <0.01 (0) | -0.47 |
| Cardiovascular system.central US Study diagnosis | 0.05 (0.21) | <0.01 (0.03) | -0.3 | acetaminophen | 0.13 (0.34) | 0.02 (0.14) | -0.43 |
| acetaminophen | 0.07 (0.26) | 0.01 (0.11) | -0.29 | index month:3 | 0.26 (0.44) | 0.1 (0.31) | -0.42 |
| index month:2 | 0.21 (0.4) | 0.1 (0.3) | -0.29 | Cardiovascular system.central US Study diagnosis | 0.09 (0.28) | <0.01 (0.04) | -0.42 |
| Exposure to SARS-CoV-2 | 0.04 (0.19) | <0.01 (0) | -0.27 | Immature granulocytes [#/volume] in Blood | 0.1 (0.3) | 0.01 (0.1) | -0.4 |
| Immature granulocytes [#/volume] in Blood | 0.06 (0.23) | 0.01 (0.1) | -0.26 | Exposure to SARS-CoV-2 | 0.06 (0.24) | <0.01 (0.01) | -0.37 |
| Telephone evaluation and management service by a physician or other qualified health care professional who may report evaluation and management services provided to an established patient, parent, or guardian not originating from a related E/M service pro | 0.03 (0.17) | <0.01 (0.01) | -0.24 | Body temperature | 0.36 (0.48) | 0.21 (0.41) | -0.35 |
| lidocaine | 0.04 (0.2) | 0.01 (0.08) | -0.23 | Oxygen saturation in Arterial blood by Pulse oximetry | 0.42 (0.49) | 0.26 (0.44) | -0.35 |
| COVID-19 | 0.02 (0.15) | <0.01 (0.01) | -0.22 | lidocaine | 0.08 (0.27) | 0.01 (0.1) | -0.34 |
| Suspected COVID-19 | 0.02 (0.15) | <0.01 (0.01) | -0.22 | Telephone evaluation and management service by a physician or other qualified health care professional who may report evaluation and management services provided to an established patient, parent, or guardian not originating from a related E/M service pro | 0.05 (0.22) | <0.01 (0.01) | -0.33 |
| fentanyl | 0.03 (0.16) | <0.01 (0.03) | -0.22 | fentanyl | 0.05 (0.21) | <0.01 (0.03) | -0.31 |
| atorvastatin | 0.06 (0.24) | 0.02 (0.15) | -0.21 | COVID-19 | 0.05 (0.21) | <0.01 (0.01) | -0.31 |
| SARS-CoV-2 | 0.02 (0.15) | <0.01 (0) | -0.21 | atorvastatin | 0.11 (0.32) | 0.03 (0.18) | -0.31 |
| ondansetron | 0.03 (0.18) | <0.01 (0.07) | -0.2 | Suspected COVID-19 | 0.04 (0.2) | <0.01 (0.01) | -0.3 |
| Telephone evaluation and management service by a physician or other qualified health care professional who may report evaluation and management services provided to an established patient, parent, or guardian not originating from a related E/M service pro | 0.02 (0.14) | <0.01 (0.01) | -0.2 | ondansetron | 0.06 (0.24) | 0.01 (0.09) | -0.29 |
| sodium chloride | 0.03 (0.16) | <0.01 (0.05) | -0.2 | sodium chloride | 0.05 (0.21) | <0.01 (0.06) | -0.28 |
| iohexol | 0.02 (0.14) | <0.01 (0.02) | -0.2 | aspirin | 0.07 (0.26) | 0.02 (0.13) | -0.28 |
| aspirin | 0.04 (0.2) | 0.01 (0.1) | -0.19 | Telephone evaluation and management service by a physician or other qualified health care professional who may report evaluation and management services provided to an established patient, parent, or guardian not originating from a related E/M service pro | 0.04 (0.19) | <0.01 (0.01) | -0.28 |
| calcium chloride | 0.02 (0.14) | <0.01 (0.03) | -0.19 | iohexol | 0.04 (0.19) | <0.01 (0.03) | -0.27 |
| midazolam | 0.02 (0.14) | <0.01 (0.02) | -0.19 | calcium chloride | 0.04 (0.19) | <0.01 (0.02) | -0.27 |
| amlodipine | 0.05 (0.21) | 0.02 (0.12) | -0.18 | midazolam | 0.04 (0.19) | <0.01 (0.03) | -0.27 |
| phenylephrine | 0.02 (0.13) | <0.01 (0.02) | -0.18 | SARS-CoV-2 | 0.03 (0.18) | <0.01 (0.01) | -0.27 |
| propofol | 0.02 (0.13) | <0.01 (0.02) | -0.18 | index month:4 | 0.14 (0.35) | 0.06 (0.24) | -0.27 |
| albuterol | 0.04 (0.19) | 0.01 (0.1) | -0.18 | amlodipine | 0.08 (0.28) | 0.03 (0.16) | -0.26 |
| ibuprofen | 0.03 (0.16) | <0.01 (0.06) | -0.18 | albuterol | 0.07 (0.25) | 0.02 (0.13) | -0.26 |
| General finding of observation of patient | 0.02 (0.14) | <0.01 (0.05) | -0.17 | phenylephrine | 0.03 (0.18) | <0.01 (0.03) | -0.26 |
| metoprolol | 0.04 (0.18) | 0.01 (0.1) | -0.16 | propofol | 0.03 (0.18) | <0.01 (0.02) | -0.26 |
| History of SARS-CoV-2 | 0.01 (0.11) | <0.01 (0) | -0.16 | ibuprofen | 0.05 (0.21) | 0.01 (0.08) | -0.25 |
| Tobacco smoking behavior - finding | 0.36 (0.48) | 0.28 (0.45) | -0.16 | General finding of observation of patient | 0.04 (0.2) | <0.01 (0.07) | -0.24 |
| heparin | 0.01 (0.12) | <0.01 (0.03) | -0.16 | An expanded problem focused history; An expanded problem focused examination; Medical decision making of low | 0.38 (0.48) | 0.26 (0.44) | -0.24 |
| enoxaparin | 0.02 (0.12) | <0.01 (0.03) | -0.16 | Oxygen saturation in Arterial blood by Pulse oximetry | 0.33 (0.47) | 0.22 (0.41) | -0.24 |
| Allergy to penicillin | 0.02 (0.14) | <0.01 (0.05) | -0.16 | Troponin T.cardiac [Mass/volume] in Serum or Plasma by High sensitivity method | 0.06 (0.23) | 0.01 (0.12) | -0.23 |
| Cytology Cervical or vaginal smear or scraping study | 0.01 (0.11) | <0.01 (0.02) | -0.15 | metoprolol | 0.06 (0.24) | 0.02 (0.13) | -0.23 |
| Body temperature | 0.19 (0.39) | 0.14 (0.34) | -0.15 | heparin | 0.03 (0.16) | <0.01 (0.03) | -0.22 |
| famotidine | 0.02 (0.15) | <0.01 (0.07) | -0.15 | Allergy to penicillin | 0.04 (0.18) | <0.01 (0.07) | -0.22 |
| Telephone evaluation and management service by a physician or other qualified health care professional who may report evaluation and management services provided to an established patient, parent, or guardian not originating from a related E/M service pro | 0.01 (0.11) | <0.01 (0.01) | -0.15 | enoxaparin | 0.03 (0.17) | <0.01 (0.04) | -0.22 |
| cefazolin | 0.01 (0.11) | <0.01 (0.02) | -0.15 | famotidine | 0.04 (0.2) | 0.01 (0.09) | -0.22 |
| glucose | 0.01 (0.11) | <0.01 (0.02) | -0.15 | oxycodone | 0.03 (0.18) | <0.01 (0.07) | -0.21 |
| oxycodone | 0.02 (0.13) | <0.01 (0.05) | -0.15 | cefazolin | 0.02 (0.15) | <0.01 (0.02) | -0.21 |
| hydrochlorothiazide | 0.03 (0.18) | 0.01 (0.11) | -0.14 | Respiratory rate | 0.26 (0.44) | 0.17 (0.38) | -0.21 |
| Oxygen saturation in Arterial blood by Pulse oximetry | 0.23 (0.42) | 0.17 (0.38) | -0.14 | Telephone evaluation and management service by a physician or other qualified health care professional who may report evaluation and management services provided to an established patient, parent, or guardian not originating from a related E/M service pro | 0.02 (0.15) | <0.01 (0.01) | -0.21 |
| omeprazole | 0.03 (0.17) | 0.01 (0.1) | -0.14 | Body height | 0.53 (0.5) | 0.43 (0.49) | -0.21 |
| bupivacaine | 0.01 (0.1) | <0.01 (0.02) | -0.14 | docusate | 0.04 (0.19) | 0.01 (0.09) | -0.21 |
| sennosides, USP | 0.02 (0.13) | <0.01 (0.05) | -0.14 | hydrochlorothiazide | 0.06 (0.24) | 0.02 (0.14) | -0.21 |
| Lower proportion before vaccination | | | | Lower proportion before vaccination | | | |
| index month:6 | <0.01 (0) | 0.11 (0.32) | 0.5 | Any visit on day 0 | 0.03 (0.18) | 1 (0) | 7.47 |
| Nucleated erythrocytes/100 leukocytes [Ratio] in Blood | 0.01 (0.08) | 0.13 (0.33) | 0.5 | Outpatient visit on day 0 | 0.03 (0.17) | 0.71 (0.45) | 1.98 |
| Platelet mean volume [Entitic volume] in Blood | 0.01 (0.08) | 0.12 (0.33) | 0.48 | Nucleated erythrocytes/100 leukocytes [Ratio] in Blood | 0.01 (0.11) | 0.19 (0.39) | 0.62 |
| index month:7 | <0.01 (0) | 0.1 (0.3) | 0.47 | Platelet mean volume [Entitic volume] in Blood | 0.01 (0.11) | 0.18 (0.39) | 0.6 |
| Platelets [#/volume] in Blood | 0.01 (0.08) | 0.11 (0.32) | 0.46 | Platelets [#/volume] in Blood | 0.01 (0.12) | 0.17 (0.38) | 0.57 |
| index month:5 | 0.01 (0.11) | 0.12 (0.32) | 0.43 | 2019 | <0.01 (0) | 0.13 (0.33) | 0.54 |
| Hematocrit [Volume Fraction] of Blood | 0.01 (0.12) | 0.11 (0.32) | 0.41 | index month:7 | <0.01 (0) | 0.12 (0.32) | 0.52 |
| Eosinophils/100 leukocytes in Blood | <0.01 (0.06) | 0.08 (0.28) | 0.4 | index month:6 | <0.01 (0) | 0.12 (0.32) | 0.52 |
| index month:8 | <0.01 (0) | 0.07 (0.26) | 0.39 | Hematocrit [Volume Fraction] of Blood | 0.03 (0.16) | 0.17 (0.38) | 0.5 |
| Neutrophils/100 leukocytes in Blood | <0.01 (0.06) | 0.08 (0.27) | 0.39 | Eosinophils/100 leukocytes in Blood | 0.01 (0.09) | 0.13 (0.33) | 0.49 |
| Lymphocytes/100 leukocytes in Blood | <0.01 (0.06) | 0.07 (0.26) | 0.37 | Neutrophils/100 leukocytes in Blood | 0.01 (0.08) | 0.12 (0.33) | 0.48 |
| Body surface area | 0.08 (0.28) | 0.21 (0.41) | 0.36 | Lymphocytes/100 leukocytes in Blood | 0.01 (0.08) | 0.11 (0.32) | 0.46 |
| Platelet mean volume [Entitic volume] in Blood | 0.06 (0.24) | 0.16 (0.37) | 0.34 | Emergency room visit on day 0 | <0.01 (0.03) | 0.09 (0.28) | 0.43 |
| Nucleated erythrocytes [#/volume] in Blood | 0.06 (0.24) | 0.16 (0.37) | 0.33 | index month:8 | <0.01 (0) | 0.08 (0.27) | 0.42 |
| Nucleated erythrocytes/100 leukocytes [Ratio] in Blood | 0.06 (0.24) | 0.16 (0.37) | 0.33 | Urobilinogen [Mass/volume] in Urine by Test strip | 0.01 (0.08) | 0.09 (0.29) | 0.4 |
| Urobilinogen [Mass/volume] in Urine by Test strip | <0.01 (0.06) | 0.06 (0.24) | 0.33 | Urobilinogen [Units/volume] in Urine by Test strip | <0.01 (0.06) | 0.08 (0.27) | 0.39 |
| Platelets [#/volume] in Blood | 0.06 (0.24) | 0.17 (0.37) | 0.33 | Specific gravity of Urine | <0.01 (0.06) | 0.08 (0.27) | 0.39 |
| Patient encounter procedure | 0.04 (0.19) | 0.12 (0.33) | 0.33 | Patient encounter procedure | 0.07 (0.25) | 0.19 (0.39) | 0.37 |
| Specific gravity of Urine | <0.01 (0.04) | 0.05 (0.23) | 0.32 | Thyrotropin [Units/volume] in Serum or Plasma | 0.01 (0.1) | 0.08 (0.27) | 0.36 |
| Urobilinogen [Units/volume] in Urine by Test strip | <0.01 (0.04) | 0.05 (0.23) | 0.32 | Body surface area | 0.16 (0.37) | 0.31 (0.46) | 0.35 |
| Glomerular filtration rate/1.73 sq M.predicted among blacks [Volume Rate/Area] in Serum, Plasma or Blood by Creatinine-based formula (MDRD) | 0.08 (0.26) | 0.17 (0.38) | 0.3 | Platelet mean volume [Entitic volume] in Blood | 0.11 (0.32) | 0.24 (0.43) | 0.35 |
| Glomerular filtration rate/1.73 sq M.predicted among non-blacks [Volume Rate/Area] in Serum, Plasma or Blood by Creatinine-based formula (MDRD) | 0.07 (0.26) | 0.17 (0.38) | 0.3 | Nucleated erythrocytes [#/volume] in Blood | 0.11 (0.31) | 0.24 (0.43) | 0.35 |
| Hematocrit [Volume Fraction] of Blood | 0.08 (0.27) | 0.18 (0.38) | 0.3 | Nucleated erythrocytes/100 leukocytes [Ratio] in Blood | 0.11 (0.31) | 0.24 (0.43) | 0.35 |
| index month:11 | <0.01 (0) | 0.04 (0.2) | 0.3 | Platelets [#/volume] in Blood | 0.12 (0.32) | 0.25 (0.43) | 0.34 |
| Thyrotropin [Units/volume] in Serum or Plasma | <0.01 (0.07) | 0.05 (0.22) | 0.29 | index month:5 | 0.01 (0.11) | 0.08 (0.27) | 0.32 |
| Basophils [#/volume] in Blood | 0.04 (0.2) | 0.12 (0.32) | 0.29 | Leukocytes [#/volume] in Urine | <0.01 (0.05) | 0.05 (0.22) | 0.31 |
| Eosinophils [#/volume] in Blood | 0.04 (0.2) | 0.12 (0.32) | 0.29 | Glomerular filtration rate/1.73 sq M.predicted among blacks [Volume Rate/Area] in Serum, Plasma or Blood by Creatinine-based formula (MDRD) | 0.14 (0.35) | 0.26 (0.44) | 0.31 |
| Lymphocytes [#/volume] in Blood | 0.04 (0.2) | 0.12 (0.32) | 0.29 | Glomerular filtration rate/1.73 sq M.predicted among non-blacks [Volume Rate/Area] in Serum, Plasma or Blood by Creatinine-based formula (MDRD) | 0.14 (0.34) | 0.26 (0.44) | 0.31 |
| Eosinophils/100 leukocytes in Blood | 0.04 (0.2) | 0.12 (0.32) | 0.29 | index month:11 | <0.01 (0) | 0.04 (0.21) | 0.3 |
| Neutrophils/100 leukocytes in Blood | 0.04 (0.2) | 0.12 (0.33) | 0.29 | index month:9 | <0.01 (0) | 0.04 (0.21) | 0.3 |
| Lymphocytes/100 leukocytes in Blood | 0.04 (0.2) | 0.12 (0.33) | 0.29 | Cholesterol in LDL [Mass/volume] in Serum or Plasma | 0.05 (0.21) | 0.13 (0.34) | 0.3 |
| Neutrophils [#/volume] in Blood | 0.05 (0.21) | 0.12 (0.33) | 0.28 | Hematocrit [Volume Fraction] of Blood | 0.15 (0.35) | 0.27 (0.44) | 0.3 |
| index month:9 | <0.01 (0) | 0.04 (0.18) | 0.27 | Basophils [#/volume] in Blood | 0.08 (0.27) | 0.18 (0.38) | 0.3 |
| Specific gravity of Urine | 0.02 (0.16) | 0.08 (0.28) | 0.26 | Eosinophils [#/volume] in Blood | 0.08 (0.27) | 0.18 (0.38) | 0.3 |
| Cholesterol in LDL [Mass/volume] in Serum or Plasma | 0.02 (0.15) | 0.08 (0.27) | 0.25 | Lymphocytes [#/volume] in Blood | 0.08 (0.27) | 0.18 (0.38) | 0.3 |
| Leukocytes [#/volume] in Urine | <0.01 (0.03) | 0.03 (0.18) | 0.25 | Eosinophils/100 leukocytes in Blood | 0.08 (0.27) | 0.18 (0.38) | 0.3 |
| Urobilinogen [Units/volume] in Urine by Test strip | 0.02 (0.15) | 0.07 (0.26) | 0.25 | Lymphocytes/100 leukocytes in Blood | 0.08 (0.27) | 0.18 (0.38) | 0.3 |
| Ketones [Presence] in Urine by Test strip | 0.03 (0.17) | 0.08 (0.28) | 0.24 | Neutrophils/100 leukocytes in Blood | 0.08 (0.27) | 0.18 (0.38) | 0.3 |
| Glucose [Presence] in Urine by Test strip | 0.03 (0.17) | 0.08 (0.28) | 0.24 | Neutrophils [#/volume] in Blood | 0.09 (0.28) | 0.18 (0.39) | 0.29 |
| Body height Measured | 0.04 (0.19) | 0.1 (0.3) | 0.24 | Erythrocytes [#/volume] in Urine | <0.01 (0.04) | 0.04 (0.2) | 0.28 |
| Thyrotropin [Units/volume] in Serum or Plasma | 0.03 (0.17) | 0.08 (0.27) | 0.23 | Specific gravity of Urine | 0.05 (0.21) | 0.12 (0.33) | 0.28 |
| Leukocytes [#/volume] in Urine | 0.02 (0.13) | 0.06 (0.24) | 0.23 | Urobilinogen [Units/volume] in Urine by Test strip | 0.04 (0.2) | 0.11 (0.32) | 0.27 |
| index month:10 | <0.01 (0) | 0.02 (0.16) | 0.22 | Body height Measured | 0.07 (0.26) | 0.15 (0.36) | 0.26 |
| Erythrocytes [#/volume] in Urine | <0.01 (0.03) | 0.03 (0.16) | 0.22 | Thyrotropin [Units/volume] in Serum or Plasma | 0.05 (0.22) | 0.12 (0.33) | 0.25 |
| Erythrocytes [#/volume] in Urine | 0.02 (0.13) | 0.06 (0.24) | 0.22 | Lymphocytes/100 leukocytes in Blood | <0.01 (0.05) | 0.04 (0.19) | 0.25 |
| Hepatitis C virus Ab [Presence] in Serum | 0.01 (0.09) | 0.04 (0.2) | 0.21 | Ketones [Presence] in Urine by Test strip | 0.05 (0.23) | 0.12 (0.33) | 0.25 |
| Oxygen saturation Calculated from oxygen partial pressure in Blood | 0.04 (0.19) | 0.09 (0.29) | 0.21 | Glucose [Presence] in Urine by Test strip | 0.05 (0.22) | 0.12 (0.33) | 0.25 |
| First Respiration rate Set | 0.03 (0.18) | 0.08 (0.27) | 0.21 | index month:10 | <0.01 (0.01) | 0.03 (0.17) | 0.24 |
| ***Optum EHR*** | | | | | | | |
| Higher proportion before vaccination | | | | Higher proportion before vaccination | | | |
| 2021 | 0.83 (0.91) | <0.01 (0) | -0.91 | 2021 | 0.84 (0.91) | <0.01 (0) | -0.91 |
| Outpatient visit on day 0 | 0.72 (0.85) | 0.06 (0.24) | -0.76 | SARS-CoV-2 (COVID-19) RNA [Presence] in Respiratory specimen by NAA with probe detection | 0.31 (0.55) | <0.01 (0) | -0.55 |
| Any visit on day 0 | 0.73 (0.85) | 0.06 (0.25) | -0.75 | Exposure to viral disease | 0.23 (0.48) | <0.01 (0.06) | -0.47 |
| SARS-CoV-2 (COVID-19) RNA [Presence] in Respiratory specimen by NAA with probe detection | 0.28 (0.53) | <0.01 (0) | -0.53 | Infectious agent detection by nucleic acid (DNA or RNA); severe acute respiratory syndrome coronavirus 2 (SARS-CoV-2) (Coronavirus disease [COVID-19]), amplified probe technique, making use of high throughput technologies as described by CMS-2020-01-R | 0.14 (0.38) | <0.01 (0) | -0.38 |
| Exposure to viral disease | 0.21 (0.46) | <0.01 (0.05) | -0.45 | Infectious agent detection by nucleic acid (DNA or RNA); severe acute respiratory syndrome coronavirus 2 (SARS-CoV-2) (Coronavirus disease [COVID-19]), amplified probe technique | 0.13 (0.36) | <0.01 (0) | -0.36 |
| Infectious agent detection by nucleic acid (DNA or RNA); severe acute respiratory syndrome coronavirus 2 (SARS-CoV-2) (Coronavirus disease [COVID-19]), amplified probe technique, making use of high throughput technologies as described by CMS-2020-01-R | 0.13 (0.37) | <0.01 (0) | -0.37 | Glucose [Mass/volume] in Serum or Plasma | 1.62 (1.27) | 1.1 (1.05) | -0.31 |
| Infectious agent detection by nucleic acid (DNA or RNA); severe acute respiratory syndrome coronavirus 2 (SARS-CoV-2) (Coronavirus disease [COVID-19]), amplified probe technique | 0.12 (0.35) | <0.01 (0) | -0.35 | index month:3 | 0.3 (0.54) | 0.1 (0.32) | -0.3 |
| index month:1 | 0.3 (0.54) | 0.11 (0.34) | -0.29 | index month:1 | 0.29 (0.54) | 0.11 (0.33) | -0.29 |
| index month:3 | 0.29 (0.54) | 0.11 (0.34) | -0.28 | Laboratory - Bacteriology and Microbiology | 0.28 (0.53) | 0.1 (0.32) | -0.29 |
| Laboratory - Bacteriology and Microbiology | 0.26 (0.51) | 0.1 (0.32) | -0.27 | index month:2 | 0.25 (0.5) | 0.1 (0.31) | -0.26 |
| Glucose [Mass/volume] in Serum or Plasma | 1.49 (1.22) | 1.08 (1.04) | -0.26 | Hemoglobin [Mass/volume] in Blood | 0.74 (0.86) | 0.49 (0.7) | -0.22 |
| index month:2 | 0.25 (0.5) | 0.11 (0.33) | -0.24 | Measurement of Severe acute respiratory syndrome coronavirus 2 (SARS-CoV-2) | 0.05 (0.22) | <0.01 (0) | -0.22 |
| Measurement of Severe acute respiratory syndrome coronavirus 2 (SARS-CoV-2) | 0.04 (0.21) | <0.01 (0) | -0.21 | Telephone evaluation and management service by a physician or other qualified health care professional who may report evaluation and management services provided to an established patient, parent, or guardian | 0.05 (0.22) | <0.01 (0.01) | -0.22 |
| Telephone evaluation and management service by a physician or other qualified health care professional who may report evaluation and management services provided to an established patient, parent, or guardian | 0.04 (0.21) | <0.01 (0.01) | -0.21 | Clinical decision support mechanism national decision support company, as defined by the medicare appropriate use criteria program | 0.05 (0.22) | <0.01 (0.03) | -0.21 |
| Clinical decision support mechanism national decision support company, as defined by the medicare appropriate use criteria program | 0.04 (0.21) | <0.01 (0.03) | -0.2 | Hematocrit [Volume Fraction] of Blood | 0.62 (0.79) | 0.42 (0.65) | -0.2 |
| Hemoglobin [Mass/volume] in Blood | 0.68 (0.82) | 0.48 (0.69) | -0.19 | COVID-19 | 0.04 (0.2) | <0.01 (0) | -0.2 |
| COVID-19 | 0.04 (0.19) | <0.01 (0) | -0.19 | Patient meets COVID-19 laboratory diagnostic criteria | 0.04 (0.2) | <0.01 (0) | -0.2 |
| Patient meets COVID-19 laboratory diagnostic criteria | 0.04 (0.19) | <0.01 (0) | -0.19 | Basophils/100 leukocytes in Blood | 1.76 (1.33) | 1.41 (1.19) | -0.2 |
| General finding of observation of patient | 0.07 (0.26) | 0.01 (0.12) | -0.19 | General finding of observation of patient | 0.07 (0.27) | 0.02 (0.12) | -0.19 |
| 2019-ncov coronavirus, sars-cov-2/2019-ncov (covid-19), any technique, multiple types or subtypes (includes all targets), non-cdc | 0.03 (0.18) | <0.01 (0) | -0.18 | Erythrocytes [#/volume] in Blood | 0.63 (0.79) | 0.43 (0.66) | -0.19 |
| Hospital outpatient clinic visit specimen collection for severe acute respiratory syndrome coronavirus 2 (sars-cov-2) (coronavirus disease [covid-19]) | 0.03 (0.17) | <0.01 (0) | -0.17 | 2019-ncov coronavirus, sars-cov-2/2019-ncov (covid-19), any technique, multiple types or subtypes (includes all targets), non-cdc | 0.03 (0.19) | <0.01 (0) | -0.18 |
| Hematocrit [Volume Fraction] of Blood | 0.57 (0.76) | 0.41 (0.64) | -0.17 | Penicillin G potassium [Mass] of Dose | 1.52 (1.23) | 1.23 (1.11) | -0.18 |
| Measurement of Severe acute respiratory syndrome coronavirus 2 antibody | 0.03 (0.17) | <0.01 (0) | -0.17 | MCH [Entitic mass] | 1.19 (1.09) | 0.94 (0.97) | -0.18 |
| Erythrocytes [#/volume] in Blood | 0.58 (0.76) | 0.42 (0.65) | -0.16 | Hospital outpatient clinic visit specimen collection for severe acute respiratory syndrome coronavirus 2 (sars-cov-2) (coronavirus disease [covid-19]) | 0.03 (0.18) | <0.01 (0) | -0.18 |
| Basophils/100 leukocytes in Blood | 1.63 (1.28) | 1.36 (1.17) | -0.16 | Lymphocytes/100 leukocytes in Blood | 0.63 (0.8) | 0.45 (0.67) | -0.18 |
| Telephone evaluation and management service by a physician or other qualified health care professional who may report evaluation and management services provided to an established patient, parent, or guardian | 0.02 (0.15) | <0.01 (0.01) | -0.15 | Measurement of Severe acute respiratory syndrome coronavirus 2 antibody | 0.03 (0.18) | <0.01 (0) | -0.18 |
| Lymphocytes/100 leukocytes in Blood | 0.59 (0.77) | 0.43 (0.66) | -0.15 | Eosinophils/100 leukocytes in Blood | 1.62 (1.27) | 1.33 (1.15) | -0.17 |
| Telephone evaluation and management service by a physician or other qualified health care professional who may report evaluation and management services provided to an established patient, parent, or guardian | 0.02 (0.15) | <0.01 (0.01) | -0.15 | index month:12 | 0.16 (0.4) | 0.08 (0.28) | -0.17 |
| index month:12 | 0.16 (0.4) | 0.09 (0.3) | -0.15 | Magnesium [Presence] in Stone | 0.32 (0.57) | 0.2 (0.45) | -0.17 |
| Penicillin G potassium [Mass] of Dose | 1.41 (1.19) | 1.18 (1.09) | -0.14 | Platelet mean volume [Entitic volume] in Blood | 0.88 (0.94) | 0.68 (0.82) | -0.17 |
| Magnesium [Presence] in Stone | 0.3 (0.55) | 0.2 (0.44) | -0.14 | MCV [Entitic volume] | 1.15 (1.07) | 0.92 (0.96) | -0.16 |
| Calcium.ionized/Calcium.total corrected for albumin in Blood | 0.18 (0.42) | 0.1 (0.32) | -0.14 | Calcium.ionized/Calcium.total corrected for albumin in Blood | 0.19 (0.44) | 0.1 (0.32) | -0.16 |
| lisinopril 10 MG Oral Tablet | 0.05 (0.23) | 0.02 (0.13) | -0.14 | Sodium [Moles/volume] in Saliva (oral fluid) | 0.24 (0.49) | 0.14 (0.38) | -0.16 |
| Eosinophils/100 leukocytes in Blood | 1.5 (1.23) | 1.27 (1.13) | -0.14 | Platelets [#/volume] in Blood | 1.21 (1.1) | 0.97 (0.99) | -0.16 |
| MCH [Entitic mass] | 1.1 (1.05) | 0.91 (0.95) | -0.14 | Telephone evaluation and management service by a physician or other qualified health care professional who may report evaluation and management services provided to an established patient, parent, or guardian | 0.03 (0.16) | <0.01 (0.01) | -0.16 |
| Platelet mean volume [Entitic volume] in Blood | 0.82 (0.9) | 0.65 (0.81) | -0.14 | MCHC [Mass/volume] | 1.15 (1.07) | 0.92 (0.96) | -0.16 |
| Sodium [Moles/volume] in Saliva (oral fluid) | 0.22 (0.47) | 0.14 (0.38) | -0.14 | Chloride [Moles/volume] in Saliva (oral fluid) | 1.36 (1.17) | 1.12 (1.06) | -0.15 |
| Viral screening | 0.04 (0.21) | 0.01 (0.11) | -0.13 | Monocytes/100 leukocytes in Blood | 1.43 (1.19) | 1.18 (1.09) | -0.15 |
| Ferritin [Interpretation] in Blood | 0.05 (0.22) | 0.01 (0.12) | -0.13 | Anion gap in Blood | 0.9 (0.95) | 0.71 (0.84) | -0.15 |
| MCV [Entitic volume] | 1.07 (1.03) | 0.89 (0.94) | -0.13 | Telephone evaluation and management service by a physician or other qualified health care professional who may report evaluation and management services provided to an established patient, parent, or guardian | 0.02 (0.15) | <0.01 (0.01) | -0.15 |
| Platelets [#/volume] in Blood | 1.12 (1.06) | 0.94 (0.97) | -0.12 | Sodium [Moles/volume] in Saliva (oral fluid) | 1.42 (1.19) | 1.18 (1.09) | -0.15 |
| visit_occurrence concept count during day -450 through -181 concept_count relative to index | 17.42 (19.35) | 14.22 (17.33) | -0.12 | visit_occurrence concept count during day -450 through -181 concept_count relative to index | 18.84 (19.36) | 15.1 (17.34) | -0.14 |
| Monocytes/100 leukocytes in Blood | 1.32 (1.15) | 1.13 (1.06) | -0.12 | lisinopril 10 MG Oral Tablet | 0.06 (0.24) | 0.02 (0.14) | -0.14 |
| MCHC [Mass/volume] | 1.06 (1.03) | 0.9 (0.95) | -0.12 | Leukocytes [#/volume] in Blood | 1.23 (1.11) | 1.01 (1.01) | -0.14 |
| Anion gap in Blood | 0.83 (0.91) | 0.69 (0.83) | -0.12 | Calcium.ionized/Calcium.total corrected for albumin in Blood | 1.43 (1.2) | 1.2 (1.1) | -0.14 |
| Chloride [Moles/volume] in Saliva (oral fluid) | 1.26 (1.12) | 1.08 (1.04) | -0.12 | Viral screening | 0.05 (0.21) | 0.01 (0.11) | -0.14 |
| Albumin [Presence] in Urine | 0.14 (0.38) | 0.09 (0.29) | -0.12 | Carbon dioxide [Partial pressure] in Blood | 1.27 (1.13) | 1.06 (1.03) | -0.14 |
| Telehealth originating site facility fee | 0.01 (0.12) | <0.01 (0.02) | -0.12 | Ferritin [Interpretation] in Blood | 0.05 (0.22) | 0.02 (0.12) | -0.14 |
| Chloride [Moles/volume] in Saliva (oral fluid) | 0.15 (0.38) | 0.09 (0.3) | -0.12 | Albumin [Presence] in Urine | 0.15 (0.39) | 0.09 (0.3) | -0.14 |
| Sodium [Moles/volume] in Saliva (oral fluid) | 1.31 (1.14) | 1.13 (1.06) | -0.11 | Glucose [Mass/volume] in Serum or Plasma | 0.99 (0.99) | 0.81 (0.9) | -0.13 |
| Lower proportion before vaccination | | | | Lower proportion before vaccination | | | |
| index month:5 | <0.01 (0) | 0.11 (0.34) | 0.66 | index month:6 | <0.01 (0) | 0.14 (0.37) | 0.37 |
| index month:4 | <0.01 (0) | 0.11 (0.33) | 0.42 | index month:5 | <0.01 (0) | 0.11 (0.33) | 0.33 |
| index month:6 | <0.01 (0) | 0.11 (0.33) | 0.34 | index month:7 | <0.01 (0) | 0.11 (0.33) | 0.33 |
| index month:7 | <0.01 (0) | 0.09 (0.29) | 0.33 | index month:4 | <0.01 (0) | 0.09 (0.3) | 0.3 |
| index month:11 | <0.01 (0.01) | 0.06 (0.24) | 0.33 | index month:8 | <0.01 (0.01) | 0.06 (0.25) | 0.25 |
| index month:8 | <0.01 (0.02) | 0.05 (0.23) | 0.29 | index month:11 | <0.01 (0.01) | 0.05 (0.23) | 0.23 |
| 0.5 ML influenza A virus A/North Carolina/04/2016 (H3N2) antigen 0.03 MG/ML / influenza A virus A/Singapore/GP1908/2015 (H1N1) antigen 0.03 MG/ML / influenza B virus B/Iowa/06/2017 antigen 0.03 MG/ML / influenza B virus B... | 0.2 (0.45) | 0.34 (0.59) | 0.24 | 0.5 ML influenza A virus A/North Carolina/04/2016 (H3N2) antigen 0.03 MG/ML / influenza A virus A/Singapore/GP1908/2015 (H1N1) antigen 0.03 MG/ML / influenza B virus B/Iowa/06/2017 antigen 0.03 MG/ML / influenza B virus B... | 0.21 (0.46) | 0.38 (0.61) | 0.22 |
| index month:10 | <0.01 (0.01) | 0.03 (0.16) | 0.23 | Any visit on day 0 | 0.73 (0.85) | 1 (1) | 0.21 |
| index month:9 | <0.01 (0.01) | 0.02 (0.14) | 0.2 | Emergency room visit on day 0 | <0.01 (0.03) | 0.03 (0.17) | 0.16 |
| Acute glomerulonephritis | 0.01 (0.08) | 0.03 (0.18) | 0.16 | index month:10 | <0.01 (0.01) | 0.03 (0.16) | 0.16 |
| Administration of influenza vaccine | 0.03 (0.16) | 0.06 (0.25) | 0.14 | index month:9 | <0.01 (0.01) | 0.02 (0.15) | 0.15 |
|  |  |  | 0.14 | Acute glomerulonephritis | 0.01 (0.08) | 0.04 (0.19) | 0.14 |
|  |  |  | 0.13 | Inpatient visit on day 0 | <0.01 (0.03) | 0.02 (0.15) | 0.14 |
|  |  |  |  | Administration of influenza vaccine | 0.03 (0.17) | 0.07 (0.27) | 0.14 |
|  |  |  |  | Outpatient visit on day 0 | 0.72 (0.85) | 0.89 (0.94) | 0.13 |
|  |  |  |  | influenza, high dose seasonal, preservative-free | 0.02 (0.13) | 0.05 (0.22) | 0.11 |

**Table S9.** Top 50 covariates with the standardized difference of means (SDM) >0.1, covariate proportion and standard deviation, for comparison of influenza vaccinated population indexed on the date of vaccination compared to the same population indexed on (a) a date or (b) a visit, day -180 to -450.

| **Influenza vaccinated patients (Target) compared to unvaccinated indexed on a date (Comparator)** | | | | **Influenza vaccinated patients (Target) compared to unvaccinated indexed on a visit (Comparator)** | | | |
| --- | --- | --- | --- | --- | --- | --- | --- |
| **Covariate name** | **Target, mean (SD)** | **Comparator, mean (SD)** | **SDM** | **Covariate name** | **Target, mean (SD)** | **Comparator, mean (SD)** | **SDM** |
| ***CUIMC EHR*** | | | | | | | |
| Higher proportion before vaccination | | | | Higher proportion before vaccination | | | |
| Glomerular filtration rate/1.73 sq M.predicted [Volume Rate/Area] in Serum, Plasma or Blood by Creatinine-based formula (MDRD) | 0.26 (0.44) | 0.11 (0.31) | -0.39 | Systolic blood pressure | 0.89 (0.32) | 0.67 (0.47) | -0.54 |
| Platelet mean volume [Entitic volume] in Blood by Automated count | 0.25 (0.43) | 0.11 (0.31) | -0.37 | Diastolic blood pressure | 0.88 (0.32) | 0.67 (0.47) | -0.54 |
| Platelets [#/volume] in Blood by Automated count | 0.26 (0.44) | 0.13 (0.33) | -0.35 | Heart rate | 0.86 (0.35) | 0.64 (0.48) | -0.53 |
| Platelet mean volume [Entitic volume] in Blood by Automated count | 0.19 (0.39) | 0.09 (0.28) | -0.3 | Body weight | 0.89 (0.31) | 0.7 (0.46) | -0.5 |
| index month:6 | 0.11 (0.32) | 0.04 (0.19) | -0.29 | index month:10 | 0.29 (0.45) | 0.1 (0.3) | -0.49 |
| Hyaline casts [#/area] in Urine sediment by Microscopy low power field | 0.05 (0.22) | <0.01 (0.06) | -0.28 | Body mass index (BMI) [Ratio] | 0.85 (0.36) | 0.65 (0.48) | -0.48 |
| Platelets [#/volume] in Blood by Automated count | 0.19 (0.39) | 0.09 (0.29) | -0.28 | Consultation for malignant neoplasm disease | 0.08 (0.28) | <0.01 (0.06) | -0.4 |
| P wave Atrium by EKG | 0.09 (0.28) | 0.03 (0.17) | -0.24 | Body height Measured | 0.58 (0.49) | 0.38 (0.49) | -0.4 |
| P wave axis | 0.09 (0.28) | 0.03 (0.17) | -0.24 | Tobacco smoking behavior - finding | 0.67 (0.47) | 0.48 (0.5) | -0.39 |
| P-R Interval | 0.09 (0.28) | 0.03 (0.17) | -0.24 | Body temperature | 0.7 (0.46) | 0.52 (0.5) | -0.36 |
| R wave axis | 0.09 (0.28) | 0.03 (0.17) | -0.24 | Respiratory rate | 0.56 (0.5) | 0.4 (0.49) | -0.32 |
| QRS duration | 0.09 (0.28) | 0.03 (0.17) | -0.24 | Patient encounter procedure | 0.52 (0.5) | 0.38 (0.48) | -0.29 |
| Q-T interval | 0.09 (0.28) | 0.03 (0.17) | -0.24 | index month:9 | 0.18 (0.38) | 0.09 (0.28) | -0.27 |
| Q-T interval corrected | 0.09 (0.28) | 0.03 (0.17) | -0.24 | Influenza, injectable, quadrivalent, preservative free | 0.24 (0.43) | 0.14 (0.34) | -0.27 |
| T wave axis | 0.09 (0.28) | 0.03 (0.17) | -0.24 | Body height [Percentile] | 0.27 (0.45) | 0.17 (0.38) | -0.25 |
| QRS complex Ventricles by EKG | 0.09 (0.28) | 0.03 (0.17) | -0.24 | Iron/Transferrin [Ratio] in Serum or Plasma | 0.04 (0.19) | <0.01 (0.07) | -0.24 |
| Screening digital breast tomosynthesis, bilateral for primary procedure) | 0.03 (0.18) | <0.01 (0.04) | -0.24 | index month:11 | 0.19 (0.39) | 0.1 (0.31) | -0.23 |
| Specific gravity of Urine by Test strip | 0.13 (0.34) | 0.06 (0.24) | -0.24 | Angiotensin converting enzyme [Enzymatic activity/volume] in Serum or Plasma | 0.04 (0.2) | 0.01 (0.08) | -0.23 |
| index month:5 | 0.12 (0.32) | 0.05 (0.22) | -0.23 | 0.5 ML influenza A virus A/Hong Kong/4801/2014 (H3N2) antigen 0.12 MG/ML / influenza A virus A/Michigan/45/2015 (H1N1) antigen 0.12 MG/ML / influenza B virus B/Brisbane/60/2008 antigen 0.12 MG/ML Prefilled Syringe [Fluzone 2017-2018] | 0.03 (0.18) | <0.01 (0.05) | -0.23 |
| Epithelial cells.squamous [#/area] in Urine sediment by Microscopy high power field | 0.05 (0.22) | 0.01 (0.11) | -0.22 | influenza, high dose seasonal, preservative-free | 0.05 (0.22) | 0.01 (0.12) | -0.22 |
| Hyaline casts [#/area] in Urine sediment by Microscopy low power field | 0.08 (0.26) | 0.03 (0.17) | -0.22 | Heart rate | 0.37 (0.48) | 0.28 (0.45) | -0.2 |
| C reactive protein [Mass/volume] in Serum or Plasma by High sensitivity method | 0.03 (0.17) | <0.01 (0.05) | -0.21 | Systolic blood pressure | 0.38 (0.48) | 0.29 (0.45) | -0.18 |
| Bordetella parapertussis DNA [Presence] in Nasopharynx by NAA with probe detection | 0.02 (0.14) | <0.01 (0) | -0.2 | Diastolic blood pressure | 0.38 (0.48) | 0.29 (0.45) | -0.18 |
| ABO and Rh group [Type] in Blood | 0.05 (0.23) | 0.02 (0.13) | -0.19 | Body weight | 0.4 (0.49) | 0.31 (0.46) | -0.18 |
| Blood typing, serologic; ABO | 0.05 (0.23) | 0.02 (0.14) | -0.19 | Oxygen saturation in Arterial blood by Pulse oximetry | 0.25 (0.44) | 0.18 (0.39) | -0.17 |
| Cytopathology, cervical or vaginal (any reporting system), collected in preservative fluid, automated thin layer preparation; manual screening under physician supervision | 0.02 (0.14) | <0.01 (0.03) | -0.18 | Glomerular filtration rate/1.73 sq M.predicted [Volume Rate/Area] in Serum, Plasma or Blood | 0.01 (0.12) | <0.01 (0.01) | -0.17 |
| Specimen type | 0.04 (0.2) | 0.01 (0.12) | -0.18 | Body height | 0.36 (0.48) | 0.28 (0.45) | -0.16 |
| Level VI - Surgical pathology, gross and microscopic examination Bone resection Breast, mastectomy - with regional lymph nodes Colon, segmental resection for tumor Colon, total resection Esophagus, partial/total resection Extremity, disarticulation Fetus, | 0.05 (0.23) | 0.02 (0.14) | -0.18 | Body height | 0.44 (0.5) | 0.36 (0.48) | -0.16 |
| Specific gravity of Urine by Test strip | 0.09 (0.28) | 0.04 (0.21) | -0.18 | Hyaline casts [#/area] in Urine sediment by Microscopy low power field | 0.02 (0.13) | <0.01 (0.03) | -0.16 |
| Chlamydophila pneumoniae DNA [Presence] in Nasopharynx by NAA with probe detection | 0.02 (0.14) | <0.01 (0.05) | -0.17 | Iron/Transferrin [Ratio] in Serum or Plasma | 0.02 (0.13) | <0.01 (0.05) | -0.16 |
| Mycoplasma pneumoniae DNA [Presence] in Nasopharynx by NAA with probe detection | 0.02 (0.14) | <0.01 (0.05) | -0.17 | Dry body weight Measured | 0.18 (0.38) | 0.12 (0.32) | -0.16 |
| C reactive protein [Mass/volume] in Serum or Plasma by High sensitivity method | 0.02 (0.13) | <0.01 (0.03) | -0.17 | Oxygen saturation Calculated from oxygen partial pressure in Blood | 0.23 (0.42) | 0.17 (0.38) | -0.16 |
| index month:7 | 0.1 (0.3) | 0.06 (0.23) | -0.17 | 0.5 ML influenza A virus A/Michigan/45/2015 (H1N1) antigen 0.12 MG/ML / influenza A virus A/Singapore/INFIMH-16-0019/2016 (H3N2) antigen 0.12 MG/ML / influenza B virus B/Maryland/15/2016 antigen 0.12 MG/ML Prefilled Syringe [Fluzone 2018-2019] | 0.02 (0.12) | <0.01 (0.04) | -0.16 |
| 0.5 ML influenza A virus A/Singapore/GP1908/2015 (H1N1) antigen 0.03 MG/ML / influenza A virus A/Singapore/GP2050/2015 (H3N2) antigen 0.03 MG/ML / influenza B virus B/Hong Kong/259/2010 antigen 0.03 MG/ML / influenza B virus B/Utah/9/2014 antigen 0.03... | 0.01 (0.12) | <0.01 (0) | -0.17 | Carbon dioxide, total [Moles/volume] in Blood | 0.03 (0.17) | 0.01 (0.1) | -0.16 |
| age group: 70 - 74 | 0.1 (0.3) | 0.06 (0.23) | -0.16 | Oxygen saturation in Arterial blood by Pulse oximetry | 0.22 (0.41) | 0.16 (0.36) | -0.15 |
| Microscopic observation [Identifier] in Cervix by Cyto stain.thin prep | 0.02 (0.13) | <0.01 (0.04) | -0.16 | Immature granulocytes/100 leukocytes in Blood | 0.25 (0.43) | 0.19 (0.39) | -0.15 |
| age group: 65 - 69 | 0.11 (0.31) | 0.06 (0.24) | -0.16 | Immature granulocytes [#/volume] in Blood | 0.25 (0.43) | 0.19 (0.39) | -0.15 |
| Measles virus IgG Ab [Presence] in Serum by Immunoassay | 0.02 (0.15) | <0.01 (0.07) | -0.15 | Angiotensin converting enzyme [Enzymatic activity/volume] in Serum or Plasma | 0.02 (0.14) | <0.01 (0.06) | -0.15 |
| Electrocardiogram, routine ECG with at least 12 leads; with interpretation and report | 0.13 (0.34) | 0.08 (0.28) | -0.15 | Anion gap in Serum or Plasma | 0.13 (0.34) | 0.09 (0.28) | -0.15 |
| Mycobacterium tuberculosis stimulated gamma interferon release by CD4+ and CD8+ T-cells [Units/volume] corrected for background in Blood | 0.01 (0.11) | <0.01 (0) | -0.15 | ABO group [Type] in Blood by Confirmatory method | 0.01 (0.11) | <0.01 (0.03) | -0.15 |
| Mitogen stimulated gamma interferon [Units/volume] corrected for background in Blood | 0.01 (0.11) | <0.01 (0) | -0.15 | Erythrocyte distribution width [Ratio] by Automated count | 0.3 (0.46) | 0.24 (0.43) | -0.14 |
| Mycobacterium tuberculosis stimulated gamma interferon [Interpretation] in Blood Qualitative | 0.01 (0.11) | <0.01 (0.01) | -0.15 | Carbon dioxide, total [Moles/volume] in Blood | 0.02 (0.14) | 0.01 (0.07) | -0.13 |
| Troponin T.cardiac [Mass/volume] in Serum or Plasma by High sensitivity method | 0.01 (0.1) | <0.01 (0) | -0.15 | pH of Urine by Test strip | 0.05 (0.22) | 0.02 (0.16) | -0.13 |
| Gamma interferon background [Units/volume] in Blood by Immunoassay | 0.01 (0.11) | <0.01 (0.02) | -0.15 | pantoprazole 40 MG Delayed Release Oral Tablet | 0.03 (0.18) | 0.01 (0.12) | -0.13 |
| 0.5 ML hydromorphone hydrochloride 1 MG/ML Prefilled Syringe | 0.01 (0.1) | <0.01 (0.01) | -0.15 | Iron/Transferrin [Ratio] in Serum or Plasma | 0.01 (0.11) | <0.01 (0.04) | -0.13 |
| Iron/Transferrin [Ratio] in Serum or Plasma | 0.02 (0.13) | <0.01 (0.06) | -0.15 | Angiotensin converting enzyme [Enzymatic activity/volume] in Serum or Plasma | 0.01 (0.11) | <0.01 (0.04) | -0.13 |
| 0.5 ML influenza A virus A/Brisbane/02/2018 (H1N1) antigen 0.03 MG/ML / influenza A virus A/Kansas/14/2017 (H3N2) antigen 0.03 MG/ML / influenza B virus B/Maryland/15/2016 antigen 0.03 MG/ML / influenza B virus B/Phuket/3073/2013 antigen 0.03 MG/ML Pre... | 0.01 (0.1) | <0.01 (0) | -0.14 | Blood [Presence] in Urine by Visual | 0.03 (0.18) | 0.01 (0.12) | -0.12 |
| Blood product type | 0.01 (0.1) | <0.01 (0) | -0.14 | Bilirubin [Presence] in Urine by Confirmatory method | 0.03 (0.18) | 0.01 (0.12) | -0.12 |
| Lower proportion before vaccination | | | | Lower proportion before vaccination | | | |
| Influenza, seasonal, injectable | <0.01 (0.01) | 0.12 (0.32) | 0.51 | Any visit on day 0 | 0.77 (0.42) | 1 (0) | 0.76 |
| Microscopic exam [Interpretation] of Urine by Cytology | <0.01 (0.02) | 0.11 (0.32) | 0.5 | Emergency room visit on day 0 | <0.01 (0.04) | 0.1 (0.3) | 0.45 |
| age group: 0-4 | <0.01 (0) | 0.1 (0.3) | 0.48 | Outpatient visit on day 0 | 0.77 (0.42) | 0.91 (0.29) | 0.37 |
| age group: 5-9 | <0.01 (0) | 0.1 (0.3) | 0.46 | Triglyceride [Mass/volume] in Serum or Plasma | <0.01 (0.06) | 0.07 (0.25) | 0.35 |
| age group: 10-14 | <0.01 (0.04) | 0.1 (0.3) | 0.46 | index month:3 | 0.03 (0.17) | 0.11 (0.32) | 0.32 |
| Follow-up encounter | 0.02 (0.14) | 0.14 (0.35) | 0.46 | index month:7 | <0.01 (0.04) | 0.05 (0.21) | 0.3 |
| Body weight | 0.39 (0.49) | 0.61 (0.49) | 0.45 | index month:5 | 0.01 (0.07) | 0.06 (0.23) | 0.3 |
| Radiologic examination, chest, 2 views, frontal and lateral | <0.01 (0.01) | 0.09 (0.28) | 0.43 | influenza, live, intranasal, quadrivalent | <0.01 (0.01) | 0.04 (0.2) | 0.29 |
| Body height Measured | 0.1 (0.3) | 0.26 (0.44) | 0.43 | index month:4 | 0.01 (0.12) | 0.07 (0.26) | 0.29 |
| Influenza, injectable, quadrivalent, preservative free | 0.01 (0.08) | 0.1 (0.3) | 0.42 | Child examination | 0.04 (0.2) | 0.12 (0.33) | 0.29 |
| Body height [Percentile] | 0.02 (0.13) | 0.12 (0.32) | 0.41 | index month:6 | <0.01 (0.05) | 0.04 (0.19) | 0.25 |
| Child examination | <0.01 (0) | 0.08 (0.27) | 0.41 | Requires influenza virus vaccination | 0.03 (0.18) | 0.09 (0.28) | 0.22 |
| Tobacco smoking behavior - finding | 0.28 (0.45) | 0.47 (0.5) | 0.4 | index month:8 | 0.02 (0.15) | 0.07 (0.25) | 0.22 |
| Body mass index (BMI) [Ratio] | 0.39 (0.49) | 0.58 (0.49) | 0.39 | CT Cervical spine W contrast IV | <0.01 (0.01) | 0.02 (0.14) | 0.2 |
| Body temperature | 0.28 (0.45) | 0.47 (0.5) | 0.39 | Newborn screening panel American Health Information Community (AHIC) | <0.01 (0.01) | 0.02 (0.14) | 0.2 |
| pneumococcal conjugate vaccine, 13 valent | <0.01 (0.06) | 0.07 (0.26) | 0.37 | rotavirus, live, monovalent vaccine | <0.01 (0.05) | 0.02 (0.16) | 0.2 |
| Child weight centiles - finding | 0.01 (0.08) | 0.08 (0.27) | 0.36 | Newborn screening test results panel - DBS | <0.01 (0.01) | 0.02 (0.13) | 0.19 |
| Mucus [Presence] in Urine sediment by Light microscopy | 0.03 (0.17) | 0.12 (0.33) | 0.36 | CT Cervical spine WO contrast | <0.01 (0.01) | 0.02 (0.13) | 0.18 |
| Child height centile finding | 0.01 (0.08) | 0.08 (0.27) | 0.36 | Routine gynecologic examination | 0.01 (0.08) | 0.03 (0.17) | 0.17 |
| Computer-aided detection (computer algorithm analysis of digital image data for lesion detection) with further review for interpretation, with or without digitization of film radiographic images; screening mammography | <0.01 (0) | 0.06 (0.23) | 0.34 | DTaP-hepatitis B and poliovirus vaccine | 0.01 (0.09) | 0.03 (0.18) | 0.17 |
| Requires influenza virus vaccination | <0.01 (0.01) | 0.05 (0.23) | 0.34 | 0.5 ML vitamin K1 2 MG/ML Injection | <0.01 (0.01) | 0.01 (0.12) | 0.17 |
| Patient encounter procedure | 0.12 (0.33) | 0.25 (0.44) | 0.34 | Requires vaccination | 0.01 (0.12) | 0.04 (0.2) | 0.16 |
| Heart rate | 0.4 (0.49) | 0.57 (0.5) | 0.33 | Initial hospital or birthing center care, per day, for evaluation and management of normal newborn infant | <0.01 (0) | 0.01 (0.11) | 0.16 |
| Systolic blood pressure | 0.45 (0.5) | 0.6 (0.49) | 0.31 | Asthma | 0.02 (0.14) | 0.05 (0.21) | 0.16 |
| Diastolic blood pressure | 0.45 (0.5) | 0.6 (0.49) | 0.31 | Inpatient visit on day 0 | <0.01 (0.03) | 0.01 (0.12) | 0.15 |
| Immunization administration through 18 years of age via any route of administration, with counseling by physician or other qualified health care professional; first or only component of each vaccine or toxoid administered | <0.01 (0.04) | 0.05 (0.22) | 0.31 | General examination of patient | 0.01 (0.1) | 0.03 (0.17) | 0.15 |
| Body mass index (BMI) [Percentile] | 0.01 (0.08) | 0.06 (0.24) | 0.3 | pneumococcal conjugate vaccine, 13 valent | 0.07 (0.25) | 0.11 (0.31) | 0.15 |
| ibuprofen 20 MG/ML Oral Suspension | <0.01 (0.02) | 0.04 (0.21) | 0.3 | hepatitis B vaccine, pediatric or pediatric/adolescent dosage | 0.01 (0.09) | 0.03 (0.16) | 0.15 |
| Aspartate aminotransferase [Enzymatic activity/volume] in Serum or Plasma | 0.12 (0.32) | 0.23 (0.42) | 0.3 | Gastroesophageal reflux disease | <0.01 (0.06) | 0.02 (0.14) | 0.14 |
| 0.5 ML influenza A virus A/Hong Kong/4801/2014 (H3N2) antigen 0.03 MG/ML / influenza A virus A/Singapore/GP1908/2015 (H1N1) antigen 0.03 MG/ML / influenza B virus B/Brisbane/60/2008 antigen 0.03 MG/ML... | <0.01 (0.01) | 0.04 (0.2) | 0.3 | Computer-aided detection (computer algorithm analysis of digital image data for lesion detection) with further review for interpretation, with or without digitization of film radiographic images; screening mammography | 0.05 (0.22) | 0.08 (0.28) | 0.14 |
| Hemoglobin A1c/Hemoglobin.total in Blood by HPLC | <0.01 (0.02) | 0.04 (0.2) | 0.3 | Depressive disorder | 0.01 (0.1) | 0.03 (0.16) | 0.14 |
| index month:10 | 0.02 (0.16) | 0.09 (0.29) | 0.29 | erythromycin 0.005 MG/MG Ophthalmic Ointment | 0.01 (0.08) | 0.02 (0.14) | 0.13 |
| hepatitis A vaccine, pediatric/adolescent dosage, 2 dose schedule | <0.01 (0.01) | 0.04 (0.19) | 0.28 | Single live birth | 0.01 (0.09) | 0.02 (0.15) | 0.13 |
| Vaccination needed | 0.02 (0.13) | 0.08 (0.26) | 0.27 | Other puncture of vein | <0.01 (0.03) | 0.01 (0.1) | 0.12 |
| NDA020503 200 ACTUAT albuterol 0.09 MG/ACTUAT Metered Dose Inhaler [Proventil] | <0.01 (0.03) | 0.04 (0.19) | 0.27 | Epithelial cells.non-squamous [#/area] in Urine sediment by Microscopy low power field | 0.01 (0.09) | 0.02 (0.15) | 0.12 |
| Fever | 0.01 (0.1) | 0.06 (0.23) | 0.27 | Pre-surgery evaluation | <0.01 (0.05) | 0.01 (0.12) | 0.12 |
| Hemoglobin [Mass/volume] in Blood | 0.27 (0.44) | 0.39 (0.49) | 0.27 | Injection or infusion of other therapeutic or prophylactic substance | <0.01 (0.01) | 0.01 (0.09) | 0.12 |
| Hematocrit [Volume Fraction] of Blood by Automated count | 0.26 (0.44) | 0.39 (0.49) | 0.27 | Direct antiglobulin test.poly specific reagent [Presence] on Red Blood Cells | <0.01 (0.04) | 0.01 (0.1) | 0.12 |
| MCHC [Mass/volume] by Automated count | 0.26 (0.44) | 0.39 (0.49) | 0.26 | influenza A virus A/Kansas/14/2017 (H3N2) antigen 158000000 UNT/ML / influenza A virus A/Switzerland/3330/2017 (H1N1) antigen 158000000 UNT/ML / influenza B virus B/Colorado/06/2017 antigen 158000000 UNT/ML... | <0.01 (0.02) | 0.01 (0.09) | 0.12 |
| Erythrocyte distribution width [Ratio] by Automated count | 0.26 (0.44) | 0.39 (0.49) | 0.26 | Benign essential hypertension | <0.01 (0.06) | 0.02 (0.13) | 0.12 |
| Erythrocytes [#/volume] in Blood by Automated count | 0.26 (0.44) | 0.39 (0.49) | 0.26 | Inflammatory disorder of digestive tract | <0.01 (0.04) | 0.01 (0.11) | 0.12 |
| Leukocytes [#/volume] in Blood by Automated count | 0.26 (0.44) | 0.39 (0.49) | 0.26 | Pain in limb | 0.01 (0.08) | 0.02 (0.14) | 0.12 |
| MCH [Entitic mass] by Automated count | 0.26 (0.44) | 0.39 (0.49) | 0.26 | Coronary arteriosclerosis | <0.01 (0.04) | 0.01 (0.1) | 0.12 |
| MCV [Entitic volume] by Automated count | 0.26 (0.44) | 0.39 (0.49) | 0.26 | Long-term drug therapy | <0.01 (0.02) | 0.01 (0.09) | 0.12 |
| Requires vaccination | 0.01 (0.08) | 0.05 (0.22) | 0.26 | esomeprazole 40 MG Delayed Release Oral Capsule [Nexium] | 0.01 (0.09) | 0.02 (0.15) | 0.12 |
| Radiologic examination, chest; single view, frontal | <0.01 (0) | 0.03 (0.18) | 0.26 | esomeprazole 40 MG Injection [Nexium] | <0.01 (0.01) | 0.01 (0.08) | 0.11 |
| age group: 15-19 | 0.02 (0.14) | 0.07 (0.26) | 0.26 | Subsequent hospital care, per day, for evaluation and management of normal newborn | <0.01 (0) | 0.01 (0.08) | 0.11 |
| Needs influenza immunization | 0.01 (0.09) | 0.05 (0.22) | 0.26 | rotavirus, live, pentavalent vaccine | <0.01 (0.05) | 0.01 (0.11) | 0.11 |
|  |  |  |  | Abnormal heart beat | <0.01 (0.01) | 0.01 (0.08) | 0.11 |
| ***Optum EHR*** | | | | | | | |
| Higher proportion before vaccination | | | | Higher proportion before vaccination | | | |
| Basophils/100 leukocytes in Blood | 1.36 (1.17) | 0.69 (0.83) | -0.91 | index month:10 | 0.43 (0.66) | 0.11 (0.33) | -0.44 |
| Eosinophils/100 leukocytes in Blood | 1.27 (1.13) | 0.64 (0.8) | -0.47 | 0.5 ML influenza A virus A/North Carolina/04/2016 (H3N2) antigen 0.03 MG/ML / influenza A virus A/Singapore/GP1908/2015 (H1N1) antigen 0.03 MG/ML / influenza B virus B/Iowa/06/2017 antigen 0.03 MG/ML / influenza B virus B/Singapore/INFTT-16-0610/2016 a... | 0.62 (0.79) | 0.38 (0.61) | -0.24 |
| Monocytes/100 leukocytes in Blood | 1.13 (1.06) | 0.61 (0.78) | -0.46 | Glucose [Mass/volume] in Serum or Plasma | 1.03 (1.01) | 0.78 (0.88) | -0.19 |
| Lymphocytes/100 leukocytes in Blood | 1.02 (1.01) | 0.56 (0.75) | -0.42 | index month:9 | 0.2 (0.45) | 0.11 (0.32) | -0.17 |
| Calcium.ionized/Calcium.total corrected for albumin in Blood | 1.15 (1.07) | 0.67 (0.82) | -0.39 | index month:11 | 0.19 (0.43) | 0.1 (0.32) | -0.16 |
| Alkaline phosphatase.renal/Alkaline phosphatase.total in Serum or Plasma | 0.78 (0.88) | 0.4 (0.63) | -0.35 | Basophils/100 leukocytes in Blood | 0.98 (0.99) | 0.78 (0.88) | -0.15 |
| Albumin [Presence] in Urine | 0.82 (0.9) | 0.43 (0.66) | -0.34 | Influenza, injectable, quadrivalent, preservative free | 0.14 (0.37) | 0.07 (0.27) | -0.14 |
| Penicillin G potassium [Mass] of Dose | 1.18 (1.09) | 0.72 (0.85) | -0.34 | MCHC [Mass/volume] | 0.81 (0.9) | 0.64 (0.8) | -0.14 |
| Albumin/Protein.total in Serum or Plasma | 0.79 (0.89) | 0.43 (0.65) | -0.33 | Eosinophils/100 leukocytes in Blood | 0.9 (0.95) | 0.73 (0.85) | -0.14 |
| Sodium [Moles/volume] in Saliva (oral fluid) | 1.13 (1.06) | 0.7 (0.83) | -0.32 | Penicillin G potassium [Mass] of Dose | 1.01 (1.01) | 0.83 (0.91) | -0.14 |
| Chloride [Moles/volume] in Saliva (oral fluid) | 1.08 (1.04) | 0.66 (0.81) | -0.32 | Sodium [Moles/volume] in Saliva (oral fluid) | 0.98 (0.99) | 0.8 (0.89) | -0.14 |
| Globulin [Mass/volume] in Serum | 0.34 (0.59) | 0.13 (0.36) | -0.31 | A history with no personal factors and/or comorbidities that impact the plan of care; An examination of body system(s) using standardized tests and measures addressing 1-2 elements f | 0.02 (0.13) | <0.01 (0.01) | -0.13 |
| Leukocytes [#/volume] in Blood | 0.98 (0.99) | 0.59 (0.77) | -0.31 | visit_occurrence concept count during day -450 through -181 concept_count relative to index | 14.94 (15.18) | 12.22 (14.33) | -0.13 |
| Carbon dioxide [Partial pressure] in Blood | 1.02 (1.01) | 0.63 (0.79) | -0.31 | Calcium.ionized/Calcium.total corrected for albumin in Blood | 0.94 (0.97) | 0.77 (0.88) | -0.13 |
| Neutrophil Ab [Presence] in Serum | 0.66 (0.81) | 0.35 (0.59) | -0.3 | Patient encounter procedure | 0.69 (0.83) | 0.55 (0.74) | -0.13 |
| Platelets [#/volume] in Blood | 0.94 (0.97) | 0.58 (0.76) | -0.3 | Chloride [Moles/volume] in Saliva (oral fluid) | 0.91 (0.96) | 0.75 (0.87) | -0.12 |
| Aspartate aminotransferase [Presence] in Body fluid | 0.66 (0.81) | 0.37 (0.61) | -0.29 | Platelet mean volume [Entitic volume] in Blood | 0.67 (0.82) | 0.53 (0.73) | -0.12 |
| MCH [Entitic mass] | 0.91 (0.95) | 0.56 (0.75) | -0.29 | Platelets [#/volume] in Blood | 0.8 (0.9) | 0.65 (0.81) | -0.12 |
| Glucose [Mass/volume] in Serum or Plasma | 1.08 (1.04) | 0.69 (0.83) | -0.29 | Carbon dioxide [Partial pressure] in Blood | 0.87 (0.94) | 0.72 (0.85) | -0.12 |
| Radiologic examination, chest; 2 views | 0.1 (0.31) | 0.01 (0.09) | -0.28 | Prediabetes | 0.02 (0.14) | <0.01 (0.05) | -0.12 |
| MCHC [Mass/volume] | 0.9 (0.95) | 0.57 (0.75) | -0.27 | Lymphocytes/100 leukocytes in Blood | 0.36 (0.6) | 0.27 (0.52) | -0.12 |
| Neutrophil cytoplasmic Ab.perinuclear [Presence] in Serum | 0.45 (0.67) | 0.23 (0.48) | -0.27 | Immunization administration (includes percutaneous, intradermal, subcutaneous, or intramuscular injections); 1 vaccine (single or combination vaccine/toxoid) | 0.21 (0.46) | 0.14 (0.38) | -0.12 |
| MCV [Entitic volume] | 0.89 (0.94) | 0.57 (0.75) | -0.27 | MCH [Entitic mass] | 0.77 (0.88) | 0.64 (0.8) | -0.11 |
| 0.5 ML varicella zoster virus glycoprotein E, recombinant 0.1 MG/ML Injection [Shingrix] | 0.07 (0.27) | <0.01 (0.03) | -0.26 | Cotinine/Creatinine [Mass Ratio] in Urine | 0.78 (0.88) | 0.64 (0.8) | -0.11 |
| Cotinine/Creatinine [Mass Ratio] in Urine | 0.87 (0.94) | 0.56 (0.75) | -0.26 | MCV [Entitic volume] | 0.78 (0.88) | 0.65 (0.8) | -0.11 |
| Glucose [Mass/volume] in Serum or Plasma | 0.79 (0.89) | 0.49 (0.7) | -0.26 | Alkaline phosphatase.renal/Alkaline phosphatase.total in Serum or Plasma | 0.58 (0.76) | 0.46 (0.68) | -0.11 |
| Hematocrit [Volume Fraction] of Blood | 0.69 (0.83) | 0.42 (0.65) | -0.25 | Leukocytes [#/volume] in Blood | 0.8 (0.9) | 0.67 (0.82) | -0.11 |
| Erythrocytes [#/volume] in Blood | 0.78 (0.88) | 0.49 (0.7) | -0.25 | Hematocrit [Volume Fraction] of Blood | 0.37 (0.61) | 0.28 (0.53) | -0.11 |
| Erythrocytes [#/volume] in Blood | 0.42 (0.65) | 0.22 (0.47) | -0.25 | Glucose [Mass/volume] in Serum or Plasma | 0.68 (0.82) | 0.56 (0.75) | -0.11 |
| Alanine aminotransferase [Enzymatic activity/volume] in Serum or Plasma | 0.6 (0.77) | 0.36 (0.6) | -0.25 | Anion gap in Blood | 0.64 (0.8) | 0.52 (0.72) | -0.11 |
| Platelet distribution width [Ratio] in Blood | 0.69 (0.83) | 0.43 (0.66) | -0.24 |  |  |  |  |
| index month:6 | 0.11 (0.33) | 0.02 (0.15) | -0.24 |  |  |  |  |
| MCV [Entitic volume] | 0.16 (0.39) | 0.05 (0.22) | -0.24 |  |  |  |  |
| Hemoglobin [Mass/volume] in Blood | 0.67 (0.82) | 0.42 (0.65) | -0.24 |  |  |  |  |
| Lymphocytes/100 leukocytes in Blood | 0.43 (0.66) | 0.24 (0.49) | -0.24 |  |  |  |  |
| Hemoglobin [Mass/volume] in Blood | 0.48 (0.69) | 0.28 (0.52) | -0.23 |  |  |  |  |
| Triglyceride [Percentile] | 0.25 (0.5) | 0.11 (0.33) | -0.23 |  |  |  |  |
| Monocytes/100 leukocytes in Blood | 0.22 (0.47) | 0.09 (0.31) | -0.22 |  |  |  |  |
| Cholesterol.total/Cholesterol in HDL [Percentile] | 0.26 (0.51) | 0.12 (0.35) | -0.22 |  |  |  |  |
| Anion gap in Blood | 0.69 (0.83) | 0.45 (0.67) | -0.22 |  |  |  |  |
| Screening digital breast tomosynthesis, bilateral for primary procedure) | 0.09 (0.3) | 0.02 (0.14) | -0.21 |  |  |  |  |
| index month:5 | 0.11 (0.34) | 0.03 (0.18) | -0.21 |  |  |  |  |
| A history with no personal factors and/or comorbidities that impact the plan of care; An examination of body system(s) using standardized tests and measures addressing 1-2 elements f | 0.04 (0.2) | <0.01 (0.01) | -0.2 |  |  |  |  |
| CHADS2VASc | 2.01 (1.58) | 1.58 (1.39) | -0.2 |  |  |  |  |
| MCHC [Mass/volume] | 0.19 (0.44) | 0.09 (0.29) | -0.19 |  |  |  |  |
| Hematocrit [Volume Fraction] of Blood | 0.41 (0.64) | 0.25 (0.5) | -0.19 |  |  |  |  |
| Charlson index - Romano adaptation | 1.68 (2.57) | 1.06 (2.11) | -0.19 |  |  |  |  |
| Acute glomerulonephritis | 0.03 (0.18) | <0.01 (0.01) | -0.18 |  |  |  |  |
| Lower proportion before vaccination | | | | Lower proportion before vaccination | | | |
| Computer-aided detection (computer algorithm analysis of digital image data for lesion detection) with further review for interpretation, with or without digitization of film radiographic images; screening mammography (List separately in addition to code | <0.01 (0.04) | 0.07 (0.27) | 0.26 | Any visit on day 0 | 0.58 (0.76) | 1 (1) | 0.33 |
| Radiologic examination, chest, 2 views, frontal and lateral | 0.01 (0.09) | 0.08 (0.28) | 0.23 | index month:3 | 0.01 (0.08) | 0.12 (0.35) | 0.32 |
| index month:9 | 0.02 (0.14) | 0.09 (0.31) | 0.22 | Outpatient visit on day 0 | 0.56 (0.75) | 0.89 (0.94) | 0.27 |
| index month:10 | 0.03 (0.16) | 0.1 (0.32) | 0.22 | index month:4 | <0.01 (0.05) | 0.06 (0.25) | 0.24 |
| Physical therapy evaluation | <0.01 (0.01) | 0.04 (0.2) | 0.2 | index month:2 | 0.03 (0.16) | 0.1 (0.32) | 0.2 |
| age group: 10-14 | <0.01 (0) | 0.04 (0.2) | 0.2 | index month:7 | <0.01 (0.03) | 0.04 (0.21) | 0.2 |
| age group: 0-4 | <0.01 (0) | 0.04 (0.2) | 0.2 | index month:8 | 0.02 (0.14) | 0.08 (0.29) | 0.19 |
| age group: 5-9 | <0.01 (0) | 0.04 (0.19) | 0.19 | index month:5 | <0.01 (0.02) | 0.04 (0.19) | 0.19 |
| Child examination | <0.01 (0.03) | 0.04 (0.2) | 0.19 | Emergency room visit on day 0 | 0.01 (0.08) | 0.04 (0.21) | 0.17 |
| 0.5 ML Streptococcus pneumoniae serotype 1 capsular antigen diphtheria CRM197 protein conjugate vaccine 0.0044 MG/ML / Streptococcus pneumoniae serotype 14 capsular antigen diphtheria CRM197 protein... | 0.03 (0.17) | 0.09 (0.3) | 0.18 | index month:1 | 0.05 (0.23) | 0.1 (0.32) | 0.13 |
| Immunization administration through 18 years of age via any route of administration, with counseling by physician or other qualified health care professional; first or only component of each vaccine or toxoid administered | <0.01 (0.03) | 0.03 (0.17) | 0.16 | index month:6 | <0.01 (0.04) | 0.02 (0.15) | 0.13 |
| age group: 15-19 | <0.01 (0.07) | 0.04 (0.19) | 0.15 |  |  |  |  |
| Requires influenza virus vaccination | 0.03 (0.17) | 0.08 (0.28) | 0.15 |  |  |  |  |
| Radiologic examination, chest; single view, frontal | <0.01 (0.03) | 0.02 (0.15) | 0.14 |  |  |  |  |
| Periodic comprehensive preventive medicine reevaluation and management of an individual including an age and gender appropriate history, examination, counseling/anticipatory guidance/risk factor reduction interventions | <0.01 (0) | 0.02 (0.13) | 0.13 |  |  |  |  |
| index month:11 | 0.06 (0.24) | 0.11 (0.32) | 0.12 |  |  |  |  |
| pneumococcal conjugate vaccine, 13 valent | 0.02 (0.14) | 0.05 (0.22) | 0.12 |  |  |  |  |
| Immunization administration through 18 years of age via any route of administration, with counseling by physician or other qualified health care professional; each additional vaccine or toxoid component administered fo | <0.01 (0.01) | 0.01 (0.12) | 0.12 |  |  |  |  |
| Occupational therapy evaluation | <0.01 (0) | 0.01 (0.12) | 0.12 |  |  |  |  |
| Screening mammography, bilateral (2-view study of each breast) | <0.01 (0.01) | 0.01 (0.12) | 0.11 |  |  |  |  |
| influenza, injectable, quadrivalent, contains preservative | 0.01 (0.09) | 0.03 (0.17) | 0.11 |  |  |  |  |
| Computer-aided detection (computer algorithm analysis of digital image data for lesion detection) with further review for interpretation, with or without digitization of film radiographic images; diagnostic mammography (List separately in addition to code | <0.01 (0.01) | 0.01 (0.11) | 0.11 |  |  |  |  |
